# Coupling Far-Western Blotting with Peptide Microarrays Reveals Novel E‑Cadherin Spore-Surface Ligands in *Clostridioides difficile*

**DOI:** 10.1021/acs.jproteome.5c01166

**Published:** 2026-06-16

**Authors:** Osiris K. Lopez-Garcia, Marjorie Pizarro-Guajardo, Klaudia I. Kocurek, Yohannes H. Rezenom, Daniel Paredes-Sabja

**Affiliations:** † Department of Biology, 14736 Texas A&M University, College Station, Texas 77843, United States; ‡ Interdisciplinary Graduate Program in Genetics & Genomics, Texas A&M University, College Station, Texas 77843, United States; § Department of Chemistry, Texas A&M University, College Station, Texas 77843, United States

**Keywords:** C. difficile spores, spore adherence, E-cadherin, peptide microarray, far-western blotting, CotE, CdeM, CDIF27147_02282, CDIF27147_03838

## Abstract

*Clostridioides difficile* is an anaerobic spore-forming bacterium and the leading cause of nosocomial diarrhea. A major clinical challenge is *C. difficile* infection recurrence, affecting 20–30% of patients, mainly driven by spore persistence. The mechanisms underlying spore persistence in the gut remain poorly understood. Recently, our group showed that E-cadherin acts as a spore receptor mediating adherence to intestinal epithelial cells (PMID: 36448839), but the identity of the E-cadherin-binding proteins remains unknown. Here, far-Western blotting coupled with MS/MS-identified E-cadherin-binding spore ligands. The spore surface proteins CotE (CDIF27147_01458) and CdeM (CDIF27147_01682), along with two uncharacterized proteins, CDIF27147_03838 and CDIF27147_02282, interact with E-cadherin. Peptide microarray analysis mapped discrete E-cadherin-binding regions within these proteins, corresponding to 9–20 residue motifs, predicted to be surface-exposed by AlphaFold. However, competitive E-cadherin pull-down assays using synthetic peptides of these motifs did not reduce E-cadherin binding to *C. difficile* spores. Comparative genomics showed that these ligands and motifs are conserved across all five classical *C. difficile* clades (C1–C5). Similar levels of E-cadherin binding were observed in spores from all five classical clades. Collectively, this work identifies CDIF27147_03838 and CDIF27147_02282 as novel E-cadherin ligands and suggests additional roles for CotE and CdeM, expanding insights into spore-host interactions.

## Graphical abstract

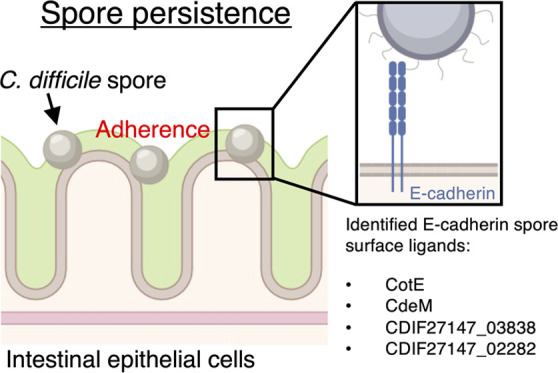

## Introduction

*Clostridioides difficile* is a strict anaerobic, spore-forming bacterium and the leading cause of nosocomial diarrhea worldwide.^1^ It is classified by the U.S. Centers for Disease Control and Prevention (CDC) as an “urgent threat” to the U.S. healthcare system.^2,3^ CDI causes over 220,000 cases annually, with associated healthcare costs approaching $4.8 billion and mortality rates of 5–8%.^2,3^ The incidence of *C. difficile* infection (CDI) is strongly associated with antibiotic usage, which disrupts the gut microbiota and creates conditions favorable for spore germination and bacterial proliferation.^2,4^ Current treatments rely primarily on antibiotics and, in recurrent cases, more antibiotics and/or fecal microbiota transplantation.^5,6^ However, antibiotics, while essential for CDI treatment, remain the primary risk factor for recurrence, which affects 20–30% of CDI patients and may increase to 60% after multiple episodes.^5,7^ Recurrent CDI (R-CDI) is a major clinical challenge,^2,7^ largely attributed to the persistence of metabolically dormant, aerotolerant spores in the host after therapy.^8^

*C. difficile* spores are the infectious and transmissible form of the bacterium and play a central role in disease recurrence and transmission.^8,9^ The exosporium, which is the outermost layer of *C. difficile* spores, serves as the first barrier that interacts with host intestinal surfaces.^9–13^ *C. difficile* produces two exosporium morphotypes: spores with a thick, electron-dense exosporium (often with polar appendages) and spores with a thin exosporium layer, both forming simultaneously during sporulation.^9,13–15^ This exosporium variability appears to be unique to *C. difficile* and may provide competitive fitness to occupy specific niches.^9,13,14^ The overall ultrastructural organization of *C. difficile* spores resembles that of other endospore-formers, yet only 25% of spore coat genes in *C. difficile* have homologues in *Bacillus subtilis*, highlighting the unique proteome of the surface of *C. difficile* spores.^9,16^ Early works revealed that *C. difficile* spores adhere to human intestinal enterocyte-like Caco-2 cells through interactions mediated by spore-surface and host cell proteins, with a binding preference to the basolateral membrane.^10^ Additionally, competitive binding assays in differentiated Caco-2 cells further suggests that spore-adherence is concentration-dependent and mediated by cell receptors.^11^ Electron micrographs support the notion that surface hair-like projections of *C. difficile* spores might mediate binding of spores to the host-cell membrane and apical microvilli of Caco-2 cells.^11^ However, the identity of the spore surface ligands remains unknown.

In the intestinal epithelium, tight junctions (TJs) and adherens junctions (AJs) form the apical junctional complex (AJC), safeguarding barrier integrity.^17,18^ E-cadherin, the core transmembrane protein of AJs, orchestrates cell–cell adhesion by linking to catenin and the actin cytoskeleton.^17,19,20^ Typically restricted to basolateral domains, E-cadherin can become accessible under specific conditions, such as in cells undergoing apoptosis at cell extrusion sites, along the lateral membranes of goblet cells and within epithelial folds of the intestinal villi that arise due to tissue tension and constriction forces.^21–23^ Both *C. difficile* toxins, TcdA and TcdB, function as glucosyltransferases that specifically modify Rho family GTPases, including RhoA, Rac1, and Cdc42, within host cells.^24,25^ This covalent modification inactivates their GTPase activity, leading to the disruption of the actin cytoskeleton and breakdown of tight and adherens junctions.^26–30^ As a result, these toxins compromise the epithelial barrier and increase paracellular permeability.^31^ In the context of CDI, toxins TcdA and TcdB induce redistribution of E-cadherin increasing its accessibility in apical and basal surfaces of intestinal epithelial cells.^32^ Enhanced E-cadherin accessibility during CDI is linked to increased adherence of *C. difficile* spores, especially those with a thick exosporium, to the disrupted epithelial barriers and preferentially to basolateral membranes.^32^

In most clinically relevant *C. difficile* strains, including strain R20291,^33,34^ the outermost exosporium layers has hair-like projections formed by members of the BclA family of collagen-like proteins.^9,16,35,36^ Electron micrographs coupled with immunogold-labeling demonstrates that E-cadherin localizes with these hairs.^32^ However, single *bclA*-gene inactivation does not affect E-cadherin binding,^32^ suggesting that unidentified ligands contribute to this interaction. Proteomic analyses revealed the presence of 184 proteins in the exosporium layer of *C. difficile* 630 spores;^37,38^ of which 8 are accessible to antibodies, suggesting their potential implication in spore-host interactions.^12,13,38–42^ Among these antibody-accessible are the spore-coat proteins CotA and CotB that have minor roles in spore coat and exosporium integrity,^38^ yet their roles in pathogenesis remain unknown. CotE is a spore coat protein, a bifunctional enzyme formed by a peroxiredoxin N-terminal domain and a Chitinase C-terminal domain,^37,41,42^ that promotes mucin binding and contributes to colonization.^40^ Among accessible exosporium proteins are two cysteine-rich morphogenetic proteins, CdeC and CdeM,^12,37^ essential for exosporium formation, and *cdeC* and *cdeM* spores are affected in adherence to the intestinal mucosa and pathogenesis.^12^ Despite significant progress in characterizing composition and assembly of the exosporium, the identity of spore ligand(s) responsible for interactions with E-cadherin remains unknown.

This work aimed to identify ligands for binding of E-cadherin to *C. difficile* spores. Using far-Western blotting coupled to mass spectrometry, we identified 34 protein candidates of which 13 were selected to assess binding of recombinant protein to E-cadherin. Far-Western blot analysis of selected recombinant proteins revealed four spore proteins (CotE, CdeM, CDIF27147_03838, and CDIF27147_02282) as E-cadherin ligands. The peptide microarray revealed discrete E-cadherin binding motifs ranging from 9 to 20 residues in these proteins. Sequence comparison showed that both full-length proteins and their corresponding motifs are highly conserved across the five classical *C. difficile* clades. Consistently, E-cadherin pull-down assays showed similar binding to spores from strains representing each clade. However, synthetic peptides derived from these motifs did not inhibit E-cadherin binding to spores, suggesting that multiple motifs participate in the redundant binding of E-cadherin to the spore. Altogether, this work narrows the candidates for *C. difficile* E-cadherin ligands to four conserved spore proteins, providing a foundation for future genetic and functional studies to determine their roles in spore adherence and as potential targets for disrupting spore persistence and disease recurrence.

## Materials and Methods

### Bacterial Strains and Growth Conditions

*C. difficile* strains (Table S1) were grown at 37 °C under anaerobic conditions in a Coy anaerobic chamber (4% H_2_, 5% CO_2_, 85% N_2_) in BHIS medium: 3.7% (w/v) brain heart infusion broth supplemented with 0.5% (w/v) yeast extract (Difco) and 0.1% (w/v) l-cysteine or on 3.7% (w/v) BHIS agar plates. *Escherichia coli* strains (Table S1) were grown aerobically at 37 °C under aerobic conditions with shaking at 150 rpm in a Luria–Bertani medium (BD), supplemented with 100 μg/mL ampicillin (Sigma), where appropriate.

### Purification of *C. difficile* Spores

*C. difficile* was cultured anaerobically on BHIS and 70:30 media to induce sporulation. After 5 days, spores were isolated by repeated ice-cold water washes and overnight lysis of residual vegetative cells, achieving >99% purity as verified by microscopy. Spores were quantified by a hemocytometer and stored at −80 °C for subsequent assays (extended methods in the Supporting Information).

### E-Cadherin Pull Down Binding Assay with *C. difficile* Spores

*C. difficile* R20291 spores were incubated with recombinant human E-cadherin (Advanced Biomatrix, Cat no. 5085) at 1–20 μg/mL, then washed, and subjected to immunoblot analysis. Binding was detected using rabbit anti-E-cadherin (Abcam, Cat no. AB40772) and HRP-conjugated goat antirabbit IgG (Thermo Fisher, Cat no. 31460). Band intensity was quantified by densitometry (ImageJ), and dissociation constant (*K*_d_) values were calculated from saturation binding curves. Comparative assays of spores from all five classical clades used identical incubation and detection protocols (extended methods in the Supporting Information).

### Fluorescence Microscopy of E-Cadherin-Binding to *C. difficile* Spores

*C. difficile* spores (1 × 10^8^ in 20 μL) were incubated with recombinant human E-cadherin Advanced Biomatrix, Cat no. 5085 at 0–8 μg/mL. After washing, spores were blocked and incubated overnight with rabbit anti-E-cadherin (Abcam, AB40772), followed by incubation with Alexa Fluor 568 antirabbit IgG (Abcam, AB175692, 1:400). Labeled spores were washed, air-dried on coverslips and mounted with ProLong Diamond antifade (Invitrogen). Images were acquired by fluorescence microscopy and analyzed using Fiji/MicrobeJ with standardized segmentation and background-corrected intensity measurements (extended methods in the Supporting Information).

### One-Dimensional Far-Western Blot

Spore coat/exosporium extracts were prepared with CHAPS buffer, separated by SDS-PAGE and transferred to a nitrocellulose membrane. Membranes were blocked in 3% BSA in TBST and incubated with 1 μg/mL recombinant human E-cadherin (Advanced Biomatrix, Cat no. 5085). Detection used a rabbit anti-E-cadherin primary antibody (Abcam, Cat no. AB40772), followed by a HRP-conjugated goat antirabbit IgG secondary antibody (Thermo, Cat no. 31460), and visualized through chemiluminescence (extended methods in the Supporting Information).

### Two-Dimensional (2D) Far-Western Blot and LC-MS/MS

Spore coat/exosporium extracts were resolved by isoelectric focusing followed by electrophoresis in SDS-PAGE and then transferred to a nitrocellulose membrane. Far-Western blotting for E-cadherin binding and antibody detection was performed as in the one-dimensional method. Immunoreactive spots were excised and subjected to in-gel trypsin digestion, with protein identification by LC–MS/MS (extended methods in the Supporting Information).

### Cloning of Ligand Candidates into Overexpressing pET-16b Vectors

Candidate ligand genes from *C. difficile* R20291 were PCR-amplified and cloned into pET-16b vectors using NdeI and *Bam*HI restriction sites, resulting in N-terminal His-tagged constructs. Plasmids were sequenced by Oxford Nanopore sequencing prior to transformation (extended methods in the Supporting Information).

### Heterologous Overexpression of Ligand Candidates in *E. coli*

*E. coli* BL21­(DE3) cells were transformed with sequence-confirmed pET-16b constructs, grown to mid log phase, and protein expression induced with 1 mM IPTG. Soluble and insoluble fractions were isolated by sonication and centrifugation, with His-tagged proteins confirmed by immunoblotting (extended methods in the Supporting Information).

### One Dimensional Far-Western Blot of Recombinant Candidate Proteins with Human E-Cadherin

Soluble and insoluble fractions and empty vector controls were separated by SDS-PAGE, transferred to nitrocellulose, and blocked with 3% BSA. Membranes were incubated with 1 μg/mL recombinant E-cadherin, followed by rabbit anti-E-cadherin (Abcam, AB40772) and HRP-conjugated goat antirabbit IgG (Thermo, 31,460), with chemiluminescence detection. For His-tagged protein detection, membranes were probed with a mouse anti-6xHis antibody (Invitrogen, MA1-21,315) and HRP-conjugated goat antimouse IgG (Rockland, 610-1302) before imaging (extended methods in the Supporting Information).

### Amino Acid Conservation of E-Cadherin Ligand Candidates

A small database was produced to include 250 *C. difficile* isolates, with 50 from each of the five classical clades, extracted from the published data set. Candidate ligand protein sequences were analyzed across these genomes by tBLASTn in Geneious Prime, reporting pairwise identity and motif-level conservation (extended methods in the Supporting Information).

### Peptide Microarray Assay

Conformational peptide microarrays (PEPperPRINT, Germany) were designed as overlapping 9-mer and 13-mer peptides from six candidate proteins, each flanked by GSGSGSG linkers and printed in duplicate (2972 peptides total). Arrays were probed with recombinant GST-tagged human E-cadherin (Antibodies Online, ABIN1348882) at 1 or 10 μg/mL and stained with anti-GST DyLight680 and anti-HA DyLight800 antibodies. Fluorescence was quantified using PepSlide Analyzer software, with data quality and motif mapping performed as described (extended methods in the Supporting Information).

### AlphaFold3 Prediction and Biophysical Properties of Ligands

Ligand protein sequences were extracted from the *C. difficile* R20291 genome and modeled by AlphaFold3 (single-chain mode, default settings). Top models were visualized in ChimeraX for mapping predicted E-cadherin-binding sites, electrostatics, and hydrophobicity. Biophysical properties (pI, net charge, and hydropathy) were calculated using Prot pi and ProtScale. Intrinsic disorder predictions were performed with DISOPRED3 in PSIPRED (extended methods in the Supporting Information).

### Inhibitory E-Cadherin Pull Down Binding Assay with *C. difficile* R20291 Spores and Synthetic Peptides

Peptides matching high-affinity E-cadherin binding motifs (9–20 aa; identified by microarray) and scrambled controls were custom synthesized (Alan Scientific, USA). Recombinant E-cadherin (Advanced Biomatrix; Cat no. 5085) was mixed with peptides (1–15 μM) and incubated with 1 × 10^8^ spores. After washing, E-cadherin binding was quantified by immunoblotting with rabbit anti-E-cadherin (Abcam, AB40772) and goat antirabbit HRP (Thermo, 31,460), using chemiluminescent detection (extended methods in the Supporting Information).

### Statistical Analyses

All data were analyzed in GraphPad Prism 10. Dose–response curves were fit by nonlinear regression to estimate *K*_d_. Group comparisons used one-way ANOVA with Šídák’s or Tukey’s tests, or Welch’s t-test for two groups. Normality was checked by Q–Q plots. *P* < 0.05 was considered significant (extended methods in the Supporting Information).

## Results

### E-Cadherin Binds to *C. difficile* Spores with Nanomolar Affinity

E-cadherin acts as a spore-adherence receptor, enhancing binding of *C. difficile* spores to intestinal epithelial cells, increased by toxins TcdA and TcdB.^32^ Although E-cadherin binding to *C. difficile* spores is concentration dependent, the binding affinity (*K*_d_) was not determined. Therefore, we revisited these interactions by pull down and single spore analyses of E-cadherin-fluorescently labeled *C. difficile* spores. For this, we used spores from a highly sporulating derivative of strain R20291, denominated R20291_CM196_ strain. The R20291_CM196_ strain has a single nucleotide polymorphism in the *rsbV* gene that leads to a hyper-sporulating phenotype, allowing high spore yields.^43^ None of the core sporulation-associated genes are affected and ultrastructural analyses confirm that R20291_CM196_ spores are similar to the parental strain.^43^ The pull-down assay with increasing concentrations of recombinant E-cadherin (1–6 μg/mL, ∼17–99 nM) incubated with intact *C. difficile* spores resulted in progressively stronger immunoreactive bands at ∼70 kDa (Figures 1A, S1 and S2). Under these conditions, the detected ∼70 kDa species corresponds to the recombinant E-cadherin construct used in this study (residues 155–710; theoretical mass ∼60.75 kDa, migrating at ∼70 kDa in SDS-PAGE), which is pulled down via its interaction with surface-exposed spore ligands, rather than to the ligands themselves. At concentrations above 6 μg/mL (99 nM), the ∼70 kDa band intensity continued to increase and became oversaturated at higher concentrations (Figures 1A, S1 and S2). In the gels, a weaker band at ∼35 kDa was occasionally detected; because its identity is uncertain and it was not consistently observed, we focused our analysis on the ∼70 kDa band corresponding to recombinant E-cadherin. In our SDS-PAGE, a predominant band at ∼20 kDa is included as a loading control (Figures 1A, S1 and S2). The prominent ∼20 kDa band corresponds to a highly abundant spore protein that appears as the most prominent band, identified as the exosporium CdeM.^44^ Because its intensity is stable between lanes and independent of E-cadherin treatment, this band was used as an internal loading control to verify equivalent spore protein input across lanes. Thus, Figure 1A reports the affinity of recombinant E-cadherin to intact *C. difficile* spores; the identity of the underlying spore ligands is addressed in Figure 2 using extracted coat/exosporium proteins.

**Figure 1:**
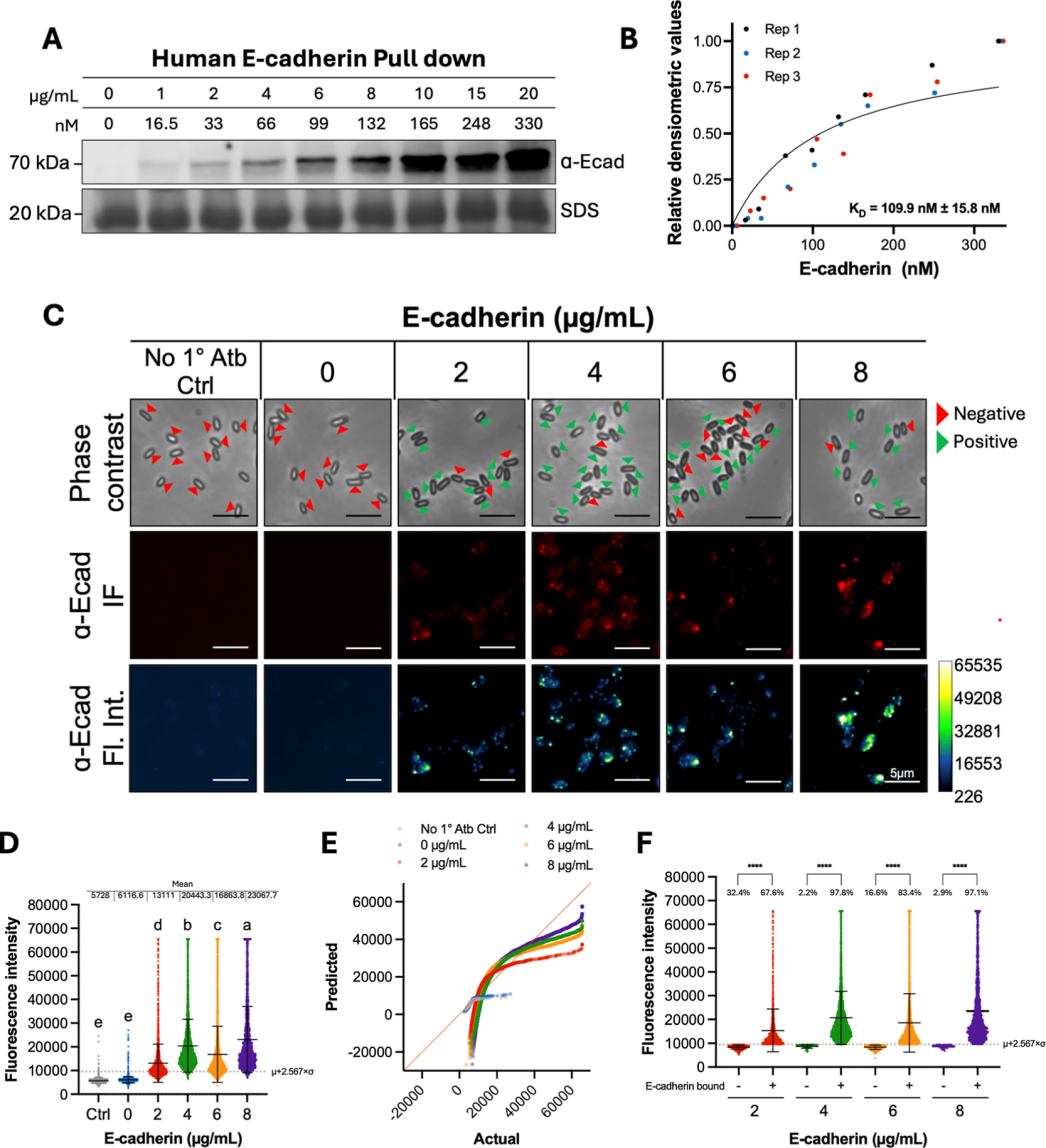
Quantitative binding and detection of E-cadherin interaction with *C. difficile*R20291_CM196_ spores. (A) E-cadherin pull down assay showing binding of recombinant human E-cadherin to *C. difficile* R20291_CM196_ spores. Spores (1 × 10^8^) were incubated with increasing concentrations of E-cadherin (0–20 μg/mL), washed, and probed with an anti-E-cadherin antibody; SDS-PAGE of input is shown as a loading control. (B) Binding saturation analysis for E-cadherin-spore interactions. Densitometric values from replicate experiments were fit to a single-site binding model to calculate the dissociation constant (*K*_d_ = 109.9 nM ± 15.8 nM). (C) Representative phase-contrast and immunofluorescence images of *C. difficile* spores including the control sample incubated only with an Alexa Fluor 568 secondary antibody (No 1° Atb Ctrl) and samples incubated with 0, 2, 4, 6, or 8 μg/mL of E-cadherin, labeled with an anti-E-cadherin antibody and Alexa Fluor 568 secondary antibody; fluorescence intensity maps are shown below. Scale bar, 5 μm. Red arrows, non-E-cadherin-bound (negative) spores. Green arrows, E-cadherin-bound (positive) spores. (D) Fluorescence intensity quantified per spore for each experimental condition in one replicate, analyzed with MicrobeJ in ImageJ. Scatter plots show distribution of fluorescence intensities for each treatment; mean is shown from each condition, and statistical significance was determined using Šídák’s multiple comparison test. Groups not sharing a letter are significantly different (*P* < 0.05) as determined by multiple comparison tests. Groups sharing one or more letters are not significantly different. Threshold for E-cadherin bound spores was calculated as μ + 2.576 × σ (9525.1) using values from E-cadherin 0 μg/mL and is shown as a gray dotted line. For the single-spore immunofluorescence analyses, five microscopic fields were analyzed per biological replicate, with a minimum of 1000 spores per replicate; across both replicates, at least 10 fields and >3000 spores were analyzed in total per condition. (E) Normal Q–Q plot comparing predicted versus actual values of E-cadherin fluorescence intensity across samples treated with varying concentrations (0–8 μg/mL) and no primary antibody control. Each sample group is represented by a distinct color as indicated in the legend. The dashed red line denotes the reference for perfect agreement between predicted and actual values. Deviations from the line indicate departures from normality for each condition. (F) Quantification of E-cadherin fluorescence intensity per spore across samples in one replicate divided based on whether spores were negative or positive for E-cadherin binding, statistical significance was determined using the Welch’s *t*-test (****, *P* < 0.0001). Threshold for E-cadherin bound spores was calculated as μ + 2.576 × σ and is shown as a gray dotted line. Percentages correspond to the proportion of E-cadherin-bound positive or negative spores out of total spores.

**Figure 2:**
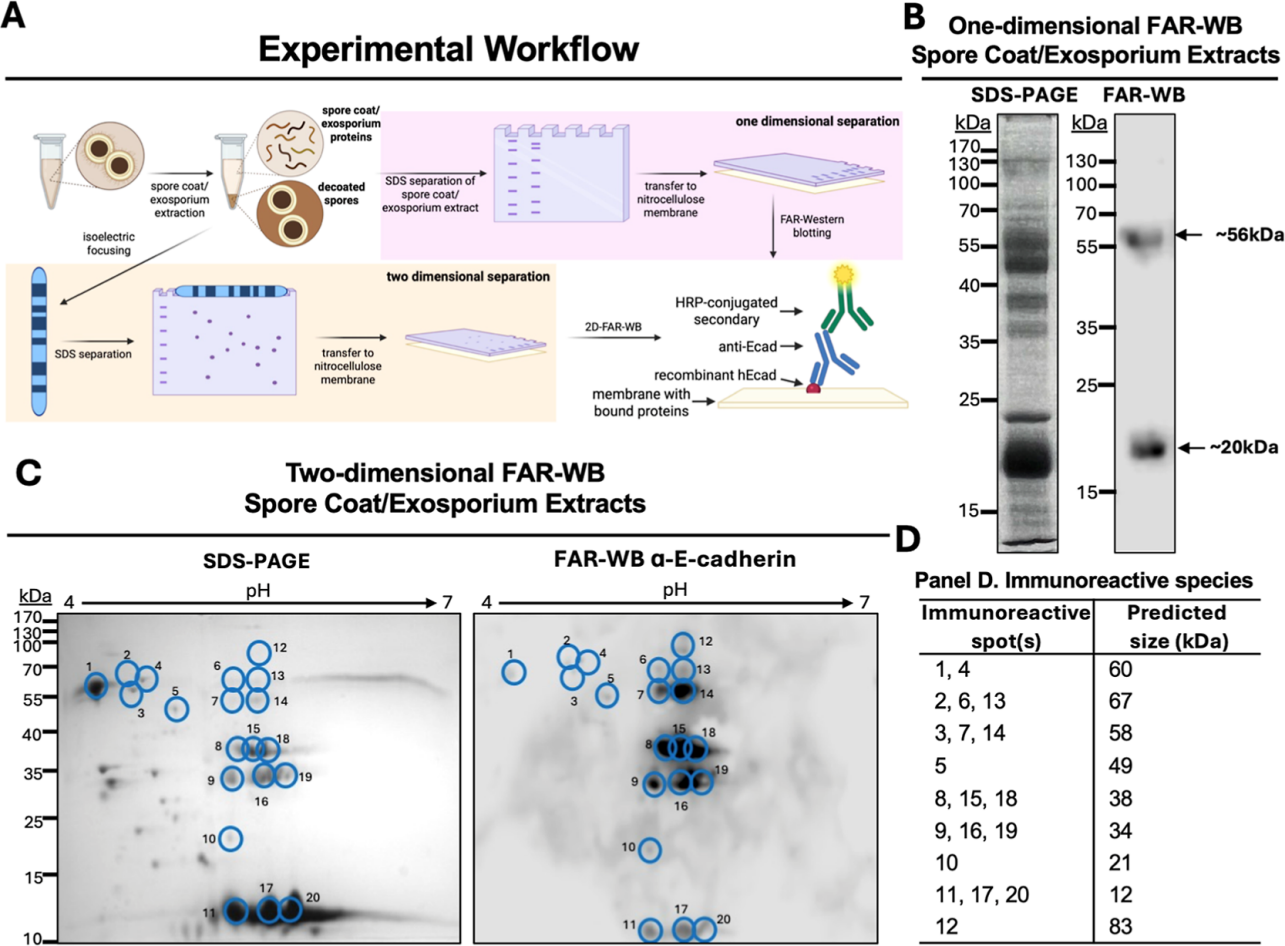
Identification of novel *C. difficile* R20291_CM196_ spore surface proteins that bind E-cadherin by FAR-Western immunoblotting analysis. (A) Schematic overview of the experimental workflow for extracting spore coat/exosporium proteins and analyzing E-cadherin binding by one-dimensional and two-dimensional FAR-Western blotting. (B) One-dimensional separation of spore coat/exosporium protein extracts. SDS-PAGE with Coomassie blue staining is shown on the left. The FAR-Western blot, probed with recombinant human E-cadherin, is shown on the right, revealing two major immunoreactive bands at approximately 56 kDa and 20 kDa. (C) Two-dimensional protein separation and detection of E-cadherin binding proteins. The left panel shows 2D-SDS-PAGE with Coomassie blue staining, and the right panel displays the corresponding 2D FAR-Western blot with immunoreactive spots (numbered 1–20) detected using an anti-E-cadherin antibody. Molecular weight markers (kDa) and pH gradients (4–7) are indicated. (D) Predicted molecular weights (kDa) for immunoreactive protein spots identified in the two-dimensional FAR-Western blot analysis.

To determine the binding affinity of E-cadherin to spores, E-cadherin was pull down with *C. difficile* spores (Figures 1A, S1 and S2), and the resulting HRP-detected blot bands were analyzed by densitometry. For each lane representing E-cadherin concentration, raw densitometric values were background-corrected by subtracting the signal from the 0 μg/mL control lane within each replicate, and adjusted values were relativized to the maximum value observed in their respective replicate, which corresponded to the 20 μg/mL E-cadherin concentration in all replicates, ensuring comparability across gels. Next, relativized densitometric values were fitted using a standard saturation binding model to estimate the dissociation constant (*K*_d_),^11,45^ yielding an average dissociation constant (*K*_d_) of 109.9 nM ± 15.8 nM (Figures 1B, S1 and S2), consistent with a moderate nanomolar affinity observed in receptor–ligand interactions.^46^

### Single-Spore Immunofluorescence Reveals Heterogeneous and Bimodal E-Cadherin Binding

Previous work revealed that E-cadherin binding to *C. difficile* spores is heterogeneous, and fluorescence is unevenly distributed around the spore.^32^ To thoroughly address this differential binding, we incubated spores with increased concentrations of E-cadherin which were then immunostained and analyzed by fluorescence microscopy at a single-spore level. In both negative controls (no primary antibody and 0 μg/mL E-cadherin), the fluorescence signal is minimal or absent, establishing a background baseline (Figure 1C). At 2 μg/mL, a subset of spores displayed detectable spotted immunofluorescence, suggesting a nonhomogeneous ligand distribution on the spore surface (Figure 1C). As the E-cadherin concentration increased to 4 and 6 μg/mL, the immunofluorescence became stronger but remained uneven around the spore surface, with signals intensifying further at 8 μg/mL (Figure 1C). However, even at the highest tested concentrations, only a subpopulation of spores showed high immunoreactivity (Figure 1C). This raises the hypothesis that there is differential tropism or variable surface accessibility of E-cadherin ligands among *C. difficile* spore populations.

To test the hypothesis, we performed single spore analysis to quantify fluorescence intensity across individual spores (Figures 1D andS1C). Single spore analysis of raw fluorescence intensities reveals that the mean fluorescence intensity for negative control was 5728 ± 911.6 (Figures 1D and S1C). For 0 μg/mL E-cadherin concentration, there was a slight but not statistically significant increase to 6116.6 ± 1323 (Figures 1D and S1C). However, a significant increase in the fluorescence intensity was observed at 2 μg/mL, which increased to 13,111 ± 8075 and to 2044.3 ± 11,173, 16863.8 ± 11,813, 23067.7 ± 13,998 at 4, 6, and 8 μg/mL, respectively (Figures 1D and S1C). Examination of the distribution of fluorescence (Figure S1D,E) shows a skewed distribution, supporting the presence of two spore populations, with the enriched population corresponding to E-cadherin-positive spores. Single-spore intensity analysis revealed that most spores exhibited fluorescence intensity between 8000 and 16,000, with the highest proportions at 4 μg/mL (34.7%) and 6 μg/mL (36.8%) (Figure S1D,E). A minority of spores incubated with 4, 6, and 8 μg/mL displayed mean fluorescence intensity up to 64,000, representing 20.7%, 24.8%, and 23.3% of those populations, respectively, and supporting the presence of two spore subpopulations. A lack of normality was evidenced by conducting a probability distribution analysis (Figures 1E and S1F). Results demonstrate that there are strong deviations from the reference line particularly at higher fluorescence values at 2, 4, 6, and 8 μg/mL, suggesting that the data do not follow a normal or Gaussian distribution (Figures 1E and S1F). Furthermore, normality hypothesis tests were performed for each concentration group, and all yielded significant *P*-values (*P* < 0.001 for each group), indicating strong evidence against normality.

The observation of one group with low fluorescence intensities and another with higher intensities provides statistical support for the existence of distinct E-cadherin-reactive spore populations. To separate these populations as negative or positive for E-cadherin binding, a threshold was established by calculating the upper 95% confidence limit of the control (0 μg/mL E-cadherin) population for each experimental replicate. This threshold was calculated using the mean (μ) and standard deviation (σ) of the raw fluorescence intensities for control spores (E-cadherin 0 μg/mL) for each replicate and defined as μ + 2.576 × σ (Replicate 1 = 2877.7 + (2.576 × 707) and Replicate 2 = 6116.6 + (2.576 × 1323.2)) (Figures 1D,F and S1C,G). Using this threshold, we separated the spores as positive and negative for E-cadherin binding (Figures 1F and S1G). Analysis revealed that at each concentration, there is a significant increase in fluorescence intensity, with means for negative spores being 8336 ± 852.3, 8722 ± 728.6, 8336 ± 902.2, and 8756 ± 610.9 at 2, 4, 6, and 8 μg/mL, respectively, and increasing to 15,397 ± 8941, 20,702 ± 11,157, 18,564 ± 12,240, and 23,499 ± 13981.1 at 2, 4, 6, and 8 μg/mL, respectively, for positive spores (Figure 1F). Differences between negative and positive spores for all concentrations were significant (*P* < 0.0001), suggesting that there are high- and low-binding spore subpopulations (Figures 1F and S1G). At 2 μg/mL, 67.8% of spores were E-cadherin-bound. This percentage increased further at higher concentrations, reaching 97.8% at 4 μg/mL, 97.1% at 6 μg/mL, and 97.9% at 8 μg/mL (Figure 1F). Altogether, these results demonstrate E-cadherin binding to *C. difficile* spores is heterogeneous, exhibiting a bimodal binding phenotype.

### Two-Dimensional Far-Western Blotting Identifies Spore Ligands for E-Cadherin

Although *C. difficile* spores interact with E-cadherin,^32^ the identity of spore ligands remains unknown. As a first approach to test the hypothesis that spore ligands are present in the spore surface, we extracted coat/exosporium proteins from R20291_CM196_ spores, which were resolved by electrophoresis in SDS-PAGE and transferred to nitrocellulose for subsequent incubation with recombinant human E-cadherin and anti E-cadherin antibody followed by the HRP-conjugated secondary antibody (Figure 2A). Unlike the pull-down assay in Figure 1A, where the immunoreactive ∼70 kDa band corresponds to recombinant E-cadherin bound to intact spores, the far-western experiments use E-cadherin as a probe to detect its receptors within spore coat/exosporium extracts. In this format, the immunoreactive bands and spots represent spore proteins that serve as E-cadherin ligands, not the E-cadherin probe itself. Results demonstrate that E-cadherin specifically interacted with two immunoreactive species of predicted sizes of ∼56 kDa and ∼20 kDa (Figure 2B), suggesting the presence of ligands for E-cadherin within extracts of spore coat and exosporium proteins.

However, since these one-dimensional E-cadherin-reactive bands are likely to contain multiple spore coat/exosporium proteins, we increased the resolution by separating the coat and exosporium extraction mixture by the charge and size. For this coat/exosporium, extracts were separated by charge further analyzed by isoelectric focusing (pH 4–7) followed by electrophoresis in a SDS-PAGE and far-Western blotting against E-cadherin (Figure 2A). Results identified 17–20 spots that were recognized by E-cadherin (Figures 2C and S3). These spots spanned isoelectric points from pH 4.0 to 5.5, with a predominant cluster at pH 5.5, specifically spots 6 to 20 localized around this pH (Figures 2C and S3). Apparent molecular weights ranged from 12.5 to 85 kDa, with ten spots being in the 50 and 85 kDa range, six between 34 to 38 kDa, and four between 12.5 to 20 kDa (Figure 2D). The most intense immunoreactive spots correspond to species with molecular weights of 55 kDa (spots 7 and 14), 38 kDa (spots 8, 15, and 18), and 34 kDa (spots 9, 16, and 19), consistent with the major ∼56 kDa and ∼20 kDa bands and additional, partially overlapping species that are not resolved in the 1D gel (Figure 2C,D). Replica far-Western blots also identified 17–18 E-cadherin immunoreactive spots each with similar molecular weight and isoelectric profile patterns (Figure S3). Minor variability between replicates was observed regarding the immunoreactive intensity for those spots that reacted with E-cadherin, suggesting modest heterogeneity in the far-Western blots (Figure S3). Altogether, these results demonstrate the presence of multiple proteins within the coat and exosporium extracts that seem to be ligands for E-cadherin in *C. difficile* R20291 spores.

To identify the protein species in these E-cadherin-reactive spots, each spot was excised from a mirror 2D SDS-PAGE gels, digested with trypsin and analyzed by MS/MS (Figures 2C and S3). Mass spectrometry results from three biological replicates detected 29 to 143 proteins hits per spot, reflecting the expected comigration of multiple proteins within individual 2D gel features in spore coat/exosporium extracts. In total, this discovery-mode analysis identified 276 unique proteins passing a 1% FDR threshold and standard Proteome Discoverer reporting criteria (Table S4). To enrich for high-confidence E-cadherin ligand candidates, we then applied a stepwise filtering strategy before the downstream analyses shown in Figure 3 and Table S5. First, only proteins detected with more than two unique peptides were retained. Second, for each spot, we required that the predicted molecular assay of the protein fall within 10 kDa of the apparent molecular weight of the excised gel spot. Only proteins meeting both criteria were considered for ranking, and from these, the top 10 identifications were selected per spot (Figure 3A and Table S5). Application of this filtering pipeline reduced the data set from 276 to 154 unique protein hits across the three replicates. Of these, 25 proteins were classified as being under the control of the sporulation-specific RNA polymerase sigma factors Sigma E (σ^E^) and/or Sigma K (σ^K^), and two of these σ^E^/σ^K^-regulated proteins were detected in all three replicates (Figure 3A,B and Table S5).^47,48^ Among the 25 σ^E^ and/or σ^K^ regulated proteins, 13 have previously being localized to the exosporium or spore coat (Figure 3C,D).^9,37,49^ These results suggest that several spore coat and exosporium proteins, regulated by σ^E^ and/or σ^K^, are likely candidates for E-cadherin binding ligands in *C. difficile*.

**Figure 3:**
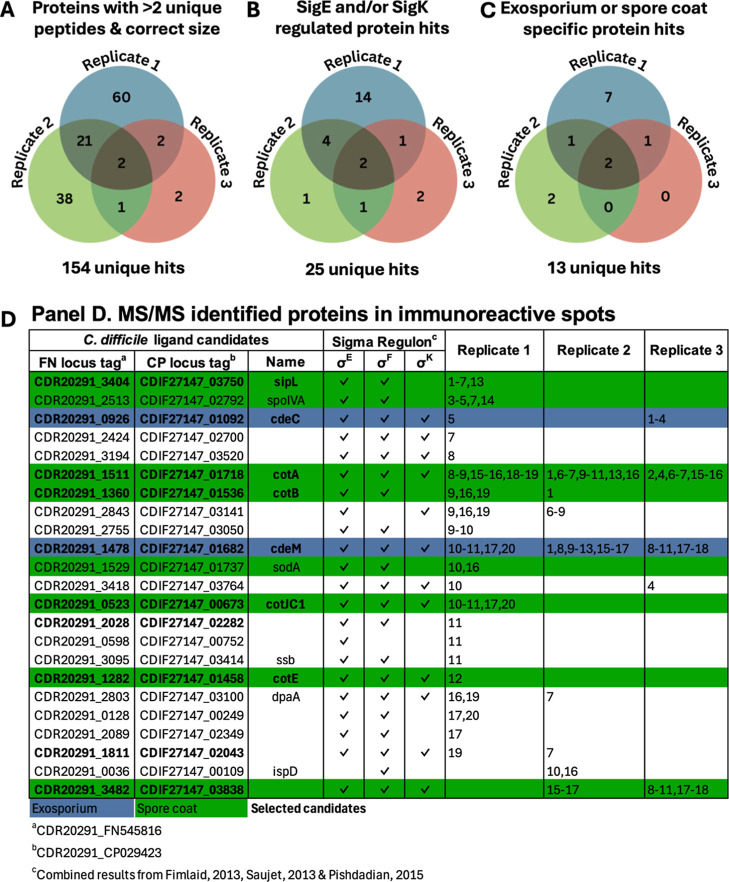
Overlap and identification of E-cadherin-binding proteins in *C. difficile* R20291_CM196_ spore coat/exosporium extracts across replicate analyses. (A–C) Venn diagrams displaying an overlap of mass spectrometry-identified proteins detected in E-cadherin immunoreactive spots from three biological replicates of 2D FAR-Western blots after sequential filtering. (A) Proteins retained after requiring more than two unique peptides, a predicted molecular mass within 10 kDa of the size of the excised spot apparent molecular weight, and selection among the top 10 ranked identification per spot. (B) Subset of filtered proteins regulated by sigma factors σ^E^ and/or σ^K^. (C) Subset of filtered proteins previously localized to the exosporium or spore coat. (D) Summary table of exosporium/spore coat proteins detected in immunoreactive spots, listing only those regulated by at least one of sigma factors σ^E^, σ^F^, or σ^K^. Table includes locus tags, gene names, sigma factor regulon assignments, and the corresponding immunoreactive spots for each biological replicate. Highlighted rows indicate selected candidate or previously characterized spore surface proteins.

### Recombinant CotE, CdeM, CDIF27147_02282, and CDIF27147_03838 Interact with E-Cadherin

A set of 13 spore coat and exosporium proteins, detected in immunoreactive spots by MS/MS, were selected for binding validation. Candidates chosen for recombinant validation were selected by applying the following criteria to the filtered MS data set: (i) the protein passed the stringent MS/MS filtering pipeline described above, (ii) the protein was regulated by at least one sporulation-specific sigma factor (σ^E^, σ^F^, or σ^K^), and/or (iii) had prior evidence of localization to the spore coat or exosporium or predicted structural, enzymatic, or assembly roles in these surface layers. Together, these criteria yielded a structurally and functionally diverse subset including CdeM, CdeC and SipL,^12,39,50,51^ CotE,^40,52^ CotA, CotB and CotJC1,^38,42,53^ CDIF27147_03838,^55^ CDIF27147_02043, and CDIF27147_02282. In addition, CDIF27147_01111, CDIF27147_00632, and CDIF27147_00446 ^54^ were repeatedly detected in the MS/MS data sets but did not meet all inclusion criteria and were included as negative controls. To confirm E-cadherin binding to candidate proteins, these 13 proteins were overexpressed in *E. coli* BL21­(DE3) pRIL and soluble and insoluble fractions were analyzed by far-Western blotting with human E-cadherin (Figures 4, S4–S6 and S8–S12). Results show that His-tagged proteins found at their expected molecular weights: CotE (∼83 kDa), CotA (∼37 kDa), CdeM (∼22 kDa), CotB (∼37 kDa), CDIF27147_00632 (∼86 kDa), CDIF27147_03838 (∼18 kDa), CDIF27147_01111 (∼42 kDa), CDIF27147_02043 (∼38 kDa), CdeC (∼45 kDa), SipL (∼61 kDa), CDIF27147_00446 (∼34 kDa), CotJC1 (∼21 kDa), and CDIF27147_02282 (∼22 kDa) (Figure 4). In 12 constructs, the recombinant protein was found in the insoluble fraction, common for overexpressed spore coat and exosporium proteins which form inclusion bodies in *E. coli* ^56–58^ (Figures 4, S4–S6 and S8–S12). However, CDIF27147_00446 (∼34 kDa) and CDIF27147_02282 (∼22 kDa) were detected in the soluble fraction (Figure 4K,M; Figure 3S K,M). Among the 13 recombinant candidate proteins tested, only four (CotE, CdeM, CDIF27147_02282, and CDIF27147_03838) were immunoreactive for E-cadherin antibodies (Figure 4).

**Figure 4:**
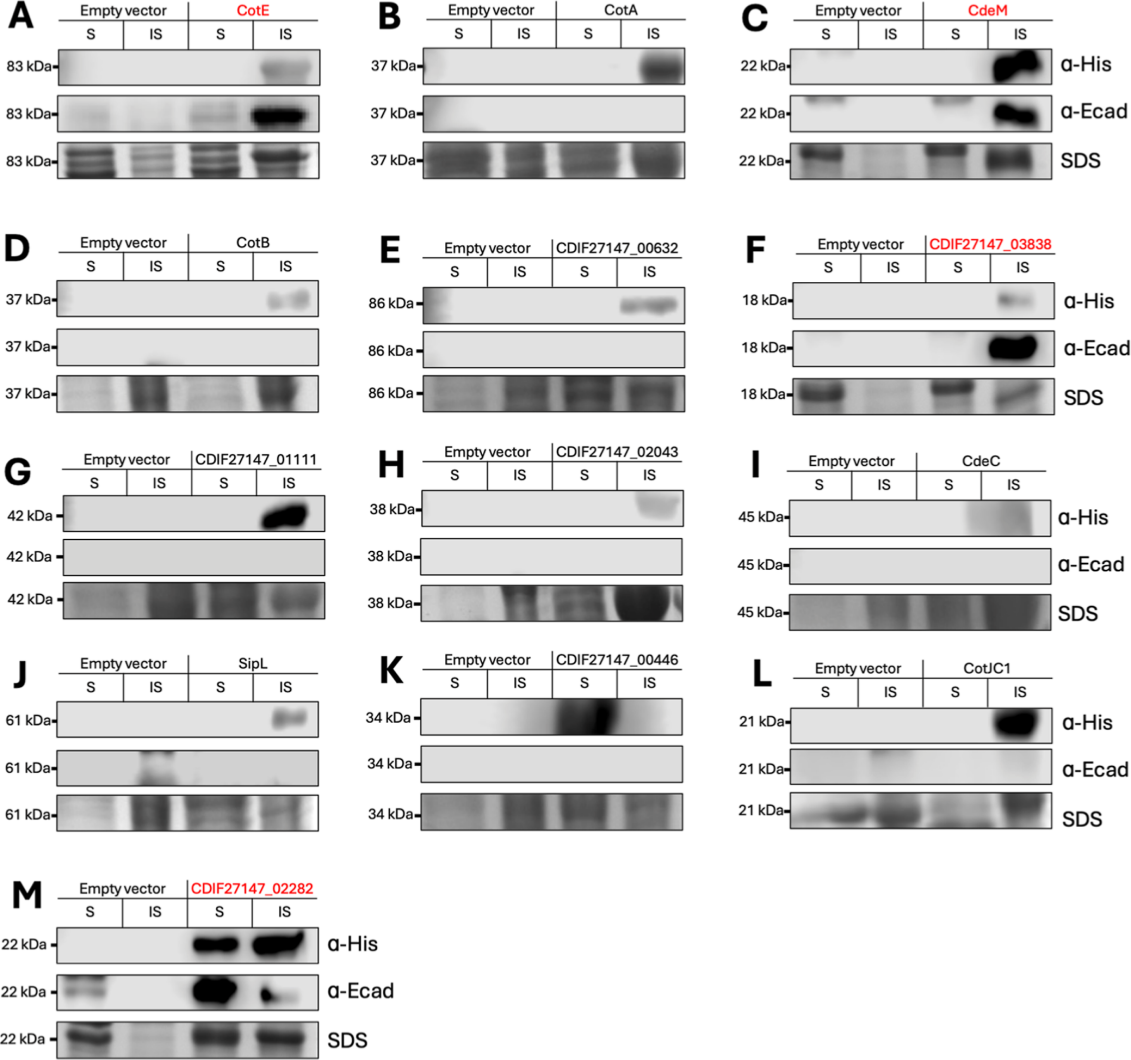
Detection of E-cadherin binding by candidate *C. difficile* R20291_CM196_ spore surface proteins expressed in *E. coli*. (A–M) Far-Western and anti-His immunoblot analysis of soluble (S) and insoluble (IS) fractions from *E. coli* expressing the indicated candidate receptor proteins or empty vector control. Lysates were separated by 12% SDS-PAGE, transferred to nitrocellulose, and probed sequentially with the (i) mouse anti-His antibody (α-His) to confirm expression, (ii) human recombinant E-cadherin followed by the rabbit anti-E-cadherin antibody (α-Ecad) to assess E-cadherin binding, and (iii) SDS-PAGE gel image for sample loading control. Far-Western blotting was performed by incubating membranes overnight with 1 μg/mL recombinant human E-cadherin, followed by the anti-E-cadherin antibody and HRP-conjugated secondary detection. Molecular weights are shown in kDa. Red protein names correspond to proteins that showed binding to E-cadherin in vitro.

### CotE, CdeM, CDIF27147_02282, and CDIF27147_03838 Are Conserved across *C. difficile* Five Classical Clades

To assess the evolutionary conservation of spore surface E-cadherin ligands and their functional relevance in pathogenesis, it is essential to understand the potential for universal targeting strategies across diverse clinical and ecological contexts. To do this, we performed amino acid sequence comparisons across the five classical *C. difficile* clades using a curated database of 250 whole-genome sequences from a published data set,^59^ comprising 50 representative isolates from each of the clades (Table S7). For each protein, the R20291 reference amino acid sequence (CDR20291_CP029423; Table S6) was used as a query, and protein conservation was assessed by tBLASTn searches against the isolate database, compiling percent pairwise identity and query coverage per clade (Table S8). CotE was found to be highly conserved across all clades, with an overall average identity of 99% to R20291 (Figure 5A). Identity was the highest in clades 1 (99.9%) and 2 (99.5%), slightly reduced in clades 3 (99.3%) and 4 (99.2%), and lowest in clade 5 (96.8%) (Figure 5A). CdeM displayed greater sequence diversity, with clade 4 showing the highest average identity (100%) (Figure 5B). Clade 1 followed (98%), while clades 2 (94.6%) and 3 (94.2%) grouped closely, and clade 5 was most divergent (92.7%) (Figure 5B). For CDIF27147_03838, identity to R20291 was the highest in clades 1 and 2 (99%), followed by clades 3 (98.4%) and 4 (98.4%), with clade 5 being the lowest at 96.7% (Figure 5C). CDIF27147_02282 similarly exhibited the greatest conservation in clade 2 (99.6%), then clade 1 (98.6%), clades 3 and 4 (98.2%), and the lowest in clade 5 (97%) (Figure 5D). In summary, all four E-cadherin ligands show high amino acid conservation (92.7–99.9%) across classical *C. difficile* clades, with differences being most evident for CdeM in clade 5.

**Figure 5:**
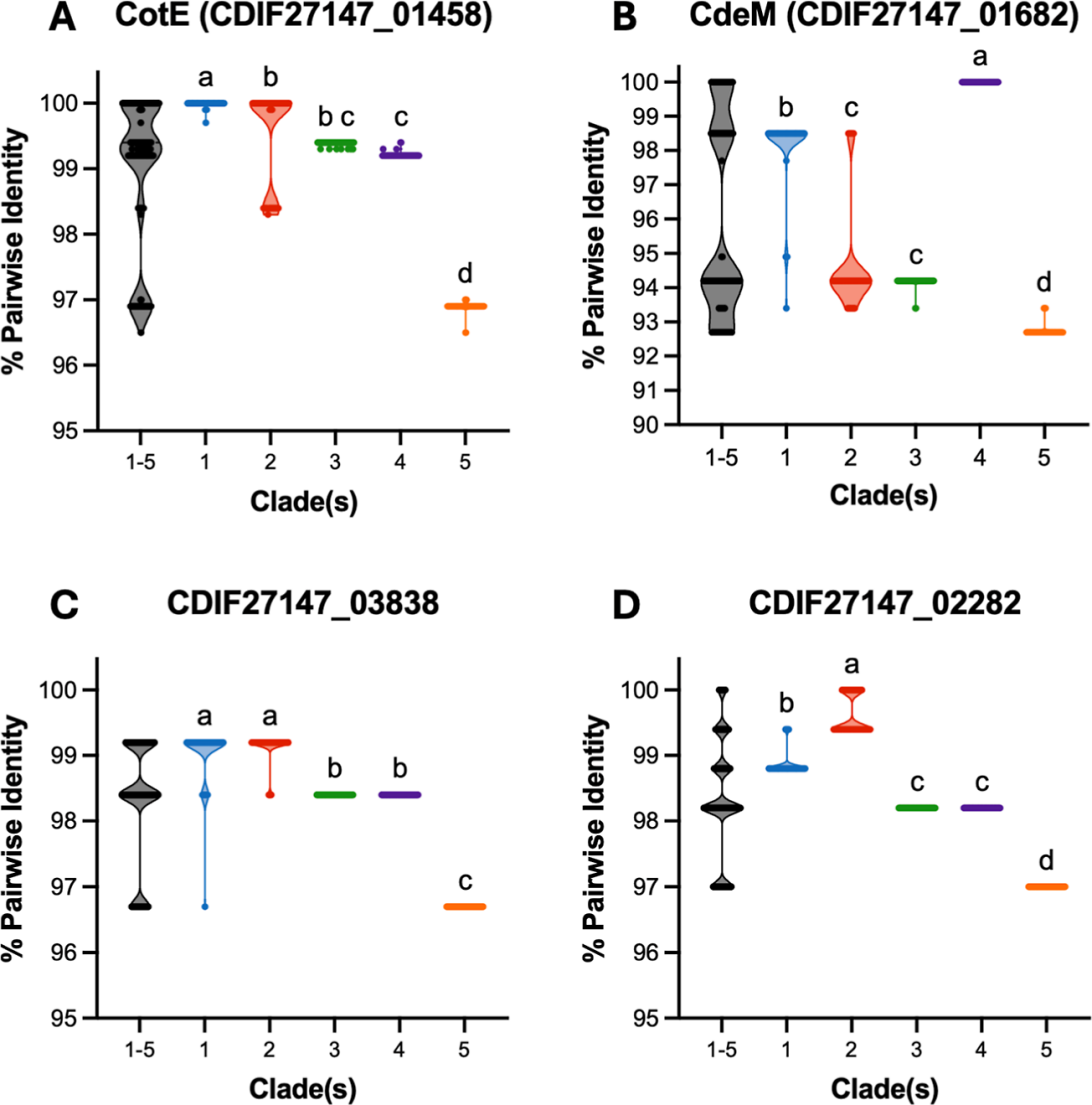
Conservation of candidate E-cadherin-binding proteins across *C. difficile* clades. (A–D) Violin plot distributions of pairwise amino acid identity for (A) CotE (CDIF27147_01458), (B) CdeM (CDIF27147_01682), (C) CDIF27147_03838, and (D) CDIF27147_02282 among representative genomes from each of the five major *C. difficile* clades. For each candidate protein, amino acid sequences were queried by tblastn against 250 genomes, with 50 strains per clade. The percent pairwise identity for each genome was extracted and plotted by clade to assess intra- and interclade sequence conservation profiles. Statistical significance was determined by ordinary one-way ANOVA, with Šídák’s multiple comparison test. Groups not sharing a letter are significantly different (*P* < 0.05) as determined by the multiple comparison test. Groups sharing one or more letters are not significantly different.

### *C. difficile* Spores Exhibit Conserved Binding to E-Cadherin across Five Classical Clades

To investigate how conserved E-cadherin binding is across *C. difficile* five classical clades, pull-down binding assays were conducted using spores purified from representative strains of each of the five classical clades. Binding affinity to E-cadherin was assessed by incubating purified *C. difficile* spores from each strain with recombinant human E-cadherin and performing a Western blot using an antibody against E-cadherin. SDS-PAGE of spore coat and exosporium extracts revealed distinct protein banding patterns among clade strains, suggesting compositional differences that may underlie variations in the E-cadherin binding affinity (Figure 6A–E). To ensure accurate comparisons, spore samples were quantified, validated for purity by phase-contrast microscopy, and equally loaded for Western blot analysis (Figure 6K). Representative strains were selected based on availability in our laboratory collection selected based on our recent work,^60^ with at least two strains included for each clade and three strains available for clade 5. Microscopy confirmed that all preparations consisted predominantly of pure, phase-bright spores with minimal contamination (Figure 6K).

**Figure 6:**
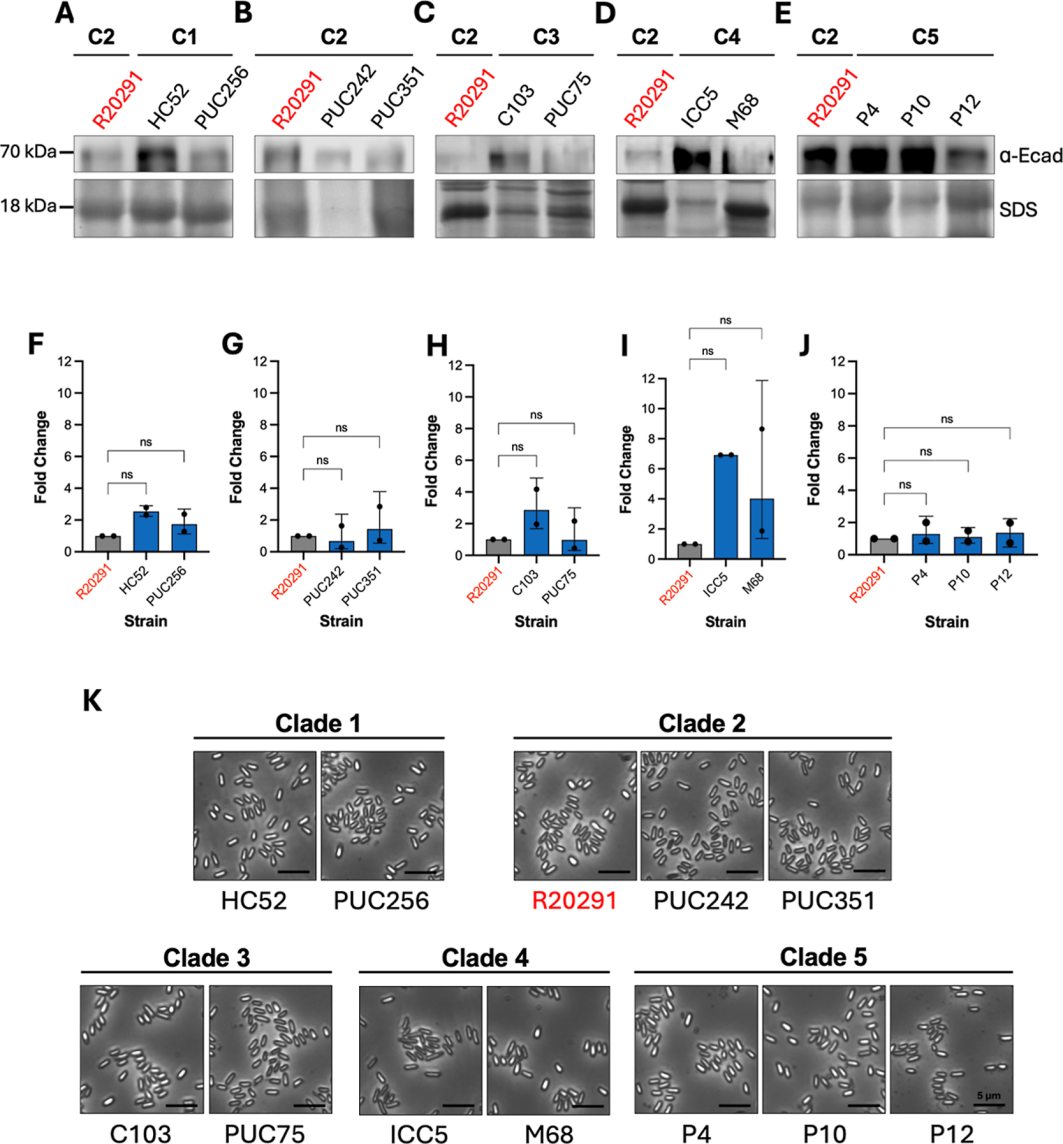
Pull-down analysis of E-cadherin binding by spores from diverse *C. difficile* Clade strains. (A–E) Equal numbers of *C. difficile* spores from strains of clades 1, 2, 3, 4, and 5 were incubated with 1 μg/mL recombinant human E-cadherin, and coat and exosporium extracts were resolved by SDS-PAGE and immunoblotted with the anti-E-cadherin antibody. (F–J) Quantification of E-cadherin binding to spore surface proteins for each strain, plotted as fold change relative to the R20291_CM196_ reference strain (Clade 2). Data represent mean ± SD from three independent experiments. Statistical significance was determined using Šídák’s multiple comparisons test (**** = *P* < 0.0001, ns = not significant). (K) Representative phase-contrast images of purified spores from each strain, illustrating sample purity prior to protein extraction and Far-Western analysis. Scale bar, 5 μm.

Quantitative analysis revealed that spores of all strains had E-cadherin binding, with R20291 (Clade 2) serving as the reference baseline (Figures 6A–J and S13–S15). Strains from clades 1, 2, 3, and 5 showed binding levels comparable to the control, with no significant differences among these groups when compared to the R20291 strain (Figures 6A–F and S13–S15). Although densitometric analysis showed that clade 4 strains exhibited a higher fold change in E-cadherin binding compared to R20291, this difference did not reach statistical significance (Figure 6I). Differences were observed in the ∼20 kDa loading control for most strains, reflecting variability in the surface layer protein abundance. Because an identical number of spores (1 × 10^8^ spores) were used to pull down E-cadherin, loading extracts from identical spore numbers in this experiment provides more accuracy than relying on a loading control. These results show that E-cadherin binding activity is conserved across representative strains of *C. difficile* five classical clades, consistent with the high amino acid conservation of E-cadherin ligand candidates.

### Peptide Microarray Identifies E-Cadherin Binding Motifs in Spore Ligands

To gain fine-details of the amino acid residues implicated in E-cadherin binding within these proteins, we performed conformational peptide microarrays using overlapping cyclic 9-mer and 13-mer peptides spanning key candidate proteins: CotE, CdeM, CDIF27147_02282, and CDIF27147_03838. In addition, in prior works, we observed that E-cadherin comes in close proximity to the hair-like projections of *C. difficile* spores which are made by BclA2 and BclA3.^32,36^ However, since these proteins are trypsin-digestion resistant and undetectable through MS/MS, we thought to include both C-terminal domains of BclA2 and BclA3 to test whether they were implicated in E-cadherin binding. As a control for nonspecific secondary antibody binding, rabbit anti-GST DyLight 680 was applied to a peptide microarray containing 2972 cyclic peptides prior to any primary antibody incubation, resulting in no detectable interactions with the secondary antibody (Figure S16). Because no E-cadherin binding spore proteins had been defined prior to this work, established peptide-level positive controls were not available. Quantitative analyses of E-cadherin binding intensities to individual peptides are shown for 1 μg/mL E-cadherin and 10 μg/mL E-cadherin (Figure 7B,C,E,F, respectively). Each bar represents the mean fluorescence intensity of duplicate peptide spots after background subtraction and outlier filtering (Figure 7B,C,E,F). Together, these plots show how E-cadherin binding varies across peptide sequences and increases in detectability when the ligand concentration is raised (Figure 7B,C,E,F). At an E-cadherin concentration of 1 μg/mL, a ligand concentration similar to physiological conditions, E-cadherin showed a high binding affinity (>10,000 fluorescence intensity [a.u.]) to two motifs within CotE; CotE_4, which spans 9 amino acids at positions 273–281 within a peroxiredoxin domain (1–377) with the sequence KKHTKNTNK and CotE_7, which spans 12 amino acids at positions 339–350 with the sequence KNTKKIAMKTLK (Figure 7A–C). Additionally, moderate binding affinity (>1000 and <10,000 fluorescence intensity [a.u.]) within a peroxiredoxin domain (1–377) of CotE was observed for motifs CotE_1 spanning 18 amino acids at positions 44–61 with sequence PVCTTEFLCFAKYYDEFK, CotE_2 spanning 20 amino acids at positions 200–219 with sequence YSCMDWYLCFVPDNYNDEEV, and CotE_6 spanning 16 amino acids at positions 315–330 with sequence YNKEDSSYEDFYKHNY (Figure 7A–C). Nine other regions within CotE, including three regions in the Chitinase domain, exhibited detectable binding above background but below 1000 fluorescence intensity [a.u.], indicating lower affinity or nonspecific binding (Figure 7A–C). CdeM, CDIF27147_02282, and CDIF27147_03838 only resulted in regions of moderate interaction (>1000 fluorescence intensity [a.u.]) with E-cadherin (Figure 7A–C). In CdeM, these regions include CdeM_1 motifs, spanning 14 amino acids from positions 4–17 with the sequence KKCYSEDWYERGES and CdeM_5 motifs at 17 amino acids long located at positions 96–112 with the sequence LWEEAEKYWDEYSKYNY (Figure 7A–C). For CDIF27147_02282, the 2282_3 motif is 20 amino acids in length, positioned at residues 71–90, with the sequence TDKFSLTFFDEDFRKELSYC (Figure 7A–C). Lastly, in CDIF27147_03838, the 3838_1 motif, comprising 10 amino acids at positions 1–10 with the sequence MYDDYKYNKC, 3838_2 motif spans 18 amino acids from residues 11–28, with sequence NKCYNDYEEMTHVHEYSE, and 3838_4 motif, 13 amino acids in length at positions 65–76, with sequence NDNVDFLDHFHK (Figure 7A–C). For the C-terminal domains of BclA2 and BclA3, the peptide microarray did not yield significant interactions with E-cadherin (Figure 7A–C). Together, these results identify multiple discrete E-cadherin binding motifs within CotE and reveal several regions of moderate affinity in CdeM, CDIF27147_02282, and CDIF27147_03838, while suggesting that the C-terminal domains of BclA2 and BclA3 do not contribute to direct E-cadherin interactions under these conditions.

**Figure 7:**
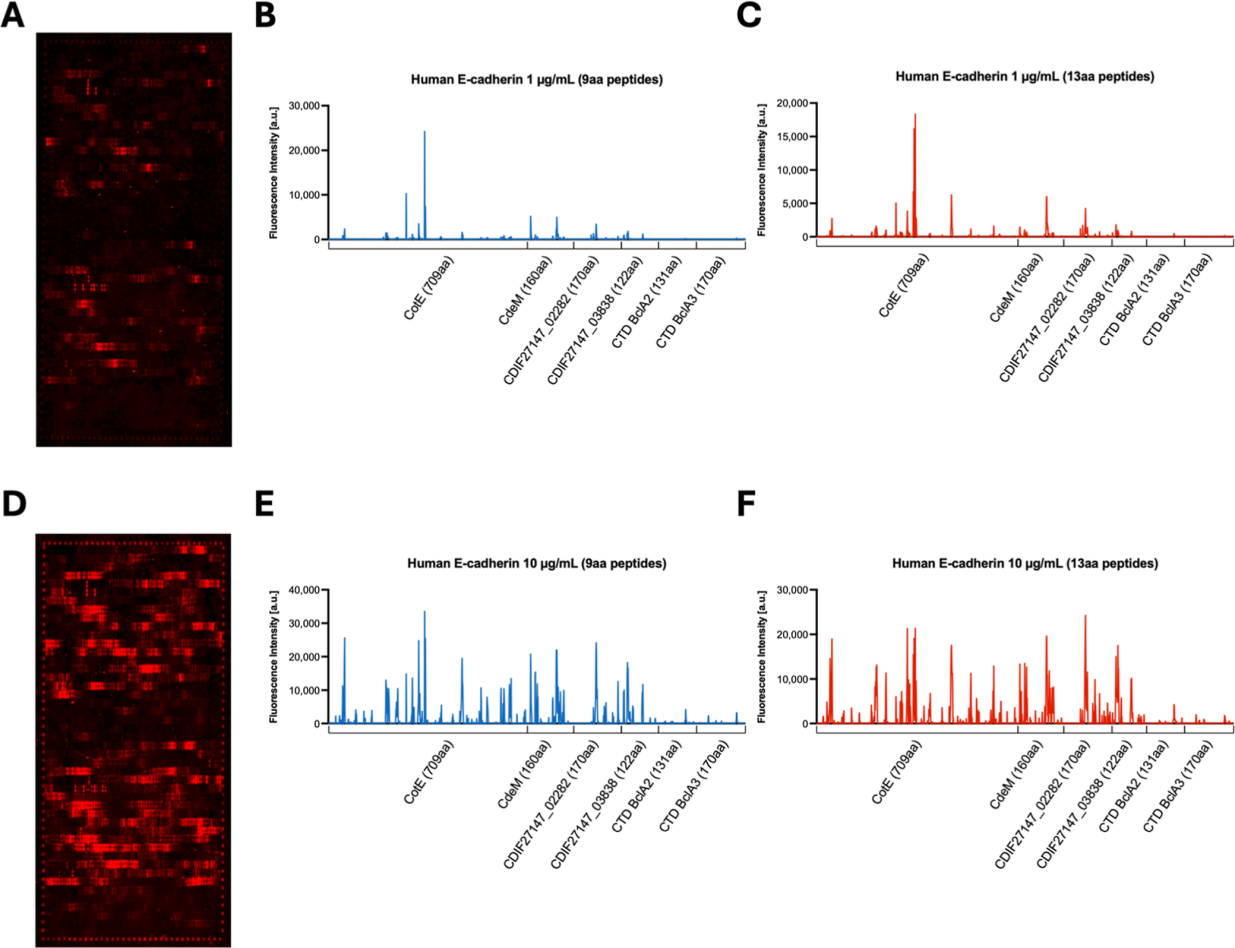
Identification of E-cadherin interaction motifs by a conformational peptide microarray. (A,D) Representative fluorescence images from conformational peptide microarrays incubated with recombinant human E-cadherin at (A) 1 μg/mL and (D) 10 μg/mL. Arrays contained overlapping cyclic 9-mer and 13-mer peptides covering sequences from CotE, CdeM, CDIF27147_02282, CDIF27147_03838, and the C-terminal domains of BclA2 and BclA3, with duplicate peptide spots and HA control frames. (B,C,E,F) Quantification of E-cadherin binding to individual peptides. Bar graphs represent fluorescence intensities (arbitrary units, a.u.) for E-cadherin 1 μg/mL (9aa in B, 13aa in C) and for 10 μg/mL (9aa in E, 13aa in F), grouped by a protein source. Data were analyzed after background correction and outlier filtering, with only peptide spots below the 40% deviation threshold included.

To detect weaker binding motifs and additive electrostatic and hydrophobic interactions, we increased E-cadherin concentration to 10 μg/mL. At this concentration, we observed 33 regions with a binding affinity above background within CotE (Figure 7D–F). Notably, ten of these regions showed a high fluorescence intensity (>10,000 [a.u.]), with five corresponding to motifs CotE_1, CotE_2, CotE_4, CotE_6, and CotE_7 (Figure 7D–F). For CdeM, there were 12 regions which exhibited a fluorescence above background, and five exceeded >10,000 [a.u.] including those of motifs CdeM_1 and Cdem_5 (Figure 7D–F). In CDIF27147_02282, seven regions showed a signal above background, with two >10,000 fluorescence intensity [a.u.] including motif 2282_1 (Figure 7D–F). Lastly within CDIF27147_03838, six regions displayed fluorescence intensity above background, three of which had >10,000 fluorescence intensity [a.u.] corresponding to motifs 3838_1, 3838_2 and 3838_4 (Figure 7D–F). In the case of C-terminal domains of BclA2 and BclA3, no significant signal with E-cadherin was observed (Figure 7D–F). Increasing E-cadherin concentration to 10 μg/mL uncovered numerous additional binding motifs, indicating that higher ligand availability enhances detection of both strong and weaker protein–ligand interactions, while confirming that binding capacity is distributed across multiple regions within CotE, CdeM, CDIF27147_02282, and CDIF27147_03838 (Figure 7D–F). However, neither BclA2 nor BclA3 C-terminal peptides showed fluorescence above the threshold for specific interactions, indicating that these regions lack detectable E-cadherin binding motifs under our assay conditions. This finding is consistent with prior ELISA assays using *bclA1*, *bclA2*, and *bclA3* mutants in strain 630, in which E-cadherin binding to spores was maintained or even increased, supporting the conclusion that the C-terminal domains of BclA2 and BclA3 do not function as direct E-cadherin ligands in these conditions.^32^ Collectively, these results demonstrate that E-cadherin binding to CotE, CdeM, CDIF27147_02282, and CDIF27147_03838 occurs through multiple, discrete motifs, with binding affinities that increase as a ligand concentration is raised, while C-terminal domains of BclA2 and BclA3 consistently show no significant interaction; this highlights extensive multivalent recognition by select spore proteins as the basis for E-cadherin binding.

### Surface Accessibility, Acidic, and Hydrophilic Features Predominate among E-Cadherin Binding Motifs on *C. difficile* Spore Ligands

To define the physicochemical properties and structural contexts of E-cadherin binding motifs identified on spore surface ligands, we combined peptide microarray data with protein structure prediction. For this, we used AlphaFold3 models to understand the three-dimensional structure and accessibility of these binding motifs within the candidate ligands (Figure 8E–H).^61^ For all four proteins (CotE, CdeM, CDIF27147_02282, and CDIF27147_03838), binding motifs identified by the peptide microarray with fluorescence intensities above 1000 [a.u.] using 1 μg/mL E-cadherin were highlighted in the prediction model and subjected to site-specific physicochemical characterization (Figure 8A–D). Within CotE, five binding motifs were mapped to distinct structural domains (Figure 8E). CotE_1 (18aa; position 44–61) is within an exposed α-helix, exhibiting a strongly acidic character (pI ∼ 4.2, net charge −5.8) and transitioning from hydrophobic to hydrophilic surface features (Figure 8E). Motif CotE_2 (20aa; position 200–219) is found at the interface of an α-helix and β-strand, distinguished by mixed hydrophobicity and a substantial negative surface charge (pI ∼ 3.4, net charge −6.0) (Figure 8E). This motif is similarly accessible, presenting both a flexible secondary structure and strong hydrophilicity (Figure 8E). A CotE_4 motif (9aa; position 273–281) is within a coil region, fully exposed and relatively neutral in charge, maintaining a strongly hydrophilic profile (Figure 8E). Motif CotE_6 (16aa; position 315–330) is an α-helical domain that is highly surface-accessible, moderately acidic (pI ∼ 6.8, net charge +2.8), and shifts from hydrophobic interior to a hydrophilic exterior (Figure 8E). A CotE_7 motif (12 aa; position 339–350) is part of an α-helix at the peroxiredoxin domain and displays amphipathic properties with a moderately polar charge distribution (Figure 8E). The AlphaFold 3 model of CotE is predicted with a generally high overall confidence, with most of the structured domains showing pLDDT values in the confident to a very high range (≥70–90). Within this context, CotE_1, CotE_6, and CotE_7 lie predominantly in these high-confidence regions, whereas CotE_2 and CotE_4 overlap segments where the pLDDT drops into lower ranges, indicating locally reduced confidence compared with the rest of the model. In CdeM, the motif CdeM_1 (14aa; position 4–17) is characterized by a strong positive surface charge (pI ∼ 10.5, net charge +4.1), moderate hydrophobicity, and an exposed α-helical geometry (Figure 8F). Alternatively, the CdeM_5 motif (17aa; position 96–112) is a highly hydrophilic, negatively charged region (pI ∼ 4.6, net charge −2.8) that lies within an elongated surface helix, presenting a strongly polar interface (Figure 8F). The predicted model of CdeM from AlphaFold3 shows an overall high structural confidence, with most of the coiled-coil core predicted in the pLDDT range of ≥70 and frequently ≥90. Within this context, CdeM_5 falls entirely in a high-confidence helical segment, whereas CdeM_1 overlaps a lower confidence region (pLDDT ∼ 50–70), indicating that its precise local conformation is less certain even though the overall helical scaffold is well supported. In the case of CDIF27147_02282, motif 2282_3 (20aa; position at 71–90) is part of an α-helix and β-strand, marked by a strong acidic residue composition (pI ∼ 4.4, net charge −4.3) and prominent hydrophilicity (Figure 8G). For CDIF27147_02282, the AlphaFold 3 model shows uniformly very high confidence (pLDDT mostly >90) across the β-sandwich core. The E-cadherin-binding motif 2282_3 lies within this same high-confidence region, indicating that its position and local geometry are predicted with strong reliability. Lastly, for CDIF27147_03838, motif 3838_1 (10aa; position 1–10) is part of a coil region that is accessible on the protein surface (Figure 8H). This motif exhibits a strong hydrophilic character and carries a modest acidic charge (pI approximately 5.7, net charge −0.8) (Figure 8H). In contrast, 3838_2 (18aa; position 11–28) resides in a partly disordered coil interspersed with β-strand secondary structure elements (Figure 8H) that presents a more strongly acidic and hydrophilic surface, with a pI near 4.6 and net negative charge near −4.6, creating an extensive negatively charged patch (Figure 8H). Additionally, 3838_4 motif (13aa; positions 65–76) is part of a coil region but displays a mixed hydropathy profile, with a transition from hydrophilic to more neutral residues and a moderate negative charge (pI ∼ 5.2, net charge −2.5) (Figure 8H). Structurally, it comprises coil and β-strand elements and possesses significant exposure (Figure 8H). Lastly, the AlphaFold 3 model of this protein has a well-defined β-sandwich core predicted with high confidence (pLDDT ≥ 70–90), while the N-terminal extension is lower confidence and likely more flexible. Within the model, the three E-cadherin-binding motifs map to distinct regions: 3838_2 lies in a high-confidence β-strand within the structured core, 3838_4 occupies an adjacent region of similarly high confidence, and 3838_1 extends into the lower confidence N-terminal tail, indicating that two motifs are in reliably modeled structural elements whereas the tail-associated motif resides in a more conformationally uncertain segment. CotE_4 and CotE_7 motifs, which were the motifs with the highest E-cadherin-binding, have a strong positive surface charge. These E-cadherin binding motifs are surface-accessible, moderately sized (9–20aa), predominantly hydrophilic or acidic in character, and commonly within flexible α-helical or coil structures. In terms of spore localization, CotE has been detected in the outer spore coat that underlines the exosporium and is partially removed by sonication, consistent with a spore surface-exposed protein.^16,40,42^ CdeM is a cysteine-rich exosporium morphogenetic protein that localizes to the exosporium layer and is accessible to antibodies on intact spores, confirming its surface exposure.^12^ CDIF27147_02282 and CDIF27147_03838 were identified in exosporium/coat-enriched surface extracts, suggesting that they are surface exposed. However, for CDIF27147_02282 and CDIF27147_03838 whether they are surface exposed remains unclear, and thus their E-cadherin-binding motifs are interpreted as surface-accessible to E-cadherin, not in the context of the exosporium surface.

**Figure 8:**
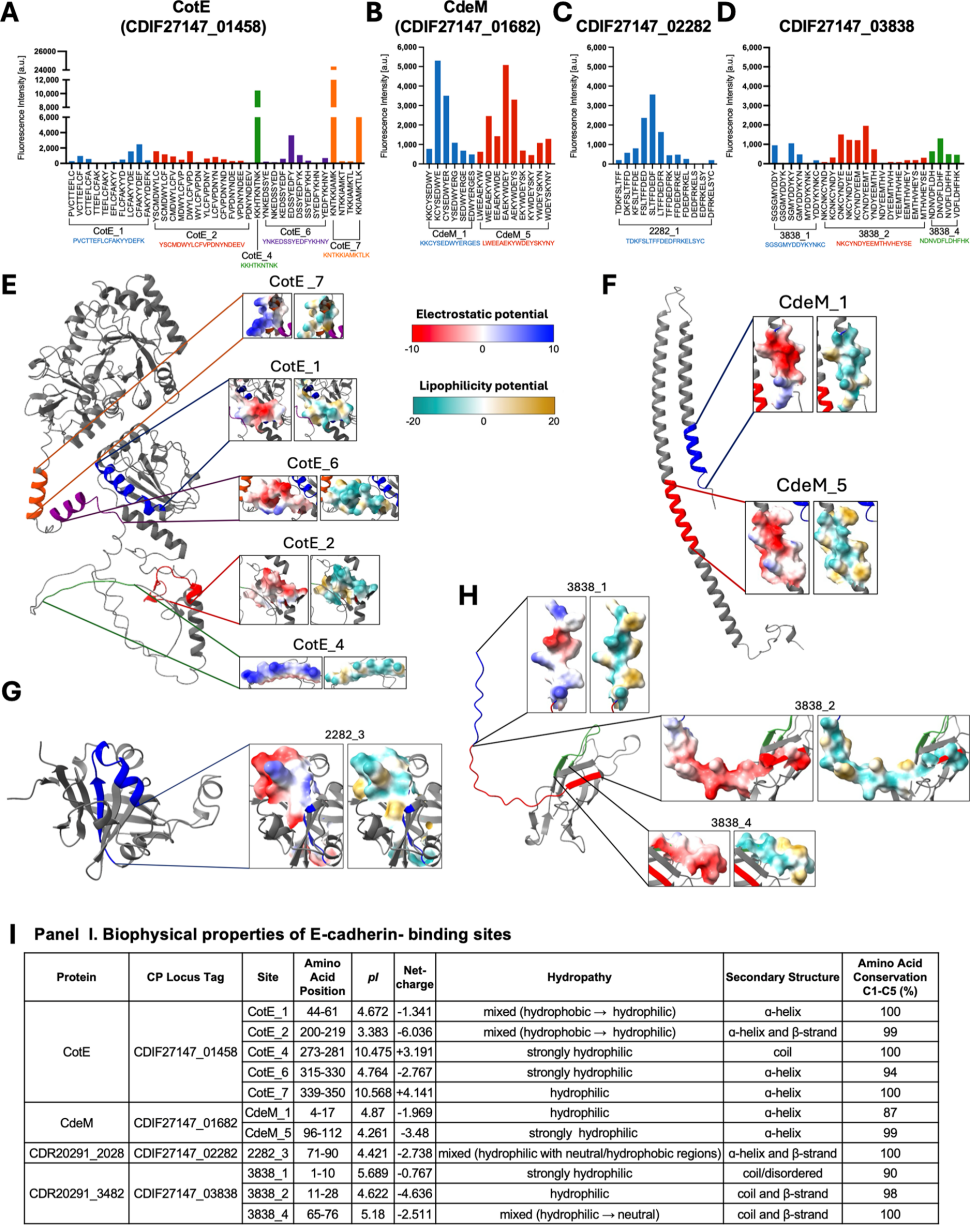
Biophysical and structural mapping of E-cadherin binding motifs in *C. difficile* spore/exosporium proteins. (A–D) Plots of peptide microarray fluorescence intensities overlaid onto linear protein sequences for (A) CotE, (B) CdeM, (C) CDIF27147_02282, and (D) CDIF27147_03838. Regions corresponding to high E-cadherin binding are highlighted and color-coded for structural analysis. (E–H) AlphaFold-predicted protein structures visualized with ChimeraX for (E) CotE, (F) CdeM, (G) CDIF27147_02282, and (H) CDIF27147_03838, with mapped E-cadherin binding motifs highlighted. Insets show zoomed views of physicochemical surface features using ChimeraX: electrostatic potential values in units of kcal/(mol) using Coulombic surface coloring (−10 red, 0 white, 10 blue) and molecular lipophilicity potential based on pyMLP (dark cyan (most hydrophilic), white to dark goldenrod (most lipophilic)) of each binding site. (I) Summary of the predicted properties of major E-cadherin binding regions, including an isoelectric point (*pI*), net charge, hydropathy classification, and secondary structure elements, as determined by Prot pi, ProtScale, and PSIPRED analyses.

Next, we assessed amino acid conservation for each motif across the five classical *C. difficile* clades using a curated database of 250 whole-genome sequences from a published data set (Figure S17).^59^ Binding motif sequences were extracted and pairwise percent identity was calculated for each motif, allowing us to compare conservation patterns (Figure S17). This analysis revealed that motifs such as CotE_1, CotE_2, CotE_4, CotE_7, 2282_3, and 3838_4 exceeded 95% in a pairwise identity across all five clades, indication of high conservation of these sequences (Figure S17A–C,E,H,K). Among the other binding motifs evaluated, CotE_6 displayed variable conservation across clades, with pairwise identity ranging from ∼81% in clade 5 to 94–100% in clades 1–4 (Figure S17D). The CdeM_1 motif showed an even greater divergence, being highly conserved on average in clades 1 and 4 (98–100%), and dropping to 79% in clades 2, 3, and 5 (Figure S17F). Motif CdeM_5 remained well conserved in most clades but showed reduced identity (∼94%) specifically in clade 5 (Figure S17G). For the 3838_1 motif, overall conservation was moderate, with pairwise identities of 90% across all five clades (Figure S17I). The 3838_2 motif was generally well-conserved (98–99%) in clades 1–4 but demonstrated divergence in clade 5 with an identity of 89% (Figure S17J). Together, these observations underscore that while several E-cadherin binding motifs are highly conserved, motifs such as CotE_6, CdeM_1, CdeM_5, 3838_1, and 3838_2 exhibit substantial clade-dependent diversity, which may influence E-cadherin binding properties among *C. difficile* strains (Figure S17).

### Synthetic Peptides Did Not Inhibit E-Cadherin Binding to *C. difficile* Spores

To determine if synthetic peptides of these E-cadherin binding motifs could inhibit binding to *C. difficile* spores, we synthesized synthetic peptides designed based on high-affinity E-cadherin binding motifs identified from a peptide microarray, with sequences corresponding to regions of candidate spore surface proteins that exhibited strong binding (fluorescence intensity >1000 au). Peptides ranged from 9 to 20 amino acids in length, tailored to match the mapped motif sequences. Pull-down assays were performed by incubating recombinant E-cadherin with synthetic peptides at concentrations ranging from 1 to 15 μM prior to mixing with R20291 spores. Peptide concentrations ranging from 1 μM (a subsaturating dose, less than 10-fold above the measured *K*_d_ of 110 nM) to 15 μM (well above *K*_d_, ensuring saturating conditions) were selected to enable detection of competitive inhibition across the relevant range. A preincubation step was done to allow peptides to interact with E-cadherin prior to incubation with *C. difficile* spores. As a negative control, a scrambled peptide was created by randomly rearranging the amino acid sequence of CotE_4, the motif with the strongest affinity to E-cadherin, while retaining its length and composition. This control was chosen to assess whether inhibitory activity requires the specific arrangement of residues rather than general properties like length, amino acid composition, charge, or hydrophobicity.^62^ Across the tested concentrations (1 to 15 μM), synthetic peptide CotE_1 (sp-CotE_1) led to a dose-dependent 3.5-fold increase in E-cadherin binding (Figures 9F,G; S18A,B and S20A). In contrast, sp-CotE_2 and the scramble peptide control showed no reduction in E-cadherin binding (Figures 9F,G; S18A,B and S20A). Likewise, sp-CotE_4 and sp-CotE_7 failed to inhibit E-cadherin binding and had no reduction relative to control strain R20291 (Figures 9B,H; S18C,D and S20B). The final CotE peptide, CotE_6, similarly showed no detectable effect on E-cadherin binding relative to the control (Figures 9C,I; S18E,F and S20C). Additionally, CdeM_1 and CdeM_5 did not result in significant changes in E-cadherin binding to spores, as seen by densitometry values relative to the control (Figures 9D,J; S19A,B and S20D). Similarly, sp-3838_1, sp-3838_2, sp-3838_4, and sp-2028_3 showed no inhibition of E-cadherin binding compared to peptide control (Figures 9E,F,K,L; S19C–F and S20E,F). Furthermore, combining all CotE-derived peptides at 1 μM or 15 μM of each also failed to inhibit E-cadherin binding (Figure S20G). Altogether, these results show that synthetic peptides corresponding to E-cadherin binding motifs are not sufficient to competitively inhibit spore binding to E-cadherin under these conditions.

**Figure 9:**
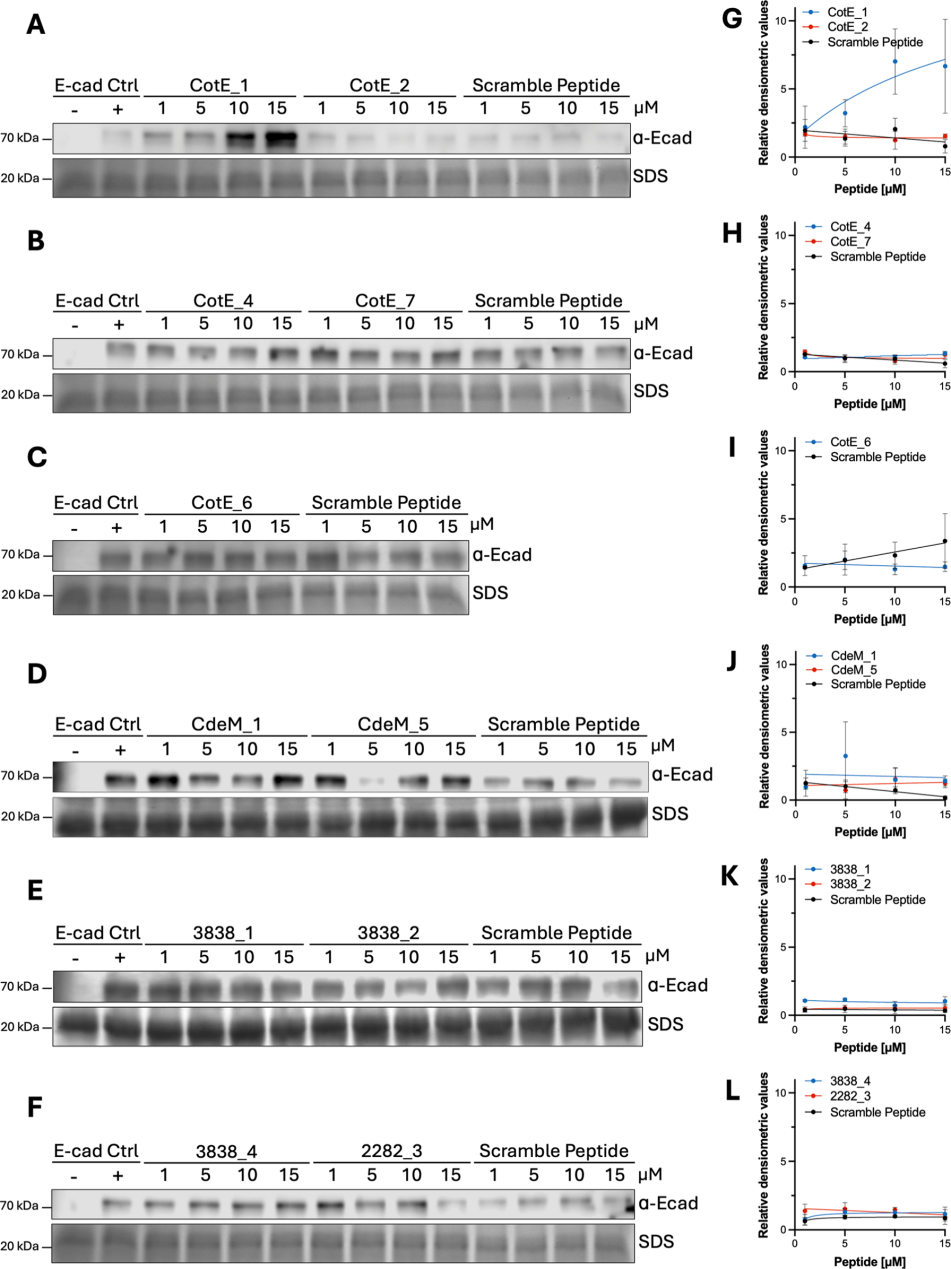
Effect of synthetic peptides in *C. difficile* R20291_CM196_ spore binding to E-cadherin based on pull-down assays. (A–F) Far-Western blots showing the effect of increasing concentrations (1, 5, 10, and 15 μM) of synthetic peptides, corresponding to putative E-cadherin interaction sites in CotE, CdeM, CDIF27147_03838, and CDIF27147_02282, on E-cadherin binding to R20291_CM196_ spores. E-cadherin controls correspond to R20291_CM196_ spores incubated in the absence or presence of E-cadherin without added synthetic peptides. Spores were incubated with E-cadherin premixed with each peptide, and bound E-cadherin detected with an anti-E-cadherin antibody. SDS-PAGE gels shown beneath each blot serve as loading controls. (G–L) Quantification of E-cadherin binding by densitometric analysis, shown as relative densiometric values versus peptide inhibitor concentration for each peptide and control scramble peptide, and E-cadherin positive and negative control. Data represent mean ± SD from two independent assays.

## Discussions

Recurrent *C. difficile* infections remain a major challenge in clinical management, mainly driven by the persistence of metabolically dormant spores within the host and healthcare environment.^8,16^ Recent work demonstrates that direct interactions between spore surface proteins and host cell proteins, including E-cadherin, vitronectin, and fibronectin, play a central role in spore adherence or internalization to intestinal epithelial cells, phenotypes that are linked to disease recurrence.^32,35^ Previous studies have also shown that the disruption of cell–cell junctions by TcdA and TcdB toxins increases accessibility of E-cadherin which also increases spore adherence to intestinal epithelial cells.^32^ The capacity of *C. difficile* to interact with E-cadherin is not unique to this bacterium, other organisms like *Listeria monocytogenes*, *Campylobacter jejuni*, and *Streptococcus pneumoniae* have been shown to utilize E-cadherin to internalize into host cells.^63–65^ In this study, we expand our understanding of how *C. difficile* spores interact with E-cadherin by identifying four spore surface proteins and their motifs that act as E-cadherin ligands.

A major contribution of this work is the identification of CotE,^40–42^ CdeM,^12^ and two previously uncharacterized exosporium or coat proteins, CDIF27147_02282 and CDIF27147_03838, as E-cadherin spore surface ligands. CotE is a bifunctional protein implicated in mucin binding and degradation, as well as colonization in vivo.^40^ Loss of *cotE* significantly reduces virulence and colonization efficiency in animal models, emphasizing its central role in host–pathogen interactions.^40^ Similarly, CdeM is involved in exosporium assembly, and its disruption leads to a diminished spore adherence to epithelial cells and compromised colonization in healthy host mucosa.^12^ Our data expand these functions, demonstrating that both, CotE and CdeM, possess multiple E-cadherin binding motifs, identified by peptide microarrays, which cluster predominantly within accessible α-helical and coil regions. Additionally, CotE possesses a C-terminal β-barrel Chitinase fold enriched in E-cadherin binding sites. Our findings also reveal that neither BclA2 nor BclA3 C-terminal domains have binding motifs for E-cadherin, an observation supported by previous studies showing that *bclA2* and *bclA3* mutants display wild-type or even enhanced E-cadherin binding.^32^ Altogether, our work has identified E-cadherin binding as a novel role for CotE and CdeM, however, future works will be essential to understand the mechanism used by these proteins and their motifs for spore–host interactions to contribute to colonization and disease outcomes.

In addition, this work extends the repertoire of candidate E-cadherin ligands by identifying two previously uncharacterized proteins, CDIF27147_02282 and CDIF27147_03838, whose expression is regulated by the sporulation-specific sigma factors σ^E^ and σ^K^. CDIF27147_02282 has a predicted flavin reductase domain identified using NCBI’s Conserved Domain Database (CDD) search,^66^ with an identified E-cadherin binding motif localized to an exposed α-helix within the predicted functional domain. CDIF27147_03838, a YmaF family homologue, which has previously been implicated in spore germination in *Bacillus* species,^55^ has three E-cadherin binding motifs mapped to the coil and α-helical region upstream of the predicted YmaF domain, suggesting the presence of two functional domains within this protein. Together, these findings identify CDIF27147_02282 and CDIF27147_03838 as novel E-cadherin ligands in *C. difficile* *.* However, outstanding questions remain regarding their abundance, spatial localization, and precise functional roles in spore adherence in vivo. Future studies employing targeted mutagenesis, and phenotypic analysis will be critical to confirm these proteins’ contributions to host–spore interactions and pathogenesis.

A second major conclusion is that E-cadherin binds to *C. difficile* spores with a predicted nanomolar affinity of *K*_d_ = 109.9 nM. In the context of protein–protein interactions, the strength of the binding affinity is generally defined by the equilibrium dissociation constant (*K*_d_) with moderate–affinity interactions defined as being in the low to midnanomolar range (approximately 10–500 nM), which are recognized as physiologically meaningful.^46^ In comparison, the binding affinity of E-cadherin to spores is considerably higher than that of *L. monocytogenes* InlA-E-cadherin complex (*K*_d_ = 8 μM), which is essential for host cell internalization,^67^ underscoring the potential biological importance of E-cadherin binding in the context of *C. difficile* spore adherence. The measured affinity is also comparable to that of other host glycoproteins such as fibronectin (*K*_d_ = 20.8 nM) and vitronectin (*K*_d_ = 106.5 nM),^11^ interactions that have been shown to enhance spore adherence to intestinal epithelial cells in a concentration-dependent manner. Altogether, these results advance our understanding of E-cadherin-spore interactions and defines it as a moderate-strength binding affinity, which contributes to spore adherence.

A third major conclusion is that E-cadherin binds heterogeneously to *C. difficile* spores, binding only to a subpopulation of spores. This observed tropism is consistent with previous observations,^32^ and suggest variability of ligands at the spore surface. The formation of two distinct exosporium morphotypes in *C. difficile*, thin exosporium and thick exosporium with a distinctive electrodense bumps,^13–15^ contributes to the observed heterogeneity in E-cadherin binding. Immunogold labeling showed that E-cadherin preferentially binds to spores with a thick exosporium.^32^ This apparent preference to spores with a thick exosporium would likely explain why E-cadherin binds to a subset of spores. Prior works suggest that there may be differences in expression levels and distribution of coat and exosporium in thin and thick exosporium spores.^13^ This variability between morphotypes suggests that the abundance of E-cadherin spore ligands identified in this work (e.g., CotE, CdeM, CDIF27147_02282, and CDIF27147_03838) differs and may be linked to the exosporium type. An attractive approach to directly test this would be fluorescence-activated sorting of spores based on E-cadherin binding, followed by a comparison of ligand abundance between E-cadherin bound and nonbound populations. However, this strategy is currently limited by the lack of validated antibodies for all candidate ligands (particularly CDIF27147_02282 and CDIF27147_03838) and by technical challenges associated with isolating sufficient quantities of sorted spores for downstream protein analysis. As an alternative, future directions to pursue these questions could benefit from utilizing translational fusions of these E-cadherin-ligand candidates to address how these candidate ligands are expressed and distributed on the surface and whether their abundance correlates with E-cadherin binding is warranted.

Another conclusion of this work is that *C. difficile* clade spores bind equally to E-cadherin, suggesting that epithelial adherence is a conserved property among classical clades of clinical relevance. The classical clades represent genetically distinct lineages that vary in epidemiology, host association, and virulence. Therefore, analyzing amino acid conservation of key spore ligands across these clades is critical to understanding whether mechanisms of host interactions, such as E-cadherin binding, are conserved and relevant to pathogenesis. Clade 1 is the most prevalent and found globally, commonly implicated in both community- and healthcare-associated infections,^68^ while clade 2 includes epidemic ribotype 027 (RT027) strains associated primarily with hospital outbreaks.^69^ Clade 3, while less common, harbors strains that phenotypically resemble clade 2.^70^ Clade 4 strains, which often exhibit antibiotic resistance and are connected to outbreaks in Asia, North America, and Europe.^71^ Lastly, clade 5 strains include toxin-producing strains that are prevalent in production animals.^72^ The ligands identified in our study, CotE, CdeM, CDIF27147_02282, and CDIF27147_03838, and the E-cadherin binding motifs within these proteins are well conserved across the five classical *C. difficile* clades. Amino acid conservation results for CotE and CdeM are consistent with previous genomic analyses and essential roles in the spore structure, integrity, and host interactions.^12,39,40^ Amino acid conservation in E-cadherin ligands supports the conclusion that E-cadherin spore adherence capability is broadly maintained across *C. difficile* clades. However, motif-level analysis revealed that while several binding motifs, including CotE_1, CotE_2, CotE_4, CotE_7, 2282_3, and 3838_4, are highly conserved (>95% pairwise identity) in all five classical clades, other motifs, such as CotE_6, CdeM_1, CdeM_5, 3838_1, and 3838_2, show substantial clade-dependent diversity. These findings indicate that although overall E-cadherin binding capacity is conserved, the specific ligand–motif interactions may vary among clades. Altogether, these results suggest that structural elements needed for interactions with E-cadherin are maintained, even when minor sequence differences exist. A limitation of this work is that direct immunofluorescence was not conducted for all studied clade strains; however, prior studies have documented the production of both thick and thin exosporium morphotypes across *C. difficile* clades,^14^ suggesting that spores from different clades share key structural features. This morphological similarity, together with the observed amino acid conservation, supports the expectation of comparable tropism and heterogeneous E-cadherin binding across strains from other clades. Nevertheless, future work including more isolates will be necessary to understand how well conserved E-cadherin binding is in other *C. difficile* strains.

A noteworthy observation is that synthetic peptides did not reduce *C. difficile* spore binding to E-cadherin by competitive inhibition. Unlike previous studies that demonstrated inhibition of *C. difficile* toxins or membrane interactions using short peptides,^73,74^ our approach specifically targeted E-cadherin aiming to competitively block interactions with *C. difficile* spores. However, none of the synthetic peptides, alone or in combination, effectively blocked E-cadherin binding in pull-down assays. However, a noteworthy observation is that the synthetic peptide against the CotE_1 motif located in between residues 44 and 61 of CotE was able to increase E-cadherin binding to *C. difficile* spores. Despite the lack of inhibition, this region seems to be implicated in E-cadherin binding and aggregation of CotE, explaining the increase in E-cadherin adherence. Moreover, several factors may explain the lack of effective inhibition, first, our linear peptides selected from microarray-mapped regions may lack the complex three-dimensional and conformational features present at native proteins, which may be needed for interactions. Another important consideration for why single synthetic peptides failed to inhibit is the target itself, the molecular interface of E-cadherin-spore binding is likely complex, complicating effective competitive inhibition by linear peptides. Together with the finding that E-cadherin binds nonhomogeneously to a subset of spores, these results raise the hypothesis that successful inhibition will likely require antibody-based strategies capable of targeting conformational epitopes within the *C. difficile* spore surface.

Collectively, our findings advance the understanding of *C. difficile* spore interactions with E-cadherin by identifying four candidate ligands, CotE and CdeM, as well as two previously uncharacterized proteins, which are conserved among clinically important clades and expand the known repertoire of spore surface proteins. Only a subset of spores, likely those with a thick exosporium, bind E-cadherin, highlighting phenotypic heterogeneity, with implications for colonization dynamics during infection. The comparable E-cadherin binding affinities observed across clades suggest that this interaction is a widely maintained mechanism that may contribute to spore persistence. Future work, including cross-linking mass spectrometry for detailed residue interface interactions, targeted mutagenesis, and in vivo studies, will be necessary to clarify the physiological significance and the specific contributions of each ligand to spore adherence and pathogenesis.

## Supplementary Material













## Data Availability

The mass spectrometry proteomics data have been deposited to the MassIVE repository (https://massive.ucsd.edu/ProteoSAFe/static/massive.jsp) with the data set identifier MSV000100416.

## Notes

Bacterial strains and growth conditions; purification of C. difficile spores; one-dimensional far-Western blot; two-dimensional (2D) far-Western blot; in-gel digestion of E-cadherin-binding protein spots; cloning of ligand candidates into overexpressing pET-16b vector; heterologous overexpression of ligand candidates in *E. coli*; one dimensional far-Western blot of recombinant candidate proteins with human E-cadherin; E-cadherin pull down binding assay with *C. difficile* spores; fluorescence microscopy of E-cadherin-binding to *C. difficile* spores; amino acid conservation of E-cadherin ligand candidates; peptide microarray assay; AlphaFold3 prediction and biophysical properties of ligands; inhibitory E-cadherin pull down binding assay with *C. difficile* R20291 spores and synthetic peptides; statistical analyses; analyses of E-cadherin binding to *C. difficile* R20291_CM196_ spores ;raw immunoblot and SDS-PAGE images for E-cadherin binding assays ; replicate analyses of E-cadherin-binding proteins in *C. difficile* R20291_CM196_ spore coat/exosporium extracts by two-dimensional FAR-Western blotting; raw immunoblot and SDS-PAGE images for CotE, CotA, CdeM, and CotB binding assays; raw immunoblot and SDS-PAGE images for CDIF27147_00632, CDIF27147_03838, CDIF27147_01111, and CDIF27147_02043 binding assays; raw immunoblot and SDS-PAGE images for CdeC, SipL, CDIF27147_00446, and CotJC1 binding assays; raw immunoblot and SDS-PAGE images for CDIF27147_02282 binding assays; replicate analysis of E-cadherin interaction with candidate *C. difficile* R20291_CM196_ spore surface proteins by Far-Western blotting; raw immunoblot and SDS-PAGE images for CotE, CotA, CdeM, and CotB binding assays; raw immunoblot and SDS-PAGE images for CDIF27147_00632, CDIF27147_03838, CDIF27147_01111, and CDIF27147_02043 binding assays; raw immunoblot and SDS-PAGE images for CdeC, SipL, CDIF27147_00446, and CotJC1 binding assays; raw immunoblot and SDS-PAGE images for CDIF27147_02282 binding assays; raw immunoblot and SDS-PAGE images for E-cadherin pull-down assays with spores from *C. difficile* clades 1–5; replicate analysis of E-cadherin binding by spore coat/exosporium extracts from diverse *C. difficile* clade strains; raw immunoblot and SDS-PAGE images for replicate E-cadherin binding assays with spore coat/exosporium extracts from *C. difficile* clade strains; secondary antibody only control on the conformational peptide microarray; conservation of candidate E-cadherin-binding motifs across *C. difficile* clades; raw immunoblot and SDS-PAGE images for peptide inhibition assays; raw immunoblot and SDS-PAGE images for peptide inhibition assays ; replicate gels of E-cadherin pull-down assays to evaluate effect of synthetic peptides on binding to *C. difficile* R20291_CM196_ spores; raw immunoblot and SDS-PAGE images for replicate E-cadherin pull-down assays with synthetic peptides; raw immunoblot and SDS-PAGE images for replicate E-cadherin pull-down assays with synthetic peptides; bacterial strains; primers used, and plasmids used (PDF)
MS/MS identification results for proteins isolated from E-cadherin immunoreactive spots (XLSX)
Filtered LC-MS/MS protein identifications and sigma factor assignment from SDS-PAGE Gel spots (XLSX)
Amino acid sequences of E-cadherin binding spore surface ligands (XLSX)
Genome accessions and metadata for EnteroBase *C. difficile* data set (XLSX)
Amino acid conservation of *C. difficile* spore surface proteins across five major *C. difficile* clades (XLSX)
