# Supplementary material for: Coupling Far-Western Blotting with Peptide Microarrays Reveals Novel E‑Cadherin Spore-Surface Ligands in Clostridioides difficile
Source: J Proteome Res. 2026 Jun 16;25(7):3487–506. doi: 10.1021/acs.jproteome.5c01166 (PMC13339774; doi:10.1021/acs.jproteome.5c01166)
Supplement: Supplementary file 1 [file pr5c01166_si_001.pdf]

Supporting Information

Coupling Far-western blotting with peptide microarray reveals novel E-cadherin spore-surface ligands in *Clostridioides difficile*.

Osiris K. Lopez-Garcia[1,2], Marjorie Pizarro-Guajardo[1], Klaudia I. Kocurek[3], Yohannes H. Rezenom[3] and Daniel Paredes-Sabja[1,2,4]*

[1]Department of Biology, Texas A&M University, College Station, Texas, 77843, U.S.A.

[2] Interdisciplinary Graduate Program in Genetics & Genomics, Texas A&M University, College Station, Texas, 77843, U.S.A.

[3] Department of Chemistry, Texas A&M University, College Station, Texas, 77843, U.S.A.

[4]Department of Biology, Texas A&M University, College Station, Texas, 77843, U.S.A. Email: dparedes-sabja@bio.tamu.edu

Table of Contents Supporting Information

Supporting Information Extended Materials and Methods

Figure S1. Analyses of E-cadherin binding to *C. difficile* R20291$_{CM196}$ spores

Figure S2. Raw immunoblot and SDS-PAGE images for E-cadherin binding assays shown in Figure 1A and Figure S1A-B

Figure S3. Replicate analyses of E-cadherin-binding proteins in *C. difficile* R20291$_{CM196}$ spore coat/exosporium extracts by two-dimensional FAR-Western blotting

Figure S4. Raw immunoblot and SDS-PAGE images for CotE, CotA, CdeM, and CotB binding assays shown in Figure 4

Figure S5. Raw immunoblot and SDS-PAGE images for CDIF27147_00632, CDIF27147_03838, CDIF27147_01111, and CDIF27147_02043 binding assays shown in Figure 4

Figure S6. Raw immunoblot and SDS-PAGE images for CdeC, SipL, CDIF27147_00446, and CotJC1 binding assays shown in Figure 4

Figure S7. Raw immunoblot and SDS-PAGE images for CDIF27147_02282 binding assays shown in Figure 4

Figure S8. Replicate analysis of E-cadherin interaction with candidate *C. difficile* R20291$_{CM196}$ spore surface proteins by Far-Western blotting

Figure S9. Raw immunoblot and SDS-PAGE images for CotE, CotA, CdeM, and CotB binding assays shown in Figure S8

Figure S10. Raw immunoblot and SDS-PAGE images for CDIF27147_00632, CDIF27147_03838, CDIF27147_01111, and CDIF27147_02043 binding assays shown in Figure S8

Figure S11. Raw immunoblot and SDS-PAGE images for CdeC, SipL, CDIF27147_00446, and CotJC1 binding assays shown in Figure S8

Figure S12. Raw immunoblot and SDS-PAGE images for CDIF27147_02282 binding assays shown in Figure S8

Figure S13. Raw immunoblot and SDS-PAGE images for E-cadherin pull-down assays with spores from *C. difficile* Clades 1-5 shown in Figure 6

Figure S14. Replicate analysis of E-cadherin binding by spore coat/exosporium extracts from diverse *C. difficile* Clade strains

Figure S15. Raw immunoblot and SDS-PAGE images for replicate E-cadherin binding assays with spore coat/exosporium extracts from *C. difficile* Clade strains shown in Figure S14

Figure S16. Secondary antibody only control on conformational peptide microarray

Figure S17. Conservation of candidate E-cadherin-binding motifs across *C. difficile* Clades

Figure S18. Raw immunoblot and SDS-PAGE images for peptide inhibition assays shown in Figure 9 (first set of constructs)

Figure S19. Raw immunoblot and SDS-PAGE images for peptide inhibition assays shown in Figure 9 (second set of constructs)

Figure S20. Replicate gels of E-cadherin pull-down assays to evaluate effect of synthetic peptides on binding to *C. difficile* $R20291_{CM196}$ spores

Figure S21. Raw immunoblot and SDS-PAGE images for replicate E-cadherin pull-down assays with synthetic peptides shown in Figure S20 (first set of constructs)

Figure S22. Raw immunoblot and SDS-PAGE images for replicate E-cadherin pull-down assays with synthetic peptides shown in Figure S20 (second set of constructs)

Tables S1. Bacterial strains used

Table S2. Primers used

Table S3. Plasmids used

Table S4. MS/MS identification results for proteins isolated from individual SDS-PAGE gel spots (xlsx)

Table S5. Filtered LC-MS/MS protein identifications and sigma factor assignment from SDS-PAGE Gel spots (xlsx)

Table S6. Amino acid sequences of E-cadherin binding spore surface ligands (xlsx)

Table S7. Genome accessions and metadata for EnteroBase *C. difficile* dataset (xlsx)

Table S8. Amino acid conservation of *C. difficile* spore surface proteins across five major *C. difficile* Clades (xlsx)

Extended material and methods

Bacterial strains and growth conditions.

C. difficile strains (Table S1) were grown at 37 °C under anaerobic conditions in a Coy anaerobic chamber (4% H2, 5% CO2, 85% N2) in BHIS medium: 3.7% (w/v) brain heart infusion broth supplemented with 0.5% (w/v) yeast extract (Difco) and 0.1% (w/v) L-cysteine or on 3.7% (w/v) BHIS agar plates. *Escherichia coli* strains (Table S1) were grown aerobically at 37 °C under aerobic conditions with shaking at 150 rpm in Luria-Bertani medium (BD), supplemented with 100 µg/mL ampicillin (Sigma), where appropriate.

Purification of *C. difficile* spores.

C. difficile strains were cultured anaerobically at 37 °C in BHIS plates supplemented with 0.5% yeast extract and 0.1% cysteine. To promote germination, overnight cultures were prepared in BHIS broth supplemented with 0.1% sodium taurocholate and 0.2% D-fructose. After overnight incubation, cultures were diluted to an OD_{600} of 0.5, and 150 µL were spread onto 70:30 sporulation medium plates, which were incubated anaerobically for 5 days at 37 °C. Sporulating cultures were harvested and resuspended in ice-cold Milli-Q water, then incubated overnight at 4 °C to lyse residual vegetative cells. Spores were collected by centrifugation at 14,000 rpm for

5 min, washed repeatedly with ice-cold Milli-Q water, and pelleted again until 99% purity free of vegetative cells and debris which was confirmed by microscopy. Final spore preparations were quantified using a Neubauer chamber and stored in 100 µL aliquots at -80 °C adjusted to 5×10^9 spores/mL until use.

One-dimensional far-western blot.

One-dimensional far-western blot assay using *C. difficile* spore coat/exosporium CHAPS buffer extracts was performed following far-western blot protocols with modifications [1,2]. Briefly, $5x10^8$ *C. difficile* spores were resuspended in 100 µL of CHAPS buffer (8 M Urea, 2 M Thiourea, 4% CHAPS and 65 mM DTT) and incubated at 37 °C for 90 min. After incubation, samples were pelleted down at 16,000 x g for 5 min and supernatant containing spore coat/exosporium extract was transferred to a new microcentrifuge tube for further processing. 25 µL of 4X Laemmli sample loading buffer was added to spore coat/exosporium extract, electrophoresed in 15% SDS-PAGE at 100 volts for ~1 h, and transferred onto nitrocellulose membrane (Bio-Rad) at 200 volts for 90 min. Nitrocellulose membrane was blocked with 5 mL of 3% bovine serum albumin (BSA) in TBST buffer (20 mM Tris-HCl, 150 mM NaCl, 0.05 % Tween 20) for 1 h at room temperature. Human E-cadherin recombinant protein (Advanced Biomatrix; Cat no. 5085) was diluted to 1 µg/mL and nitrocellulose membrane was incubated with 5 mL of human E-cadherin at 4 °C for 16 h, followed by three washes with 5 mL of TBST buffer for 5 min each. Next, membrane was incubated with 5 mL 1:1000 rabbit anti-E-cadherin (Abcam; Cat no. AB40772) for 4 h at room temperature, followed by three washes with 5 mL of TBST buffer for 5 min each, followed by incubation with 5 mL of 1:10,000 HRP-conjugated goat anti-rabbit IgG (Thermo; Cat no. 31460)

for 1 h. Immunoreactive proteins were detected using a chemiluminescent substrate (BioRad, Cat no. 17050610) and visualized with the chemiluminescence C-Digit Blot Scanner (LI-COR, USA).

Two-dimensional (2D) far-western blot.

A two-dimensional (2D) Far-Western blotting coupled with LC-MS/MS approach following the method above with the following additions. 200 µL of rehydration buffer (8 M Urea, 2 M Thiourea, 4% CHAPS, 0.002% bromophenol blue, 65 mM dithiothreitol (DTT) and 0.2% of pH 4-7 ampholytes) were added to *C. difficile* spores coat/exosporium extract (200 µL) equivalent to 4 × 10^9. After 1 h of incubation at 37 °C, 200 µL of the protein-containing sample was applied to a lane of the isoelectro-focusing (IEF) tray and overlaid with a 7 cm pH 4-7 ReadyStrip IPG strip (Bio-Rad), then incubated at room temperature until the rehydration buffer was fully absorbed (~1 h). The strip was covered with ~1 mL of mineral oil (Bio-Rad, Cat no. 1632129) and incubated overnight at room temperature in a humidified chamber. IPG strips were then placed in IPGphor (Pharmacia Biotech, Sweden) and subjected to IEF. The IEF steps for 7 cm IPG strips were (1) constant power, 0.1 watts for 1 h; (2) constant power, 0.5 watts with an upper voltage of 8,000 volts for 1 h; (3) constant voltage, 1,000 volts for 1 h. Before second dimension on SDS-PAGE, IPG strips were placed gel-side up on a cleaned hydration tray and equilibrated in equilibrium buffer containing 6 M Urea, 75 mM Tris-HCl pH 8.8, 29.3% glycerol, 2% SDS, and 0.002% bromophenol blue. Reduction, to break disulfide bonds and fully denature protein structure, was performed by incubating IPG strips with equilibration buffer containing 10 mg/mL DTT under gentle agitation for 10-15 min, followed by buffer removal. For alkylation, to prevent the reformation of disulfide bonds, strips were overlaid with 4 mL equilibration buffer containing 25 mg/mL iodoacetamide and incubated under gentle agitation for 10-15 min, followed by buffer

removal. IPG strips were then positioned on a 10 × 10 cm, 1.5 mm thick, 15% acrylamide gel and fixed using ~500 µL of 1% melted agarose containing 1× Tris-Glycine buffer (pH 8.0) and 0.001% Bromophenol Blue. Gels were electrophoresed at 50 mA for 4 h at 4 °C until dye reach end of the gel. Mirror gels were processed in parallel, with one stained using Coomassie Brilliant Blue G-250 colloidal (Bio-Rad) for protein visualization and the other subjected to Far-western blot analysis as described above.

In-gel digestion of E-cadherin-binding protein spots.

To identify E-cadherin ligands, immunoreactive spots were excised from SDS-PAGE gel and subjected to in-gel trypsin digestion. For these analyses, three independent 2D gels were processed: Gel 1 corresponds to the 2D FAR-Western shown in Fig. 2C, while Gels 2 and 3 correspond to the replicate blots shown in Fig. S2A and Fig. S2B, respectively. Gel fragments were transferred to 0.5 mL microcentrifuge tubes containing 100 µL of 100 mM ammonium bicarbonate; the solution was reduced to just cover the gel pieces before adding 10 µL of 50 mM DTT and incubating at 95 °C for 15 min. After cooling, 10 µL of 100 mM iodoacetamide was added, and samples were incubated for 30 min at room temperature in the dark. Gel pieces were destained and dehydrated by incubation with 400 µL of 50% acetonitrile/50 mM ammonium bicarbonate for 15 min, followed by replacement with 400 µL of 100% acetonitrile to fully dehydrate the gel, after which the liquid was removed and gels air-dried for 15-30 min. For proteolytic digestion, 20 µL of sequencing-grade modified trypsin (0.02 µg/µL in 25 mM ammonium bicarbonate; Promega, Cat. V5111) was added, and gels were rehydrated on ice for approximately 30 min. Sufficient 25 mM ammonium bicarbonate (~50 µL) was added to fully submerge the gel pieces, and tubes were sealed and incubated overnight at 37 °C in a humidified chamber. Peptide-containing supernatants

were collected and samples were analyzed by LC-MS/MS using an Ultimate 3000 nano-LC system coupled to an Orbitrap Fusion tribrid mass spectrometer (Thermo Scientific).

1 µl was injected onto and separated by a 150 × 0.075 mm column (Waters nanoEase M/Z Peptide BEH C18, 130 Å, 1.7 µm particle size) at a flow rate of 0.300 µl/min. The total duration of the method was 60 minutes, with the gradient set as follows: equilibration at 2% B (98% acetonitrile, 2% water, 0.1% formic acid), ramp to 45% B at 37 minutes, ramp to 90% B at 40 minutes and hold until 46 minutes, ramp down to 2% B at 47 minutes and hold at 2% B until the end of the run at 60 minutes. Eluent was introduced into the Fusion mass spectrometer by nano-ESI at a static voltage of 2450 V. Mass spectrometry data were acquired in positive mode at a resolution of 120,000 (at m/z 200) in the m/z range 400-1600. MS/MS data were acquired by HCD at a fixed collision energy of 28% with a precursor ion isolation window of 1.6 m/z; fragments were detected in the ion trap.

Data processing and analysis was performed in Proteome Discoverer 2.4 (Thermo Scientific) using *C. difficile* strain R20291 reference CP029423 as the database. The search was performed using Sequest HT as the engine and Percolator for FDR estimation, with trypsin enzyme specificity, and up to 2 allowed missed cleavages. Precursor and fragment mass tolerances were set to 10 ppm and 0.6 Da, respectively. N-terminal methionine cleavage and acetylation were considered, with cysteine carbamidomethylation set as a fixed modification. The FDR thresholds used were 1% (high confidence) and 5% (medium confidence) and protein grouping and reporting rules are described together with the criteria for inclusion in Tables S4 and S5.

Cloning of ligand candidates into overexpressing pET-16b vector.

Primer design and amplification of *C. difficile* R20291 strain were based on the available *C. difficile* genome from the NCBI databases with accession number CP029423. Candidate genes were inserted downstream of His-tag sequence of pET-16b utilizing restriction enzymes NdeI and BamHI, which render a N-terminal His-tagged protein. DNA fragments encoding candidate gene sequences were amplified using the primers listed in Table S2. Genomic DNA was obtained from an overnight culture of *C. difficile* R20291 strain and isolated using a Monarch Genomic DNA purification kit (New England Biolabs). The candidate genes were PCR-amplified with Phusion High-Fidelity DNA polymerase (New England Biolabs) using 1 μg of chromosomal DNA. The PCR product was recovered from agarose gel and cleaned with the Zymoclean Gel DNA Recovery Kit (Zymo). The pET-16b vector was digested with BamHI/NdeI restriction endonucleases, and the digested plasmid and PCR product were ligated by Gibson Assembly [3]. The resulting reaction was transformed into the bacterial strain *E. coli* BL21 (DE3) pRIL using heat shock transformation standard procedures. Transformant cells were plated on LB plates containing 100 μg/mL ampicillin and incubated overnight at 37 °C. Plasmid DNA was isolated using Phenol-Chloroform Extraction standard protocol [4,5]. Insertion of candidates genes was confirmed by restriction enzyme digestion using BamHI and NdeI, then positive plasmids for each candidate gene were sequence Oxford Nanopore Technology by Plasmidsaurus Inc. (Eugene, OR).

Heterologous overexpression of ligand candidates in *E. coli.*

Transformed *E. coli* strains containing pET-16b plasmids with candidates genes (Table S3) as well as empty vector control were grown at 37 °C in 50 mL LB medium supplemented with 100 μg/mL ampicillin, 35 μg/mL chloramphenicol and 0.5% glucose until OD_{600} = 0.5, induced with

a final concentration of 1 mM IPTG and further grown for 16 h at 37 °C. After incubation, cultures were pelleted down at 8,000 x g for 10 min at 4 °C. The separation of soluble and insoluble protein fractions was performed as follows. Cell pellets were resuspended in 150 µL soluble buffer (50 mM Tris-HCl (pH 8.0), 500 mM NaCl, 40 mM imidazole, 0.1% Triton X-100, 2.5 mM β-mercaptoethanol, and 30% glycerol) and subjected to sonication at 30 watts for 15 s, followed by cooling on ice for 30 s. This sonication and cooling cycle was repeated five times to ensure cell lysis. The lysate was then centrifuged at 16,000 x g for 10 min, and the supernatant was collected (~ 100 µL) and designated as the soluble fraction. The remaining pellet was resuspended in 150 µL insoluble buffer (20 mM Tris-HCl (pH 7.8), 500 mM NaCl, 5 mM imidazole, and 8 M urea) and subjected to an identical sonication and cooling procedure, consisting of five cycles of 15 s of sonication at 30 watts followed by 30 s of cooling on ice. The insoluble-lysate was again centrifuged at 16,000 x g for 10 min, and the resulting supernatant (~ 100 µL) was collected and designated as the insoluble fraction. Both fractions were aliquoted and stored at -20 °C until further analysis.

One dimensional far-western blot of recombinant candidate proteins with human E-cadherin.

Soluble and insoluble fractions of overexpressed ligands as well as empty vector control were processed to assess expression and solubility of recombinant proteins. Fractions were diluted and resuspended in 2X Laemmli sample loading buffer, boiled for 5 min, centrifuged at 16,000 x g for 1 min and separated by electrophoresis on 12% acrylamide gels alongside PageRuler Plus prestained protein ladder (Thermo Fisher) at 100 volts until dye reached end of gel (~ 90 min). Proteins were transferred onto nitrocellulose membranes (Bio-Rad) and blocked for 1 h at room

temperature with 3% BSA in 0.1% Tween-20 Tris-buffered saline (T-TBS). Next, membranes were incubated with 5 mL of 1 μg/mL human recombinant E-cadherin protein (Advanced Biomatrix; Cat no. 5085) in 1% BSA in TBS. Overnight at 4 °C. Following three 5 min washes with TBST, membranes were incubated with 1:1000 rabbit anti-E-cadherin antibody (Abcam; Cat no. AB40772) for 4 h at room temperature or overnight at 4 °C, washed three times, and then incubated with 1:10,000 HRP-conjugated goat anti-rabbit IgG antibody (Thermo; Cat no. 31460) for 1 h at room temperature.

For detection of overexpressed His-tagged proteins, membranes were incubated with 1:1,000 mouse 6x-His Tag monoclonal antibody (Invitrogen; Cat no. MA1-21315) for 3 h at room temperature, washed, and then incubated with 1:10,000 HRP-conjugated goat anti-mouse IgG secondary antibody (Rockland; Cat no. 610-1302). Immunoreactive proteins were detected using a chemiluminescent substrate (BioRad, Cat no. 17050610) and visualized with the chemiluminescence C-Digit Blot Scanner (LI-COR, USA).

E-cadherin pull down binding assay with *C. difficile* spores.

Binding affinity of human E-cadherin to *C. difficile* spores from the epidemic R20291 strain were analyzed using modified protocols adapted previously described methods [6]. Twenty microliters of spore suspensions (1×10^{8} spores) were pelleted down at 16,000 x g for 5 min at room temperature, resuspended in 40 μL of PBS-T 0.2% BSA in PBS-T containing 1-20 μg/mL of recombinant human E-cadherin (Advanced Biomatrix; Cat no. 5085) and incubated for 90 min at 37 °C. Spores were washed three times with 40 μL of PBS after centrifuging at 16,000 x g for min and the pellet resuspended in 40 μL of 2X Laemmli sample loading buffer, boiled for 5 min, centrifuged at

16,000 x g for 5 min. Spore coat/exosporium extracts containing E-cadherin (20 μL) were electrophoresed on 12% SDS-PAGE gels for ~1 h at 100 volts. Proteins were transferred to nitrocellulose membranes (Bio-Rad) at 200 volts for 90 min and blocked for 1 h at room temperature with 3% BSA in 0.1% Tween-20 Tris-buffered saline (T-TBS). Membranes were probed with 1:1,000 rabbit anti-E-cadherin antibody (Abcam; Cat no. AB40772) in T-TBS containing 1% BSA for 1 h at room temperature, rinsing three times followed by incubation with 1:10,000 goat anti-rabbit IgG HRP (Thermo; Cat no. 31460) in 1% BSA TTBS for 3 h at room temperature. Immunoreactive proteins were detected using a chemiluminescent substrate (BioRad, Cat no. 17050610) and visualized with the chemiluminescence C-Digit Blot Scanner (LI-COR, USA).

Quantitative densitometry analysis of E-cadherin binding to *C. difficile* spores was performed using ImageJ software (v. 1.54p). Immunoblots images were converted to grayscale, and the region of interest (ROI) corresponding to each immunoreactive band was outlined using the rectangle selection tool. The integrated density (sum of pixel values) within each ROI was measured, reflecting the amount of E-cadherin detected by antibody. Background intensity was measured in an adjacent area free of signal and subtracted from each sample's value to correct for nonspecific binding or blot artifacts. The dissociation constant (K_d) was determined by fitting densitometric values of E-cadherin binding at different protein concentrations to a saturation binding model and K_D is the dissociation equilibrium constant quantified as previously described [7,8].

For the E-cadherin pull down assay, spores were prepared and purified as described above from *C. difficile* strains of the five classical Clades (C1-C5; Table S1) [9,10]. Spore coat/exosporium

extracts were prepared as previously described. Far-Western blotting was performed as outlined above. To compare E-cadherin binding of Clade strains, spores from strain R20291$_{CM196}$ were included as control. Briefly, nitrocellulose membranes were incubated with 1 µg/mL human recombinant E-cadherin protein (Advanced Biomatrix; Cat no. 5085) overnight at 4 °C. After three 5 min washes with TBST, membranes were incubated with 1:1000 rabbit anti-E-cadherin antibody (Abcam; Cat no. AB40772) for 4 h at room temperature or overnight at 4 °C, followed by three additional washes. Membranes were then incubated with 1:10,000 HRP-conjugated goat anti-rabbit IgG antibody (Thermo; Cat no. 31460) for 1 h at room temperature before detection.

Fluorescence microscopy of E-cadherin-binding to *C. difficile* spores.

Twenty microliters of *C. difficile* spores (1×10^8 total) were incubated for 1 h at 37 °C in 40 µL of PBS with 0.2% bovine serum albumin (BSA) containing 0, 2, 4, 6, or 8 µg/mL of recombinant human E-cadherin (Advanced Biomatrix; Cat no. 5085). After incubation, spores were washed three times with PBS with 0.2% BSA after centrifugation at 16,000 x g for 5 min. Spores were then blocked with 2% BSA for 1 h at room temperature and incubated overnight at 4 °C with 1:100 rabbit anti-E-cadherin (Abcam; Cat no. AB40772) in PBS-1% BSA. After incubation, spores were washed three times with PBS with 0.2% BSA after centrifugation at 13,000 x g for 5 min, and incubated for 1 h at room temperature with 1:400 anti-rabbit IgG Alexa Fluor 568 (Abcam; Cat no. AB175692) in PBS-1% BSA. After incubation, spores were washed three times with PBS with 0.2% BSA after centrifugation at 16, 000 x g for 5 min and resuspended in 20 µL of PBS. 5 µL of samples were spread on sterile coverslips air-dried at room temperature for 30 min. Coverslips were mounted using ProLong Diamond Antifade Mountant (Invitrogen), incubated overnight at 4 °C and then sealed with nail polish.

Imaging was performed using a Leica DMRX fluorescence microscope equipped with a Hamamatsu ORCA-Fusion BT (C15440-20UP) Camera. Phase-contrast images (2546 × 2546 pixels) were acquired with a 150 ms exposure. Fluorescent images were captured using Chroma ET-mCherry/Texas Red filter set (Cat no. 49008; exciter ET560/40x, dichroic T585lpxr, emitter ET630/75m; Chroma Technology Corp.) with 1 s of exposure time. Negative controls included spores incubated without E-cadherin or primary antibody, which were also imaged with 1 s of exposure time.

Spore segmentation was carried out in Fiji using the MicrobeJ plugin, with “Fit shape” set to Rod-shaped. Morphology filters were defined as area 100-max and length 10-max with width, circularity, curvature, sinuosity, angularity, solidity, and intensity all set to 0-max. “Exclude on edges” was enabled to remove partial objects at image boundaries, and “Shape descriptors” were selected to export morphology features for each segmented particle. For intensity quantification, the mean gray value within each particle ROI was measured and background-corrected by subtracting the mean gray value of the local background (reported as corrected mean intensity, mean_c). All parameters were held constant across the dataset to ensure consistent detection and measurement across images and conditions. The “Rod-shaped” setting was chosen empirically because MicrobeJ’s axial model improves segmentation and descriptor extraction even for nearly round spores, without forcing elongation, and performed more reliably than the “round” template on these data.

Amino acid conservation of E-cadherin ligand candidates.

To assess amino acid conservation of ligand protein candidates CotE (CDIF27147_01458), CdeM (CDIF27147_01682), CDIF27147_03838 and CDIF27147_02282 within *C. difficile* Clades a small database was produced to include 250 *C. difficile* isolates with 50 isolated from each of the five classical Clades extracted from published dataset (Table S7) [9]. For each candidate protein, tBLASTn searches were run in Geneious Prime (2025.1.1, 2025) using the BLAST plugin querying the amino acid sequences (Table S6) encoded in reference genome *C. difficile* R20291 (CDR20291_CP029423) against the 250 *C. difficile* isolate database. Search settings were program = tBLASTn with matrix = BLOSUM45, word size = 2, gap open/ext = 15/2, low-complexity filtering =off, e-value threshold = 0.05. For each strain, percent pairwise identity and query coverage were exported from the Geneious tBLASTn results table and compiled for downstream analysis. Pairwise identity values for full-length hits were summarized per Clade (Table S8). After completing the analyses described above, binding motif sequences were extracted from the sequences obtained from each genome. Amino acid conservation was then assessed based on percentage identity to evaluate motif-level sequence conservation across strains.

Peptide microarray assay.

Conformational peptide microarrays were performed by PEPperPRINT (PEPperPRINT GmbH, Heidelberg, Germany) and designed using sequences from six proteins (Table S6): peroxiredoxin-chitinase (CotE, **CDIF27147_01282**), exosporium morphogenetic protein (CdeM, **CDIF27147_01682**), flavin reductase-like domain-containing protein (**CDIF27147_02282**), YmaF family protein (**CDIF27147_03838**), exosporium glycoprotein (C-terminal domain of BclA2), and collagen-like exosporium glycoprotein BclA3 (C-terminal domain of BclA3) [11]. Each protein sequence was elongated with neutral GSGSGSG linkers at both termini to minimize

truncated peptide artifacts. These sequences were converted into overlapping 9-mer and 13-mer peptides with overlaps of 8 and 12 amino acids, respectively. Peptides were cyclized on-chip via thioether bonds between modified N-termini and C-terminal cysteine residues [11,12]. The final microarrays contained 2,972 cyclic peptides printed in duplicate, framed by additional HA control peptides for assay validation.

Recombinant human E-cadherin (AA 1-82 with GST tag; Antibodies Online; Cat no. ABIN1348882) was diluted to concentrations of 1 μg/mL and 10 μg/mL in PBS (pH 7.4) with 0.005% Tween-20 and 10% blocking buffer (Rockland, Cat no. MB-070). Microarrays were pre-blocked with blocking buffer (Rockland, Cat no. MB-070) for 30 min and then incubated with E-cadherin solutions for 16 h at 4 °C under orbital shaking at 140 rpm. After incubation, arrays were washed twice for 10 s each with PBS-Tween and stained with rabbit anti-GST DyLight680 (0.1 μg/mL) and mouse anti-HA DyLight800 (0.2 μg/mL) for 45 min at room temperature.

Microarrays were scanned using Innopsys InnoScan 710-IR Microarray Scanner (Innopsys Inc; Illinois, USA); scanning resolution of 20 μm; scanning gain of 50 at low laser power (680 nm, red) and 10 at high laser power (800 nm, green) with fluorescence detection optimized for DyLight680 and DyLight800 dyes. Spot intensities were quantified using PepSlide Analyzer software (SICASYS Software GmbH, Heidelberg, Germany), which calculated median foreground intensities, background signals, and spot-to-spot deviations for duplicates. A maximum deviation threshold of 40% was applied to ensure data quality; spots exceeding this threshold were manually flagged as artifacts or validated as accurate data points. Intensity maps were generated using a red-to-white color scale to visualize interaction motifs, which were mapped onto protein

sequences to identify binding sites. Ambiguous residues within interaction motifs were annotated in gray for clarity. Baseline adjustments were applied to intensity plots to enhance visualization of signal-to-noise ratios across the peptide sequences. Quality controls included pre-staining microarrays with secondary antibodies to assess background interactions and staining HA control peptides to validate assay performance and microarray integrity. All assays were conducted with duplicate spots to ensure reproducibility.

AlphaFold3 prediction and biophysical properties of ligands.

The amino acid sequences of ligand protein candidates were extracted from the *C. difficile* R20291 reference genome (CP029423) and submitted to AlphaFold3 using single-chain mode with five stochastic seeds and three recycles per target [13]. Templates were left on default discovery, with automatic MSA generation; models were ranked by pLDDT. Predicted structures were saved alongside confidence metrics (pLDDT per residue), and top model was used in UCSF ChimeraX 1.10.1 (2025) was used for structure visualization, surface coloring, and figure generation [14]. Predicted binding sites defined by peptide microarray positive regions were mapped onto each ligand candidate by highlighting sequence with distinct colors to distinguish sites within or across proteins. Electrostatic potential on molecular surface was visualized using Coulombic surface coloring (blue = positive, white = neutral, red = negative), calculations were done using Amber 20 recommended default charges and atom types for standard residues, and hydrophobicity was displayed using the molecular lipophilicity potential scheme (blue = hydrophilic, white = neutral, yellow = hydrophobic.

Isoelectric point (pI) and net charge at specific residue sites were calculated using Prot pi | Protein Tool (https://protpi.ch; v. 2.2.29.152) [15]. Isolelectric point (pI) and local net charge was calculated at defined pH 7.4 using the ProMoST pKa set and default temperature/ionic-strength assumptions; cysteines were treated as reduced, N-termini were set as free amines, and C-termini as free carboxylates. Hydropathy profiles were generated with ProtScale (https://web.expasy.org/protscale/) using the Kyte-Doolitle amino acid scale, window size 9, no normalization of the scale, and linear N- to C-terminal indexing [16].

Prediction of intrinsically disordered regions were predicted using the PSIPRED Protein Analysis Workbench (http://bioinf.cs.ucl.ac.uk/psipred) [17], using the DISOPRED3 pipeline with default parameters: three iterations of PSI-BLAST against UniRef90 to compute PSSM profiles (E-value threshold 0.001), followed by the DISOPRED3 support-vector machine classifier to assign per-residue disorder probabilities. Residues with disorder probability ≥ 0.5 over contiguous windows ≥ 20 residues were called disordered.

Inhibitory E-cadherin pull down binding assay with *C. difficile* R20291 spores and synthetic peptides.

Synthetic peptides were designed based on high-affinity E-cadherin binding motifs identified from peptide microarray mapping of candidate spore surface proteins as regions with fluorescence intensity >100 arbitrary units. Peptide sequences of 9-20 amino acids corresponding to E-cadherin binding motifs as identified by peptide microarray were selected for synthesis. The scrambled peptide was generated by inputting the amino acid sequence of the highest binding motif into a scramble sequence tool, which randomly rearranges the order of the amino acids while preserving

the original composition. This approach creates a control peptide with the same residues but in a randomized sequence, allowing assessment of the sequence specificity of binding. All peptides, including scrambled control, were custom synthesized through solid-phase peptide synthesis by Alan Scientific (Maryland, USA). Recombinant human E-cadherin (Advanced Biomatrix; Cat no. 5085) was diluted to concentration of 200 nM with PBS and further diluted 1:1 with peptides to a final volume of 40 µL, resulting in a final E-cadherin concentration of 100 nM and final peptide concentration of 1, 5, 10 or 15 µM. E-cadherin-peptide mixtures were incubated for 2 h at 37 °C. Spores (1×10^8) were prepared in 0.2% BSA, pelleted, and resuspended in 40 µL of the E-cadherin-peptide mixtures, followed by incubation for 1 h at 37 °C.

Post incubation, spores were washed three times with PBS and resuspended in 40 µL 2X Laemmli loading buffer. Samples were boiled for 5 min, then subjected to SDS-PAGE using 12% acrylamide gels. Proteins were transferred onto nitrocellulose membranes blocked with 3% BSA in 0.1% Tween-20 TBS (TTBS) for 1 h at room temperature. Membranes were incubated overnight at 4 °C with 1:1000 rabbit anti-E-cadherin antibody (Abcam; Cat no. AB40772), washed thrice with TTBS, then incubated with 1:10,000 HRP-conjugated goat anti-rabbit IgG secondary antibody (Thermo; Cat no. 31460) for 3 h at room temperature. Chemiluminescent detection was performed according to the manufacturer's instructions.

Statistical analyses.

All statistical analyses and graph generation were performed in GraphPad Prism 10 (version 10.5.0). Dose-response binding experiments were analyzed by nonlinear regression with a single-site binding model to estimate dissociation constants (K_d), presented as mean ± SEM. Comparisons

of fluorescence intensities, percent binding, and inhibition by synthetic peptides were evaluated using one-way ANOVA with Šídák's or Tukey's multiple comparisons post hoc tests for more than two groups, while Welch's t test was applied for two-group comparisons with unequal variances. Delimitation of negative and positive E-cadherin bound spores was defined using a threshold of $\mu + 2.576 \times \sigma$ from control values. Distributions were assesed for normality using Q-Q plots, and frequency distributions were visualized with histogram plots. Percent pairwise identity of candidate E-cadherin-binding proteins among *C. difficile* Clades was compared by one-way ANOVA and multiple comparisons testing. Quantification of E-cadherin binding to spore surface proteins from different Clade strains was statistically analyzed using Šídák's multiple comparisons test, and results were plotted as relative fold changes ± SD from biological replicates. A significance level of $P < 0.05$ was used for all analyses.

References

1 Pizarro-Guajardo, M., Ravanal, M. C., Paez, M. D., Callegari, E. & Paredes-Sabja, D. Identification of Clostridium difficile Immunoreactive Spore Proteins of the Epidemic Strain R20291. *Proteomics Clin Appl* **12**, e1700182 (2018). https://doi.org:10.1002/prca.201700182

2 Wu, Y., Li, Q. & Chen, X. Z. Detecting protein-protein interactions by Far western blotting. *Nat Protoc* **2**, 3278-3284 (2007). https://doi.org:10.1038/nprot.2007.459

3 Gibson, D. G. *et al.* Enzymatic assembly of DNA molecules up to several hundred kilobases. *Nat Methods* **6**, 343-345 (2009). https://doi.org:10.1038/nmeth.1318

4 Birnboim, H. C. & Doly, J. A rapid alkaline extraction procedure for screening recombinant plasmid DNA. *Nucleic Acids Res* **7**, 1513-1523 (1979). https://doi.org:10.1093/nar/7.6.1513

5 Sambrook, J. & Russell, D. W. Purification of nucleic acids by extraction with phenol:chloroform. *CSH Protoc* **2006** (2006). https://doi.org:10.1101/pdb.prot4455

6 Castro-Córdova, P. *et al.* Redistribution of the Novel Clostridioides difficile Spore Adherence Receptor E-Cadherin by TcdA and TcdB Increases Spore Binding to Adherens Junctions. *Infect Immun* **91**, e0047622 (2023). https://doi.org:10.1128/iai.00476-22

7 Mora-Uribe, P. *et al.* Characterization of the Adherence of. *Front Cell Infect Microbiol* **6**, 99 (2016). https://doi.org:10.3389/fcimb.2016.00099

8 Xue, Q. *et al.* Bacillus anthracis spore entry into epithelial cells is an actin-dependent process requiring c-Src and PI3K. *PLoS One* **5**, e11665 (2010). https://doi.org:10.1371/journal.pone.0011665

9 Guerrero-Araya, E., Cid-Rojas, F., Muñoz, M., Rodríguez, C. & Paredes-Sabja, D. Identification of Novel Cryptic and Classical Clades in Clostridioides difficile. *bioRxiv*, 2025.2008.2001.668003 (2025). https://doi.org:10.1101/2025.08.01.668003

10 Knight, D. R. *et al.* Major genetic discontinuity and novel toxigenic species in Clostridioides difficile taxonomy. *Elife* **10** (2021). https://doi.org:10.7554/eLife.64325

11 Stadler, V. *et al.* Combinatorial synthesis of peptide arrays with a laser printer. *Angew Chem Int Ed Engl* **47**, 7132-7135 (2008). https://doi.org:10.1002/anie.200801616

12 Iwasaki, K., Goto, Y., Katoh, T. & Suga, H. Selective thioether macrocyclization of peptides having the N-terminal 2-chloroacetyl group and competing two or three cysteine residues in translation. *Org Biomol Chem* **10**, 5783-5786 (2012). https://doi.org:10.1039/c2ob25306b

13 Abramson, J. *et al.* Accurate structure prediction of biomolecular interactions with AlphaFold 3. *Nature* **630**, 493-500 (2024). https://doi.org:10.1038/s41586-024-07487-w

14 Pettersen, E. F. *et al.* UCSF ChimeraX: Structure visualization for researchers, educators, and developers. *Protein Sci* **30**, 70-82 (2021). https://doi.org:10.1002/pro.3943

15 Josuran, R. *Prot pi | Bioinformatics Calculator*, <https://www.protpi.ch/Calculator/ProteinTool> (2014).

16 Kyte, J. & Doolittle, R. F. A simple method for displaying the hydropathic character of a protein. *J Mol Biol* **157**, 105-132 (1982). https://doi.org:10.1016/0022-2836(82)90515-0

17 Buchan, D. W. A. & Jones, D. T. The PSIPRED Protein Analysis Workbench: 20 years on. *Nucleic Acids Res* **47**, W402-W407 (2019). https://doi.org:10.1093/nar/gkz297

Supplementary Figures

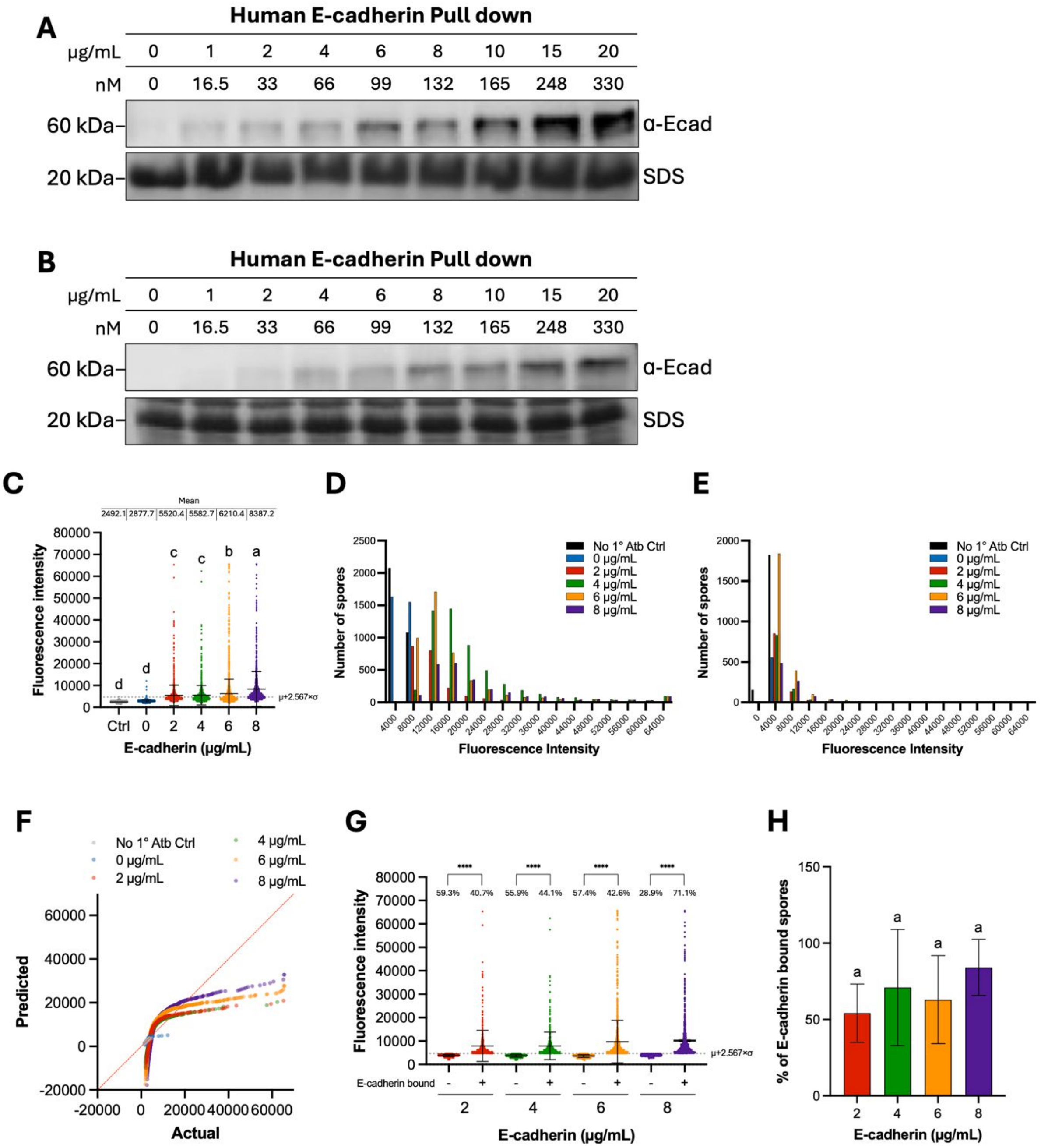

Figure S1 | Analyses of E-cadherin binding to *C. difficile* R20291$_{CM196}$ spores. (A-B) Representative images from two additional biological replicates of the binding assay shown in Figure 1A. *C. difficile* R20291$_{CM196}$ spores were incubated with increasing concentrations of recombinant human E-cadherin, washed, and processed for Far-Western blotting with anti-E-cadherin antibody detection. Molecular weight markers are indicated in kDa. (C) Fluorescence intensity quantified per spore for each condition in one replicate, analyzed using MicrobeJ in ImageJ for replicate 1. Scatter plots show distribution of fluorescence intensities for each treatment; mean is shown for each condition, and statistical significance was determined using

Šídák's multiple comparisons test. Groups not sharing a letter are significantly different ($P < 0.05$) as determined by multiple comparison test. Groups sharing one or more letters are not significantly different. Threshold for E-cadherin bound spores was calculated as $\mu+2.576\times\sigma$ (4698.9) using values from E-cadherin 0 µg/mL and is shown as grey dotted line. (D) Frequency distribution histogram depicting spore counts versus fluorescence intensity for replicate 1, and (E) frequency distribution histogram for replicate 2, showing concentration-dependent increases in binding distribution for each experiment. (F) Normal Q-Q plot comparing predicted versus actual values of E-cadherin fluorescence intensity across samples treated with varying concentrations (0-8 µg/mL) and no primary antibody control for replicate 1. Each sample group is represented by a distinct color as indicated in the legend. The dashed red line denotes the reference for perfect agreement between predicted and actual values. Deviations from the line indicate departures from normality for each condition. (G) Quantification of E-cadherin fluorescence intensity per spore across samples in one replicate divided based on whether spores were negative or positive for E-cadherin binding, statistical significance was determined using Welch's t test (****, $P < 0.0001$) for replicate 1. Threshold for E-cadherin bound spores was calculated as $\mu+2.576\times\sigma$ and is shown as grey dotted line. Percentages correspond to the proportion of E-cadherin bound positive or negative spores out of total spores. (H) Bar graph showing the percentage of E-cadherin-bound spores at 2, 4, 6 or 8 µg/mL of E-cadherin based on threshold (R1 = 9525.1, R2 = 4698.9), and statistical significance was determined using Tukey's multiple comparisons test. Groups not sharing a letter are significantly different ($P < 0.05$) as determined by multiple comparison test. Groups sharing one or more letters are not significantly different.

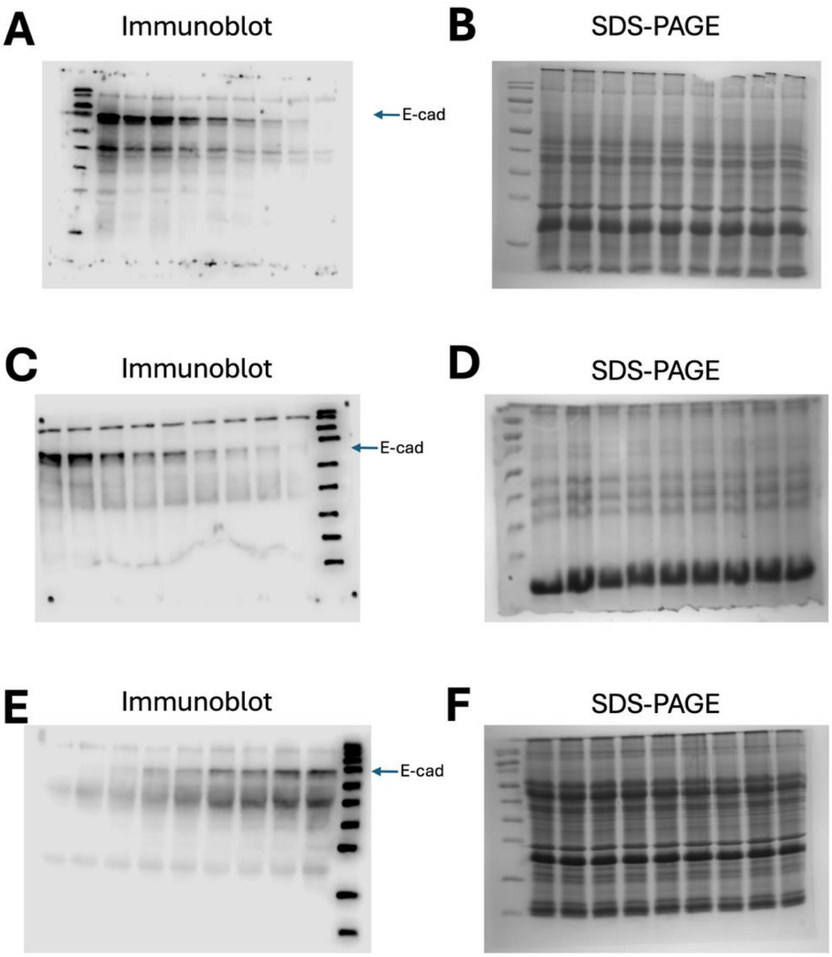

Figure S2 | Raw immunoblot and SDS-PAGE images for E-cadherin binding assays shown in Figure 1A and Figure S1A-B. (A,B) Raw chemiluminescent immunoblot (A) and corresponding Coomassie-stained SDS-PAGE gel (B) of *C. difficile* R20291$_{CM196}$ spore surface proteins incubated with increasing concentrations of recombinant human E-cadherin, corresponding to the pull-down experiment quantified in Figure 1A. The immunoblot was probed with anti-E-cadherin antibody (α-E-cad), and the arrow indicates the E-cadherin band. (C,D) Raw chemiluminescent immunoblot (C) and corresponding Coomassie-stained SDS-PAGE gel (D) from the biological replicate whose processed data are shown in Figure S1A. (E,F) Raw chemiluminescent immunoblot (E) and corresponding Coomassie-stained SDS-PAGE gel (F) from the biological replicate whose processed data are shown in Figure S1B.

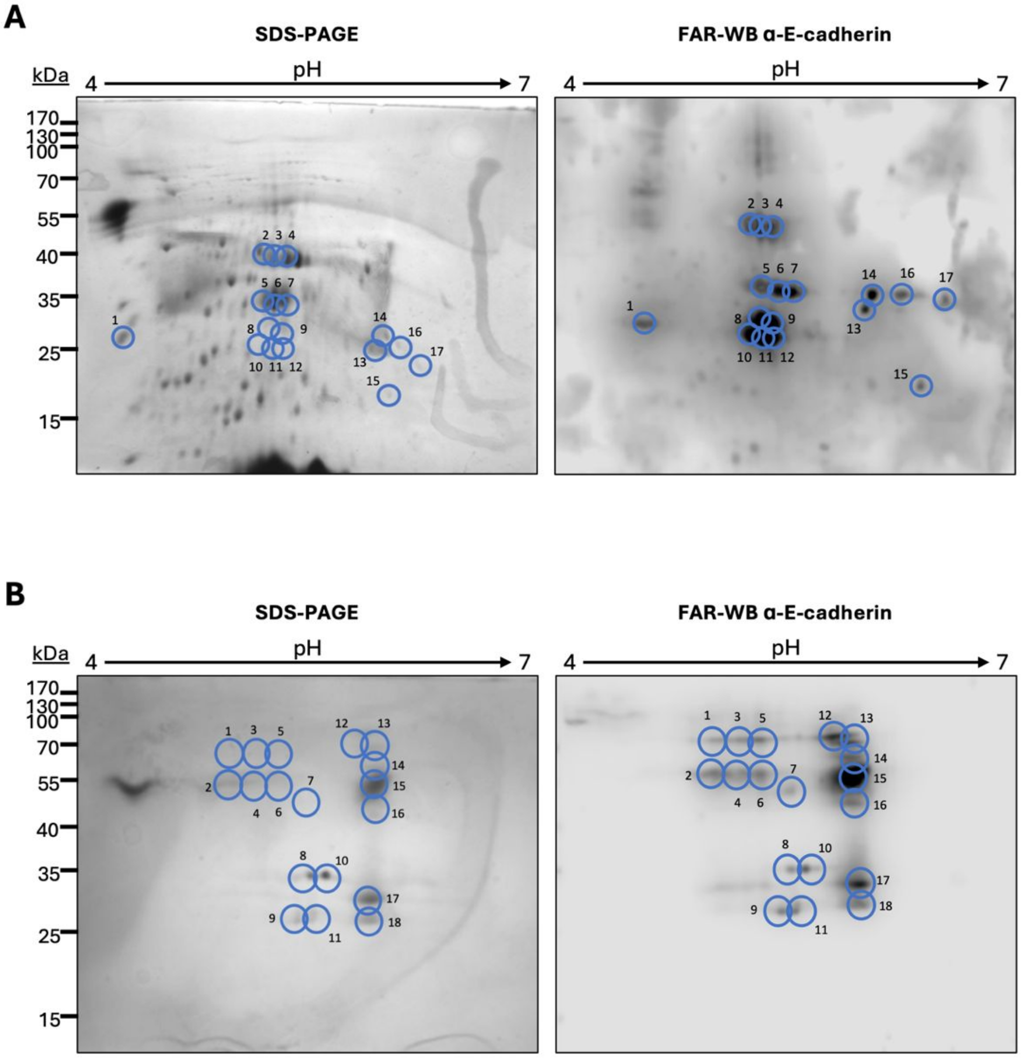

Figure S3 | Replicate analyses of E-cadherin-binding proteins in *C. difficile* $R20291_{CM196}$ spore coat/exosporium extracts by two-dimensional FAR-Western blotting. (A) Two-dimensional SDS-PAGE and FAR-Western blot of second biological replicate. Left: Coomassie-stained 2D gel showing protein separation by isoelectric focusing (pH 4-7) and SDS-PAGE with molecular weight markers (kDa) indicated. Right: Corresponding 2D FAR-Western blot, probed with anti-E-cadherin antibody, highlighting immunoreactive protein spots (numbered 1-17). (B) Two-dimensional SDS-PAGE and FAR-Western blot of third biological replicate. Left: Coomassie-stained 2D gel with separated proteins. Right: 2D FAR-Western blot showing immunoreactive spots detected using anti-E-cadherin antibody. Molecular weight markers and pH range (4-7) are shown; immunoreactive spots are indicated (numbered 1-18).

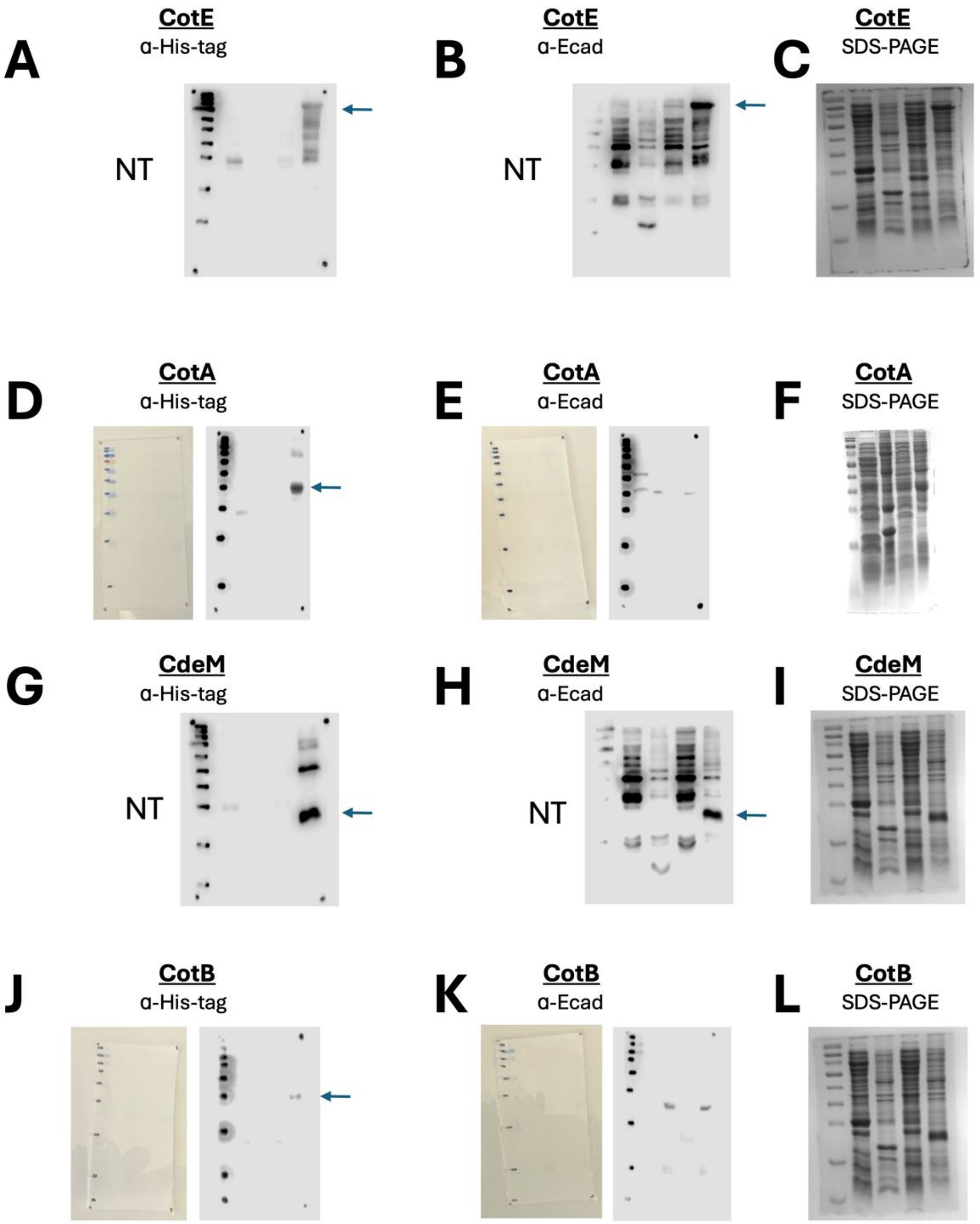

Figure S4 | Raw immunoblot and SDS-PAGE images for CotE, CotA, CdeM, and CotB binding assays shown in Figure 4. (A-C) Raw anti-His-tag immunoblot (A), anti-E-cadherin immunoblot (B), and SDS-PAGE gel (C) for purified His-tagged CotE, with arrows indicating the CotE band or E-cadherin-bound species; NT indicates that the corresponding nitrocellulose membrane image was not taken. (D-F) Raw anti-His-tag immunoblot (D), anti-E-cadherin immunoblot (E), and SDS-PAGE gel (F) for purified His-tagged CotA. (G-I) Raw anti-His-tag immunoblot (G), anti-E-cadherin immunoblot (H), and SDS-PAGE gel (I) for purified His-tagged CdeM. (J-L) Raw anti-His-tag immunoblot (J), anti-E-cadherin immunoblot (K), and SDS-PAGE gel (L) for purified His-tagged CotB. SDS-PAGE gels (C, F, I, L) show total protein loading and molecular weight marker lanes for the samples in the corresponding immunoblots, underlying the processed data presented in Figure 4.

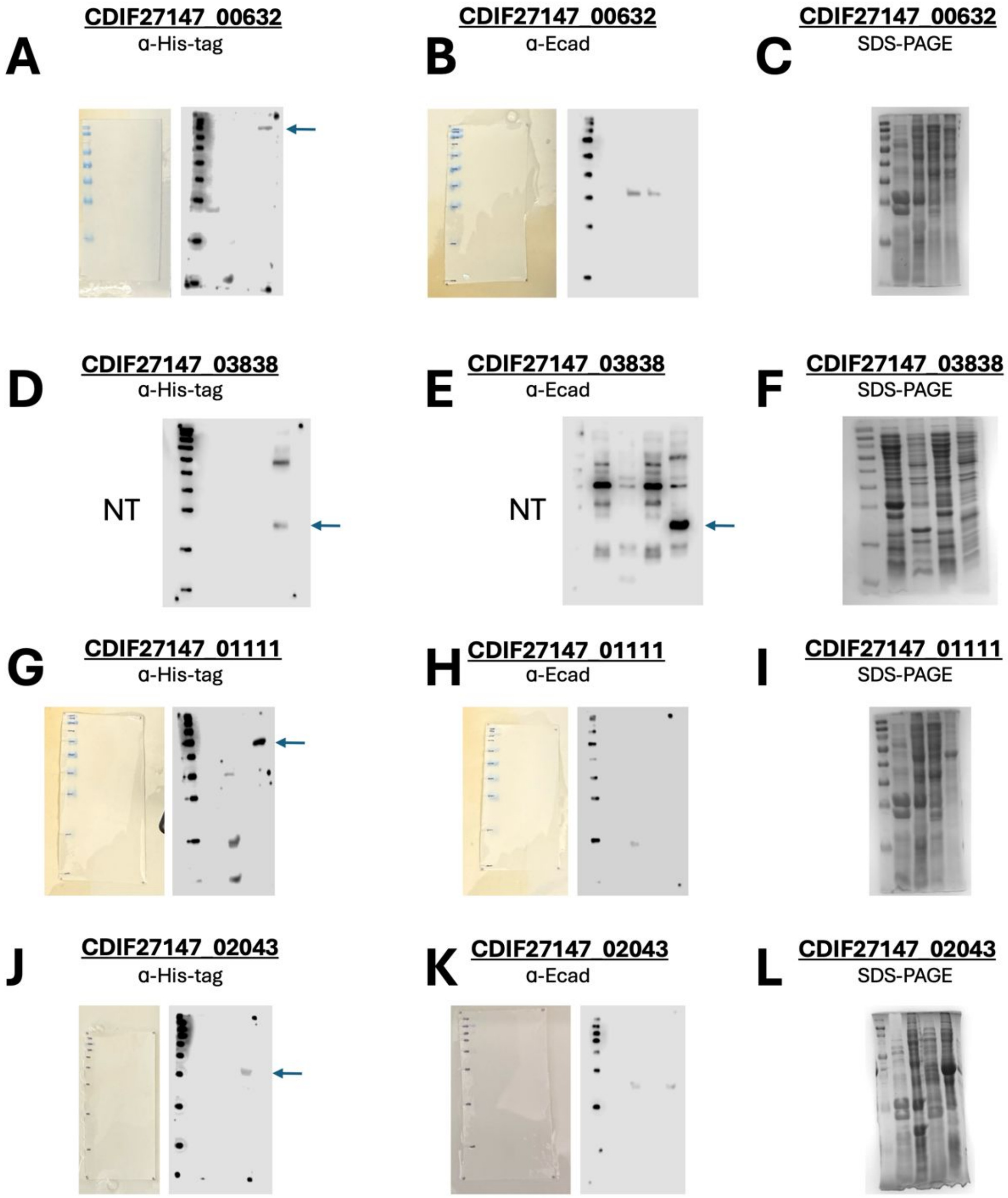

Figure S5 | Raw immunoblot and SDS-PAGE images for CDIF27147_00632, CDIF27147_03838, CDIF27147_01111, and CDIF27147_02043 binding assays shown in Figure 4. (A-C) Raw anti-His-tag immunoblot (A), anti-E-cadherin immunoblot (B), and SDS-PAGE gel (C) for purified His-tagged CDIF27147_00632, with arrows indicating the tagged protein or E-cadherin-bound species. (D-F) Raw anti-His-tag immunoblot (D), anti-E-cadherin immunoblot (E), and SDS-PAGE gel (F) for purified His-tagged CDIF27147_03838. (G-I) Raw anti-His-tag immunoblot (G), anti-E-cadherin immunoblot (H), and SDS-PAGE gel (I) for purified His-tagged CDIF27147_01111. (J-L) Raw anti-His-tag immunoblot (J), anti-E-cadherin immunoblot (K), and SDS-PAGE gel (L) for purified His-tagged CDIF27147_02043. NT indicates that the corresponding nitrocellulose membrane image was not taken. SDS-PAGE gels

(C, F, I, L) show total protein loading and molecular weight marker lanes for the samples in the corresponding immunoblots, underlying the processed data presented in Figure 4.

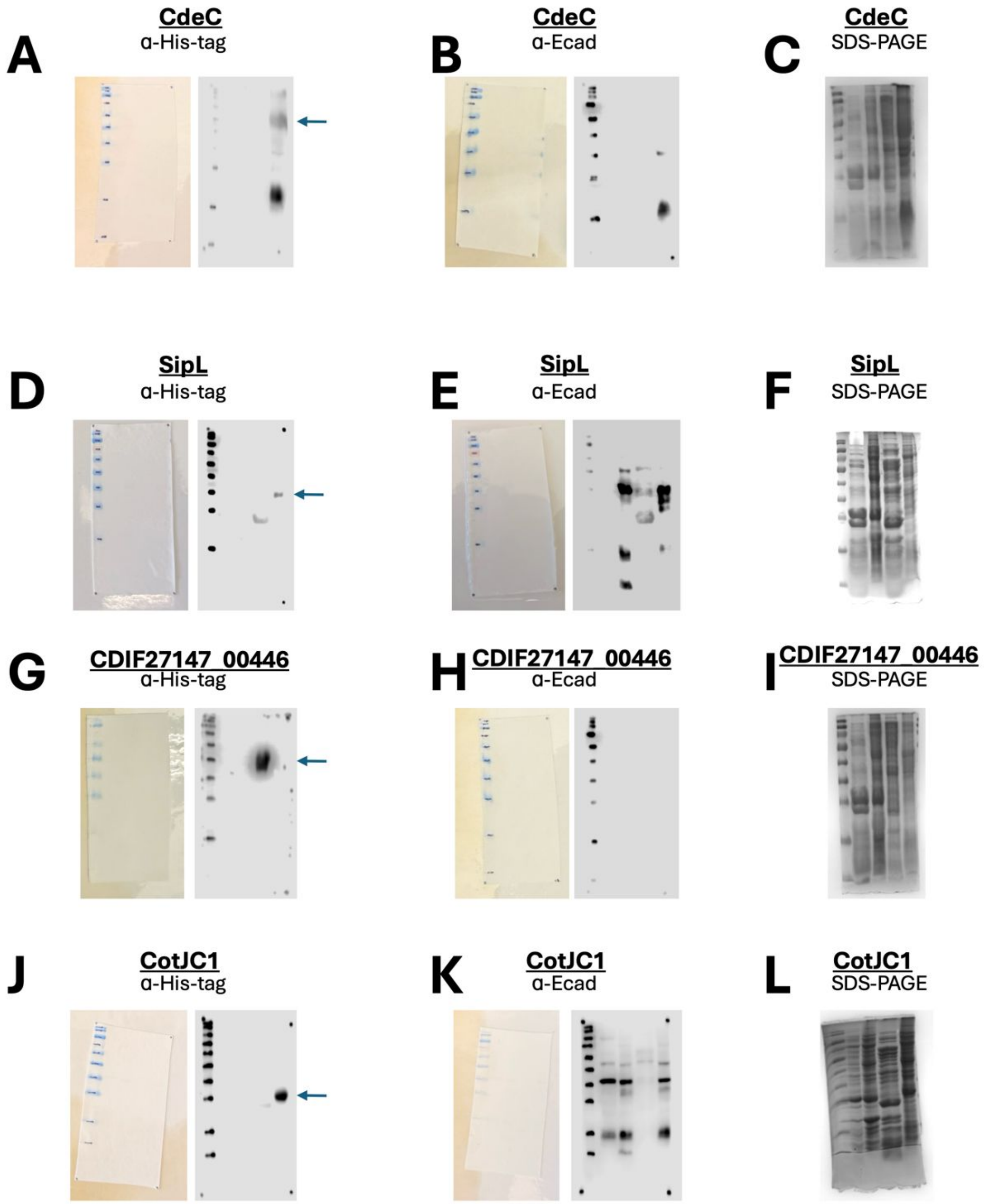

Figure S6 | Raw immunoblot and SDS-PAGE images for CdeC, SipL, CDIF27147_00446, and CotJC1 binding assays shown in Figure 4. (A-C) Raw chemiluminescent immunoblots probed with anti-His-tag antibody (A) or anti-E-cadherin antibody (B), and corresponding SDS-PAGE gel (C) for purified His-tagged CdeC, with arrows indicating the CdeC band or E-cadherin-bound species. (D-F) Raw anti-His-tag immunoblot (D), anti-E-cadherin immunoblot (E), and SDS-PAGE gel (F) for purified His-tagged SipL. (G-I) Raw anti-His-tag immunoblot (G), anti-E-cadherin immunoblot (H), and SDS-PAGE gel (I) for purified His-tagged CDIF27147_00446. (J-L) Raw anti-His-tag immunoblot (J), anti-E-cadherin immunoblot (K),

and SDS-PAGE gel (L) for purified His-tagged CotJC1. NT indicates that the corresponding nitrocellulose membrane image was not taken. SDS-PAGE gels (C, F, I, L) show total protein loading and molecular weight marker lanes for the samples in the corresponding immunoblots, underlying the processed data presented in Figure 4.

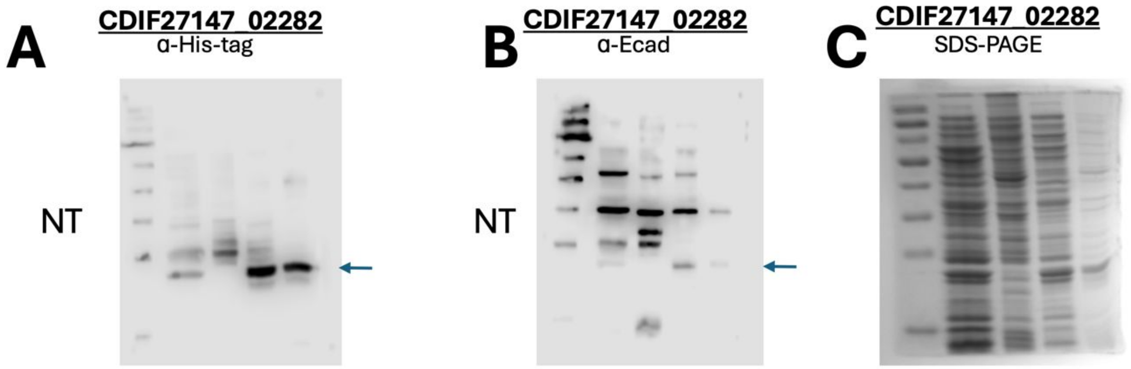

Figure S7 | Raw immunoblot and SDS-PAGE images for CDIF27147_02282 binding assays shown in Figure 4. (A) Raw chemiluminescent immunoblot of purified His-tagged CDIF27147_02282 probed with anti-His-tag antibody (α-His-tag); the arrow indicates the CDIF27147_02282 band. (B) Raw chemiluminescent immunoblot of the same samples probed with anti-E-cadherin antibody (α-E-cad); the arrow indicates E-cadherin binding to CDIF27147_02282. NT indicates that the corresponding nitrocellulose membrane image was not taken. (C) Corresponding Coomassie-stained SDS-PAGE gel showing total protein loading and molecular weight marker lanes for the samples in panels A and B, underlying the processed data shown in Figure 4.

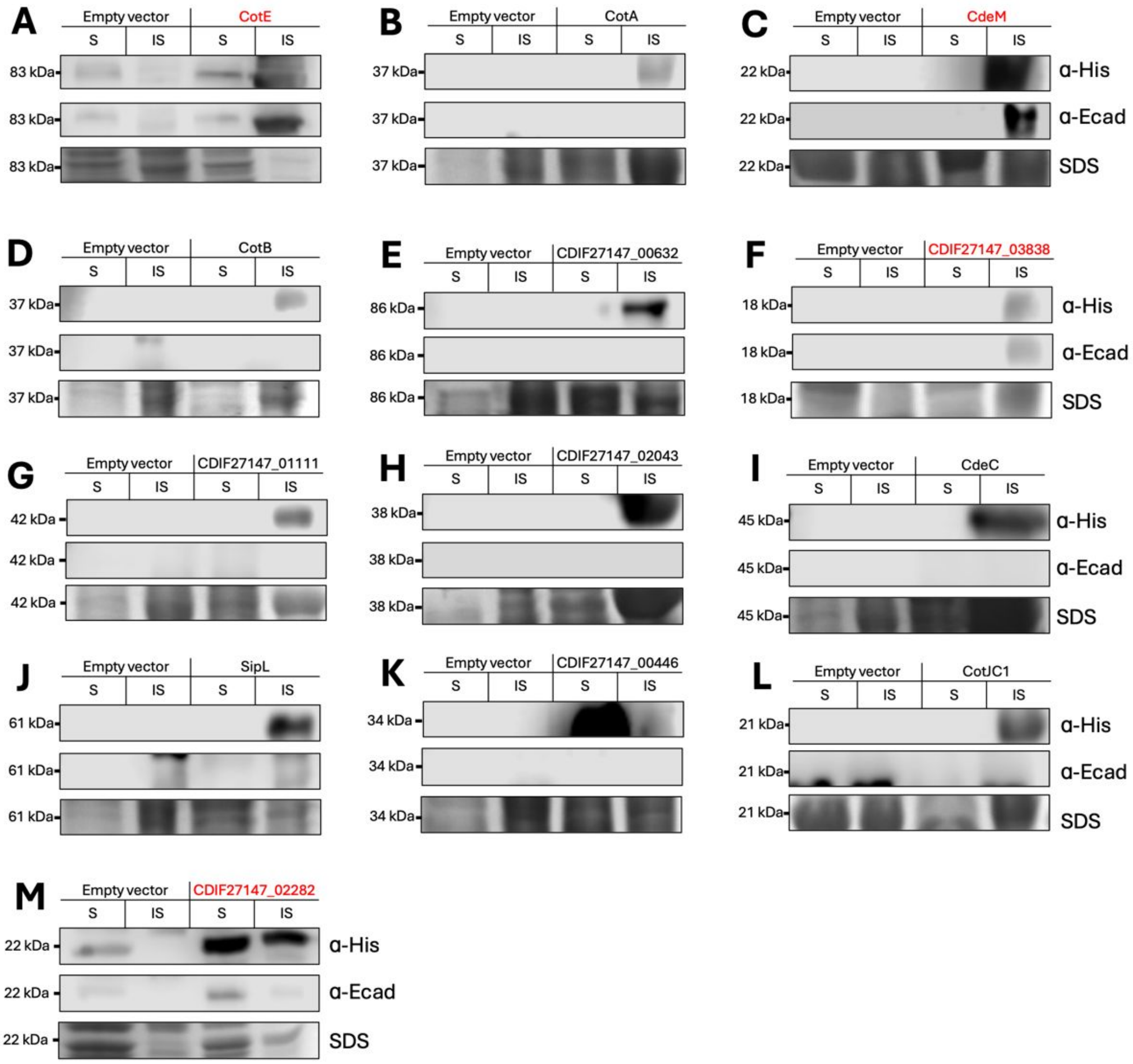

Figure S8 | Replicate analysis of E-cadherin interaction with candidate *C. difficile* R20291$_{CM196}$ spore surface proteins by Far-Western blotting. (A-M) Representative images from a second biological replicate showing the soluble (S) and insoluble (IS) fractions of overexpressed candidate proteins and empty vector control separated by 12% SDS-PAGE and transferred to nitrocellulose membranes. Membranes were probed sequentially with anti-His antibody (α-His) to verify protein expression, human recombinant E-cadherin followed by anti-E-cadherin antibody (α-Ecad) to detect E-cadherin binding, and a gel image for loading control. Experimental procedures matched those described for the primary Far-Western analyses (Figure 4). Molecular weight markers are indicated in kDa. Red protein names correspond to proteins that showed binding to E-cadherin *in vitro*.

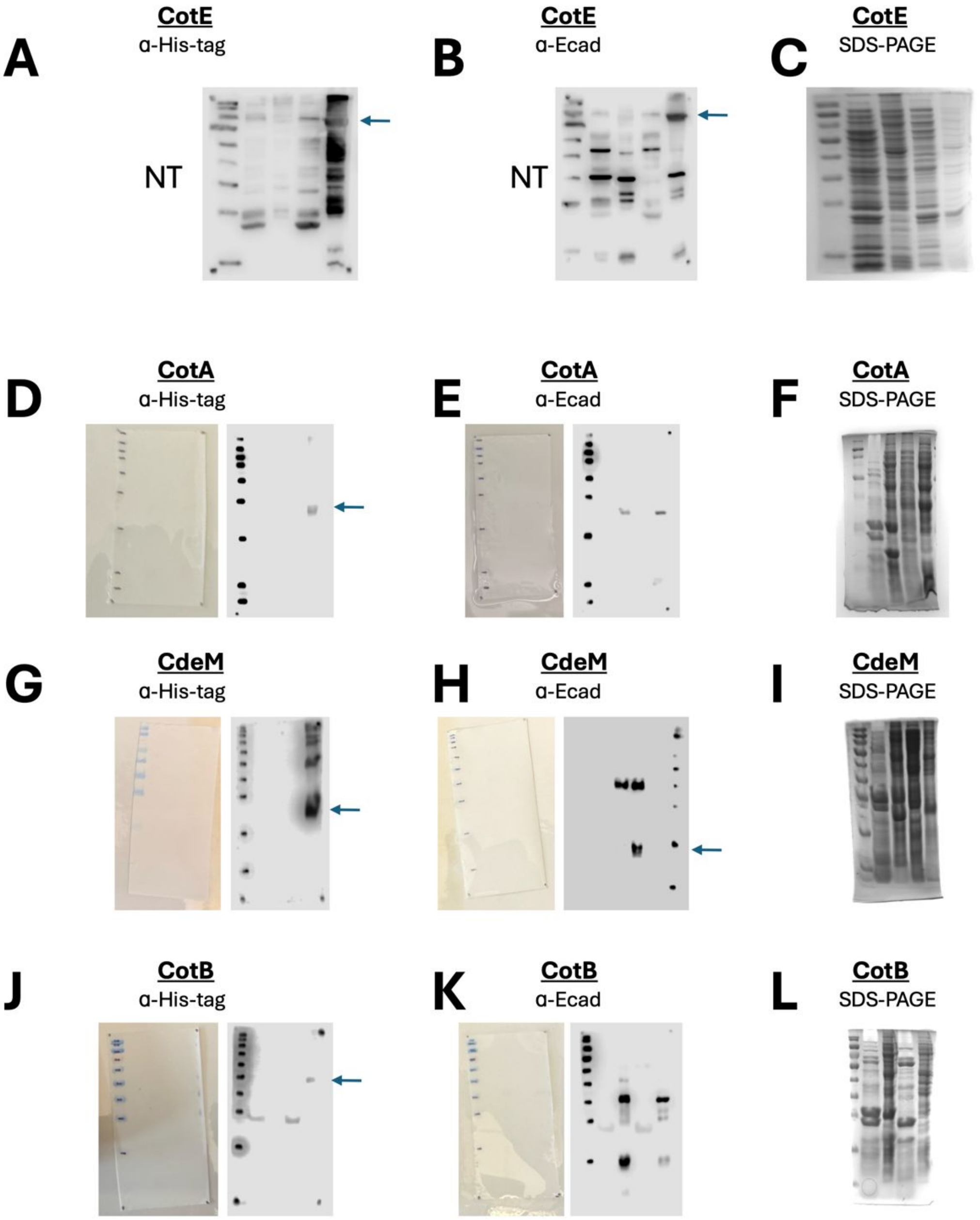

Figure S9 | Raw immunoblot and SDS-PAGE images for CotE, CotA, CdeM, and CotB binding assays shown in Figure S8. (A-C) Raw anti-His-tag immunoblot (A), anti-E-cadherin immunoblot (B), and SDS-PAGE gel (C) for purified His-tagged CotE, with arrows indicating the CotE band or E-cadherin-bound species. (D-F) Raw anti-His-tag immunoblot (D), anti-E-cadherin immunoblot (E), and SDS-PAGE gel (F) for purified His-tagged CotA. (G-I) Raw anti-His-tag immunoblot (G), anti-E-cadherin immunoblot (H), and SDS-PAGE gel (I) for purified His-tagged CdeM. (J-L) Raw anti-His-tag immunoblot (J), anti-E-cadherin immunoblot (K), and SDS-PAGE gel (L) for purified His-tagged CotB. SDS-PAGE gels (C, F, I, L) show total protein loading and molecular weight marker lanes for the samples in the corresponding immunoblots. NT indicates that the corresponding nitrocellulose membrane image was not taken.

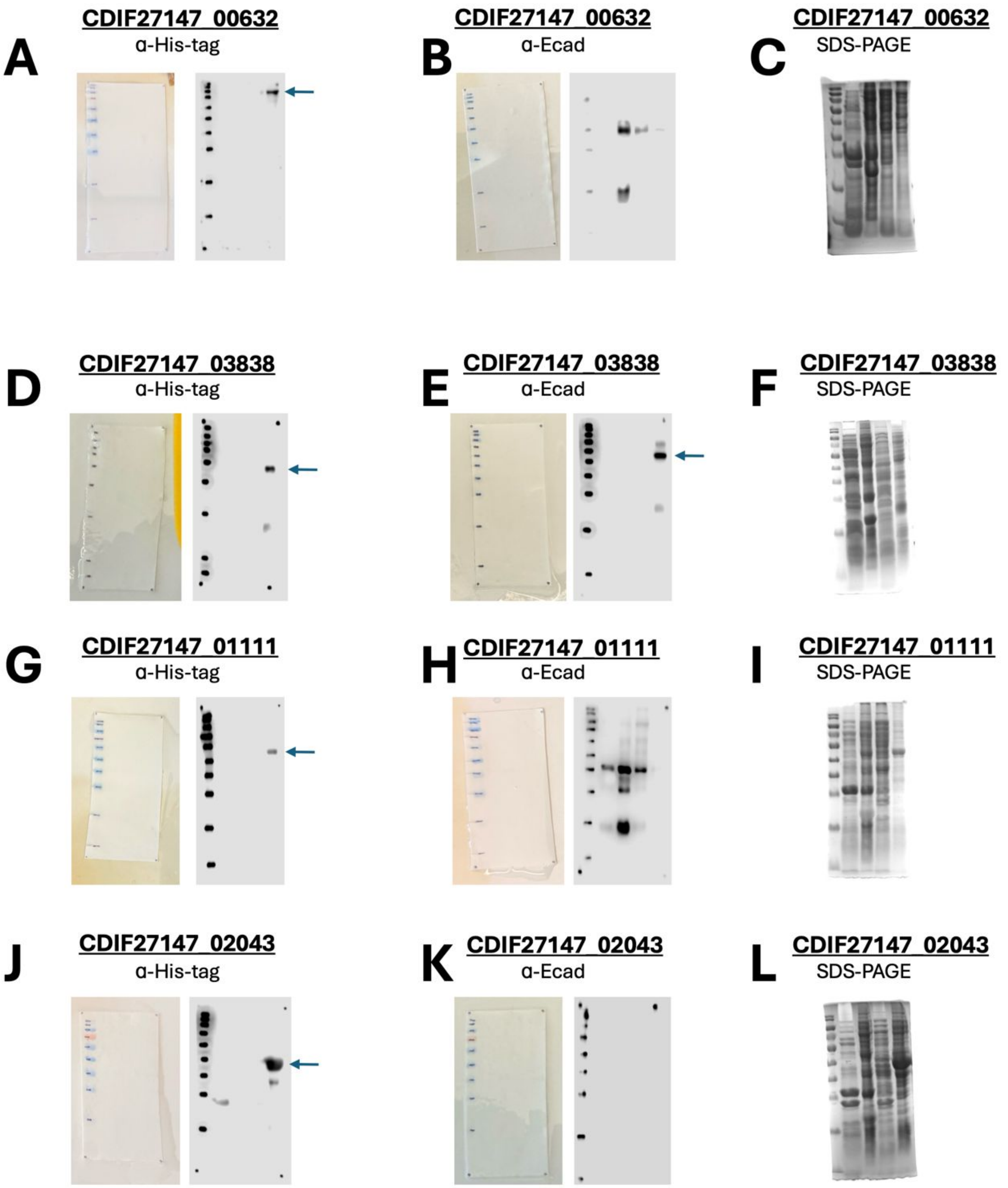

Figure S10 | Raw immunoblot and SDS-PAGE images for CDIF27147_00632, CDIF27147_03838, CDIF27147_01111, and CDIF27147_02043 binding assays shown in Figure S8. (A-C) Raw anti-His-tag immunoblot (A), anti-E-cadherin immunoblot (B), and SDS - PAGE gel (C) for purified His-tagged CDIF27147_00632, with arrows indicating the tagged protein or E-cadherin-bound species. (D-F) Raw anti-His-tag immunoblot (D), anti-E-cadherin immunoblot (E), and SDS-PAGE gel (F) for purified His-tagged CDIF27147_03838. (G-I) Raw anti-His-tag immunoblot (G), anti-E-cadherin immunoblot (H), and SDS-PAGE gel (I) for purified His-tagged CDIF27147_01111. (J-L) Raw anti-His-tag immunoblot (J), anti-E-cadherin immunoblot (K), and SDS-PAGE gel (L) for purified His-tagged CDIF27147_02043. SDS-

PAGE gels (C, F, I, L) show total protein loading and molecular weight marker lanes for the samples in the corresponding immunoblots.

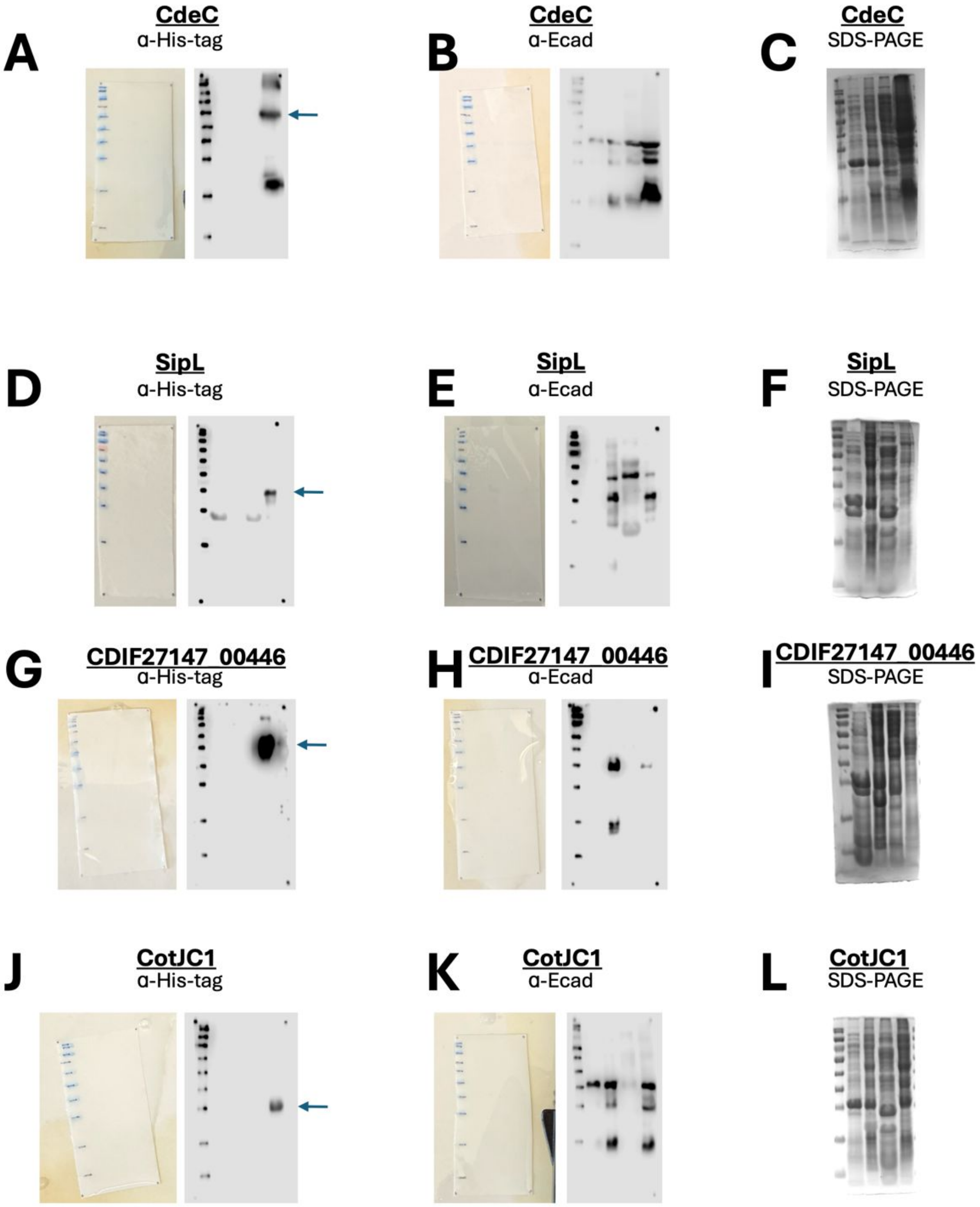

Figure S11 | Raw immunoblot and SDS-PAGE images for CdeC, SipL, CDIF27147_00446, and CotJC1 binding assays shown in Figure S8. (A-C) Raw anti-His-tag immunoblot (A), anti-E-cadherin immunoblot (B), and SDS-PAGE gel (C) for purified His-tagged CdeC, corresponding to the processed data in Figure S8 for this construct; arrows indicate the CdeC band or E-cadherin-bound species. (D-F) Raw anti-His-tag immunoblot (D), anti-E-cadherin immunoblot (E), and SDS-PAGE gel (F) for purified His-tagged SipL. (G-I) Raw anti-His-tag immunoblot (G), anti-E-cadherin immunoblot (H), and SDS-PAGE gel (I) for purified His-tagged CDIF27147_00446. (J-L) Raw anti-His-tag immunoblot (J), anti-E-cadherin

immunoblot (K), and SDS-PAGE gel (L) for purified His-tagged CotJC1. SDS-PAGE gels (C, F, I, L) show total protein loading and molecular weight marker lanes for the samples in the corresponding immunoblots.

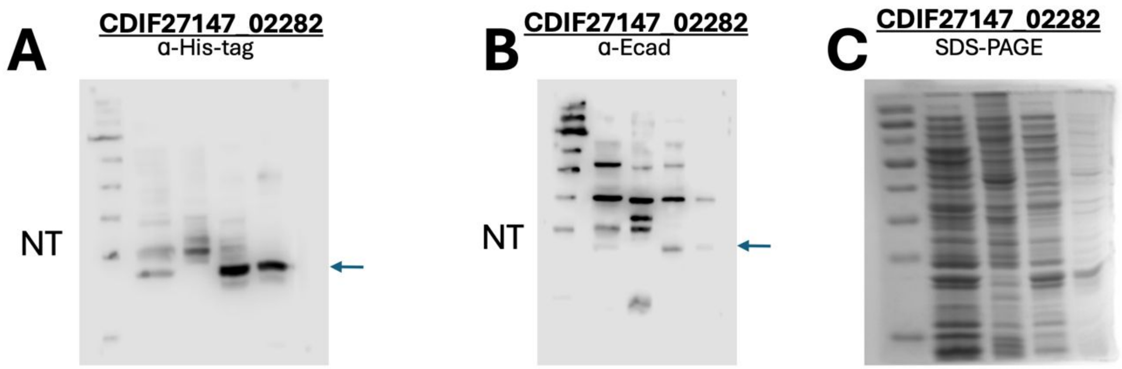

Figure S12 | Raw immunoblot and SDS-PAGE images for CDIF27147_02282 binding assays shown in Figure S8. (A) Raw anti-His-tag immunoblot of purified His-tagged CDIF27147_02282, with the arrow indicating the CDIF27147_02282 band. (B) Raw anti-E-cadherin immunoblot of the same samples, with the arrow indicating E-cadherin binding to CDIF27147_02282. (C) Corresponding Coomassie-stained SDS-PAGE gel showing total protein loading and molecular weight marker lanes for the samples in panels A and B. NT indicates that the corresponding nitrocellulose membrane image was not taken.

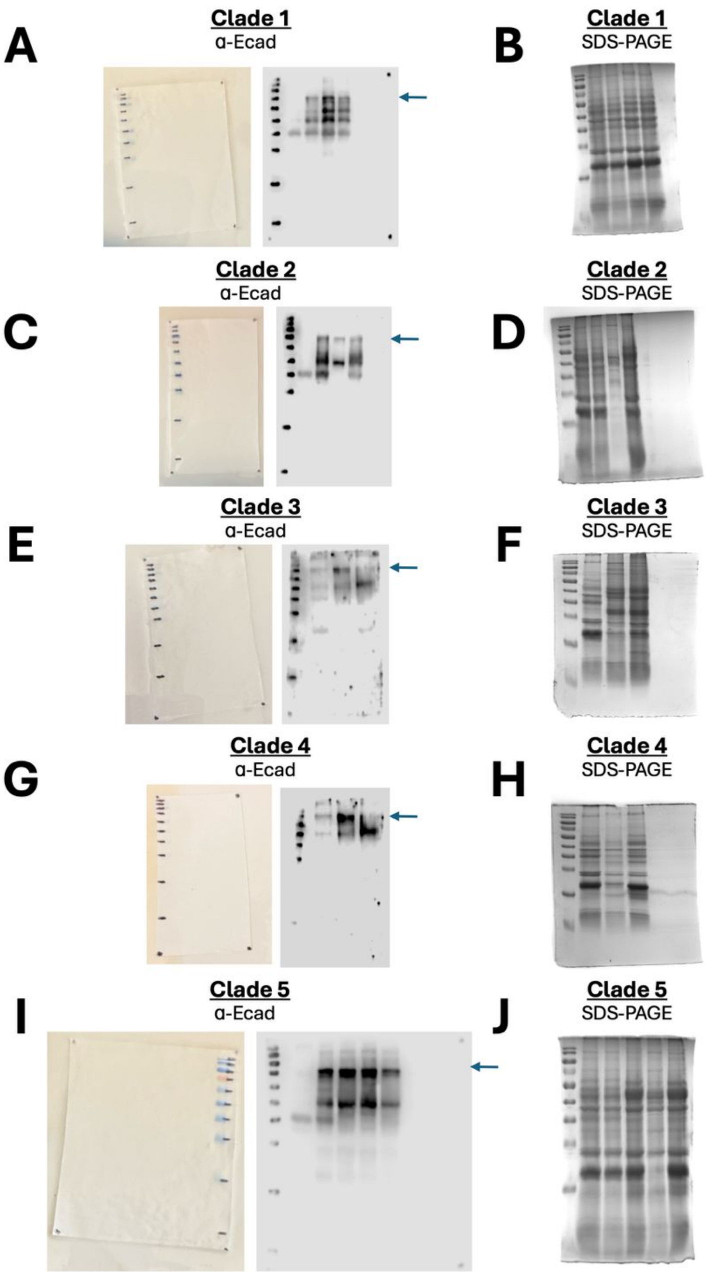

Figure S13 | Raw immunoblot and SDS-PAGE images for E-cadherin pull-down assays with spores from *C. difficile* Clades 1-5 shown in Figure 6. (A,B) Raw anti-E-cadherin immunoblot (A) and corresponding SDS-PAGE gel (B) for coat and exosporium extracts from *C. difficile* Clade 1 spores incubated with recombinant human E-cadherin; arrows indicate E-cadherin-reactive bands. (C,D) Raw anti-E-cadherin immunoblot (C) and SDS-PAGE gel (D) for Clade 2 spore extracts. (E,F) Raw anti-E-cadherin immunoblot (E) and SDS-PAGE gel (F) for Clade 3 spore extracts. (G,H) Raw anti-E-cadherin immunoblot (G) and SDS-PAGE gel (H) for Clade 4 spore extracts. (I,J) Raw anti-E-cadherin immunoblot (I) and SDS-PAGE gel (J) for Clade 5 spore extracts. SDS-PAGE gels (B, D, F, H, J) show total protein loading and molecular weight marker lanes for the samples in the corresponding immunoblots, underlying the processed data presented in Figure 6.

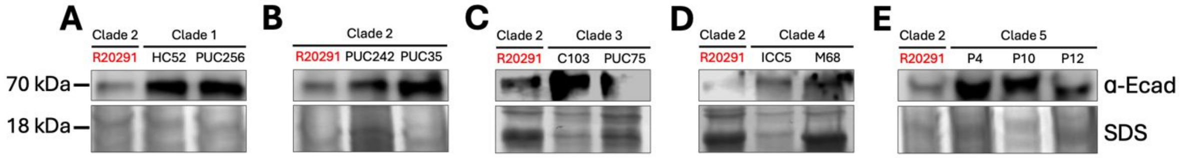

Figure S14 | Replicate analysis of E-cadherin binding by spore coat/exosporium extracts from diverse *C. difficile* Clade strains. (A-E) Far-Western blots showing binding of 1 µg/mL recombinant human E-cadherin to spore coat/exosporium protein extracts from *C. difficile* strains representing Clades 1-5. Membranes were probed with anti-E-cadherin antibody (top) and SDS-PAGE images are shown below as loading controls.

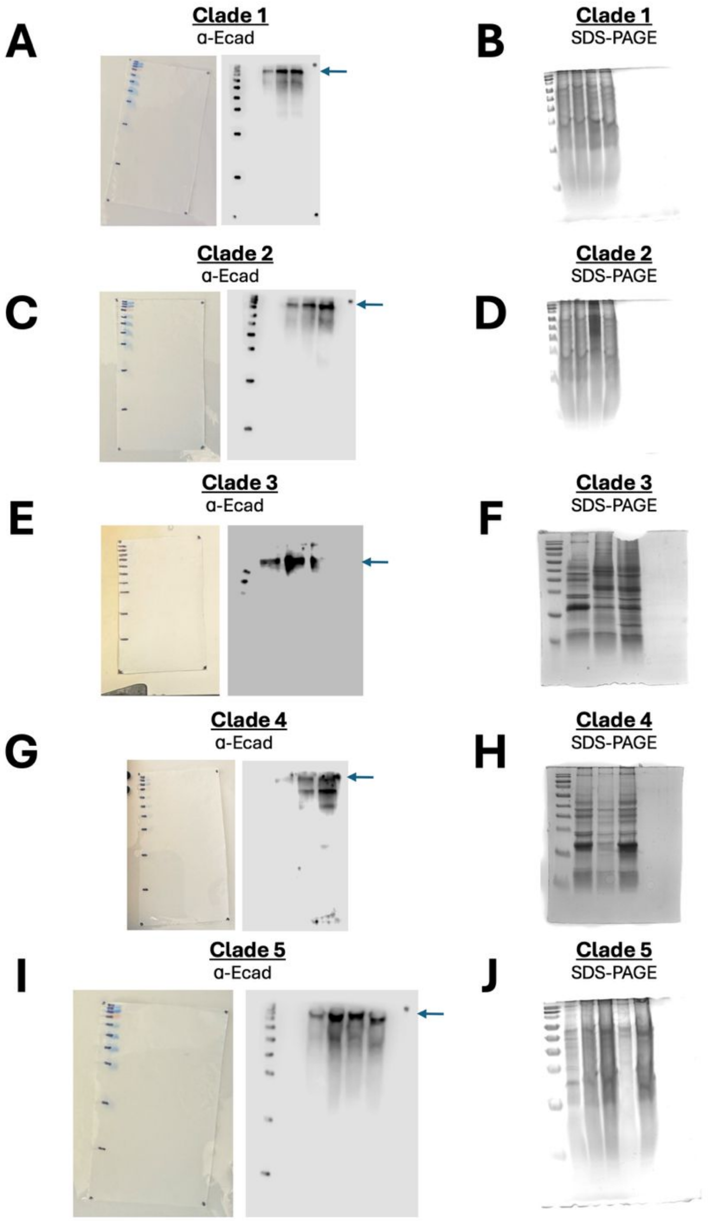

Figure S15 | Raw immunoblot and SDS-PAGE images for replicate E-cadherin binding assays with spore coat/exosporium extracts from *C. difficile* Clade strains shown in Figure S14. (A,B) Raw anti-E-cadherin immunoblot (A) and corresponding SDS-PAGE gel (B) for spore coat/exosporium protein extracts from *C. difficile* Clade 1 strains incubated with recombinant human E-cadherin; arrows indicate E-cadherin-reactive bands. (C,D) Raw anti-E-cadherin immunoblot (C) and SDS-PAGE gel (D) for Clade 2 extracts. (E,F) Raw anti-E-cadherin immunoblot (E) and SDS-PAGE gel (F) for Clade 3 extracts. (G,H) Raw anti-E-cadherin immunoblot (G) and SDS-PAGE gel (H) for Clade 4 extracts. (I,J) Raw anti-E-cadherin immunoblot (I) and SDS-PAGE gel (J) for Clade 5 extracts. SDS-PAGE gels (B, D, F, H, J) show total protein loading and molecular weight marker lanes for the samples in the corresponding immunoblots, underlying the processed replicate data presented in Figure S14.

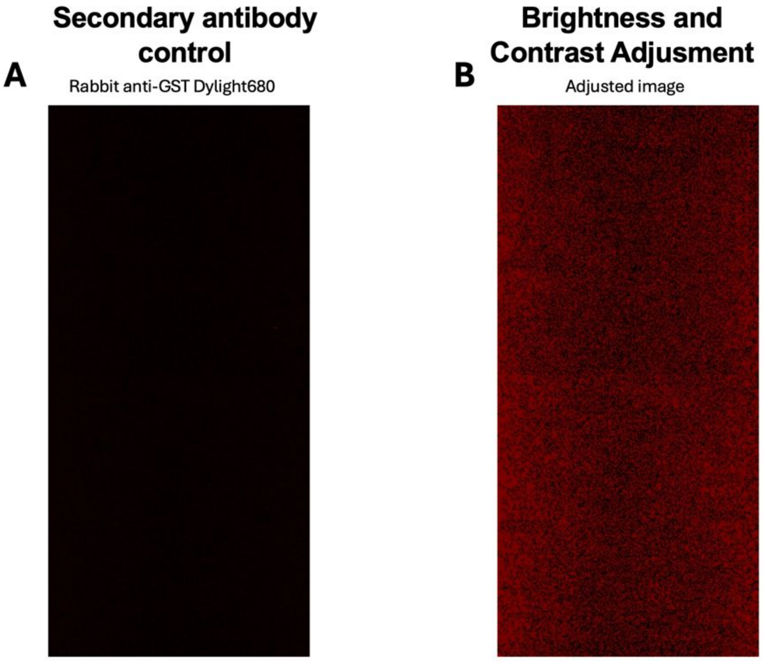

Figure S16 | Secondary antibody only control on conformational peptide microarray. (A) Rabbit anti GST DyLight 680 secondary antibody was incubated on a microarray containing 2,972 cyclic constrained peptides to assess nonspecific binding prior to primary antibody assays. (B) Brightness and contrast adjusted view of the same scan shows uniform background without discrete peptide features, indicating no detectable secondary antibody interactions. Therefore, quantification with PepSlide Analyzer was not performed for this control.

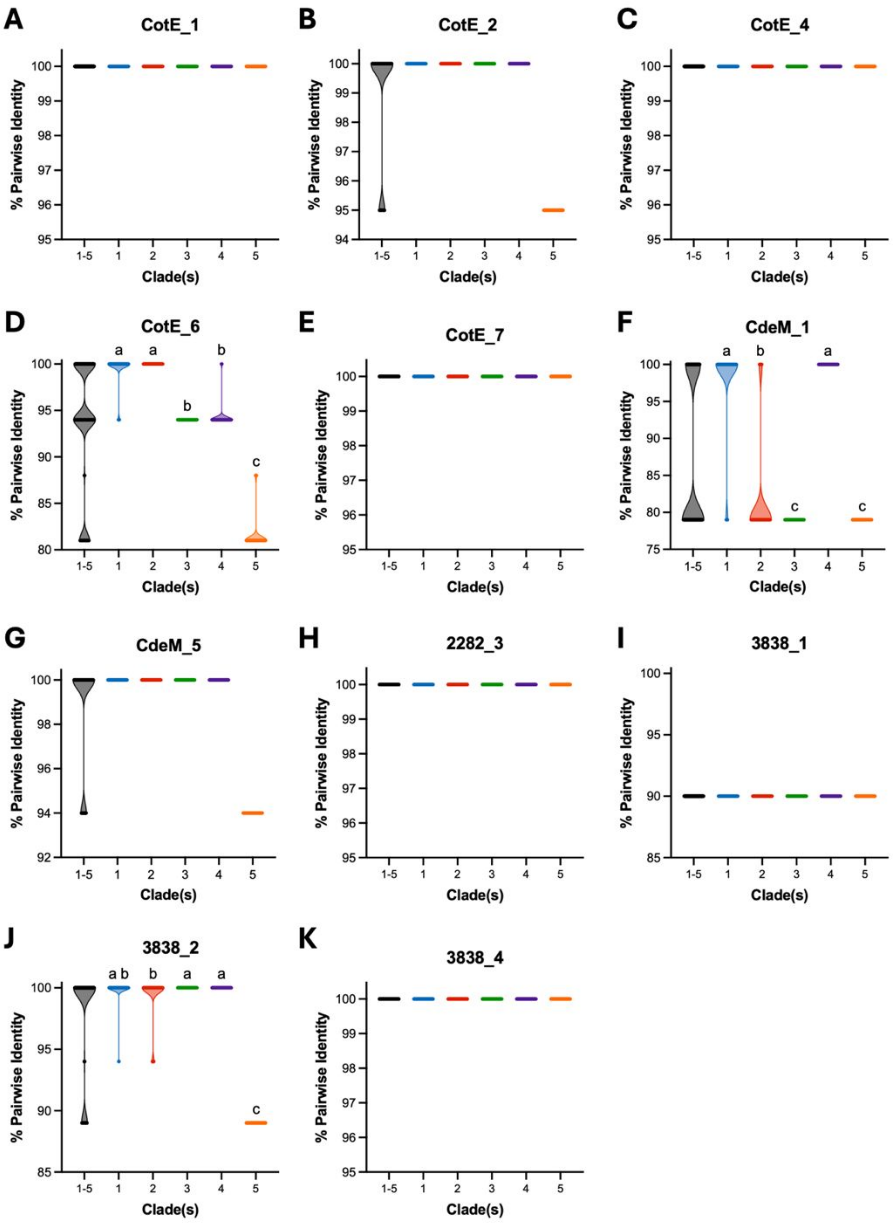

Figure S17 | Conservation of candidate E-cadherin-binding motifs across *C. difficile* Clades. (A-K) Violin plot distributions of pairwise amino acid identity for motif sequences from (A) CotE_1, (B) CotE_2, (C) CotE_4, (D) CotE_6, (E) CotE_7, (F) CdeM_1, (G) CdeM_5, (H) 2282_3, (I) 3838_1, (J) 3838_2, and (K) 3838_4 among 50 genomes from each of the five major *C. difficile* Clades. For each motif, amino acid sequences from all sequences were compared against the R20291 reference sequence. The percent pairwise identity for each genome was extracted and plotted by clade to assess intra- and inter-clade sequence conservation profiles. Statistical significance was determined by ordinary one-way ANOVA with Šídák's multiple comparisons test. Groups not sharing a letter are significantly different ($P < 0.05$) as determined by multiple comparison test; groups sharing one or more letters are not significantly different. Panels without statistical letters indicate no variation within or between clades (statistical error of zero), significance cannot be calculated.

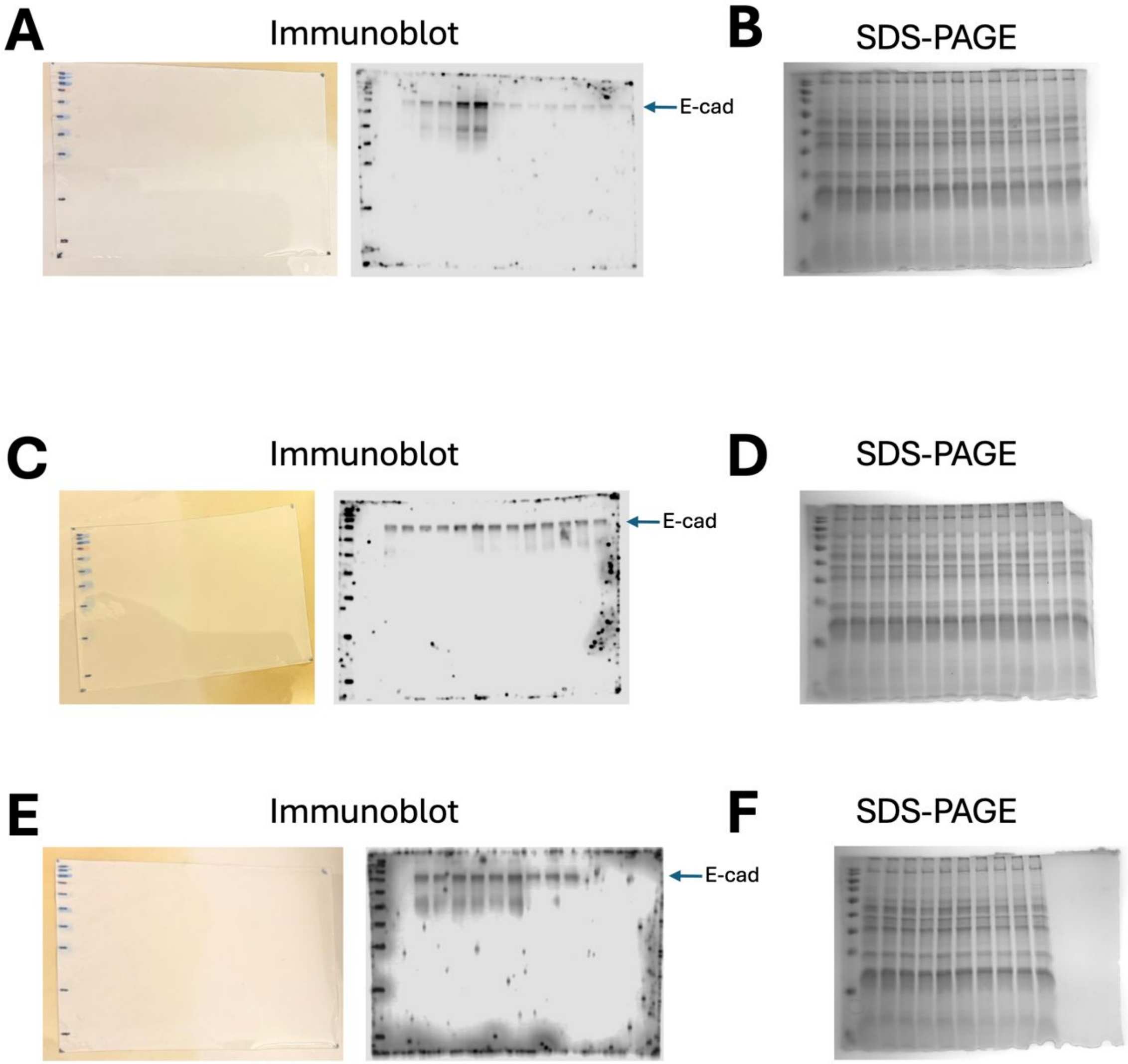

Figure S18 | Raw immunoblot and SDS-PAGE images for peptide inhibition assays shown in Figure 9 (first set of constructs). (A,B) Raw chemiluminescent immunoblot (A) and corresponding SDS-PAGE gel (B) for Far-Western analysis of E-cadherin binding to *C. difficile* $R20291_{CM196}$ spores incubated with increasing concentrations of the indicated synthetic peptide and scramble control, as shown in Figure 9A. The immunoblot was probed with anti-E-cadherin antibody (α-E-cad), and the arrow indicates the E-cadherin band. (C,D) Raw chemiluminescent immunoblot (C) and SDS-PAGE gel (D) corresponding to the peptide inhibition experiment shown in Figure 9B. (E,F) Raw chemiluminescent immunoblot (E) and SDS-PAGE gel (F) corresponding to the peptide inhibition experiment shown in Figure 9C. SDS-PAGE gels (B, D, F) show total protein loading and molecular weight marker lanes for the samples in the respective immunoblots.

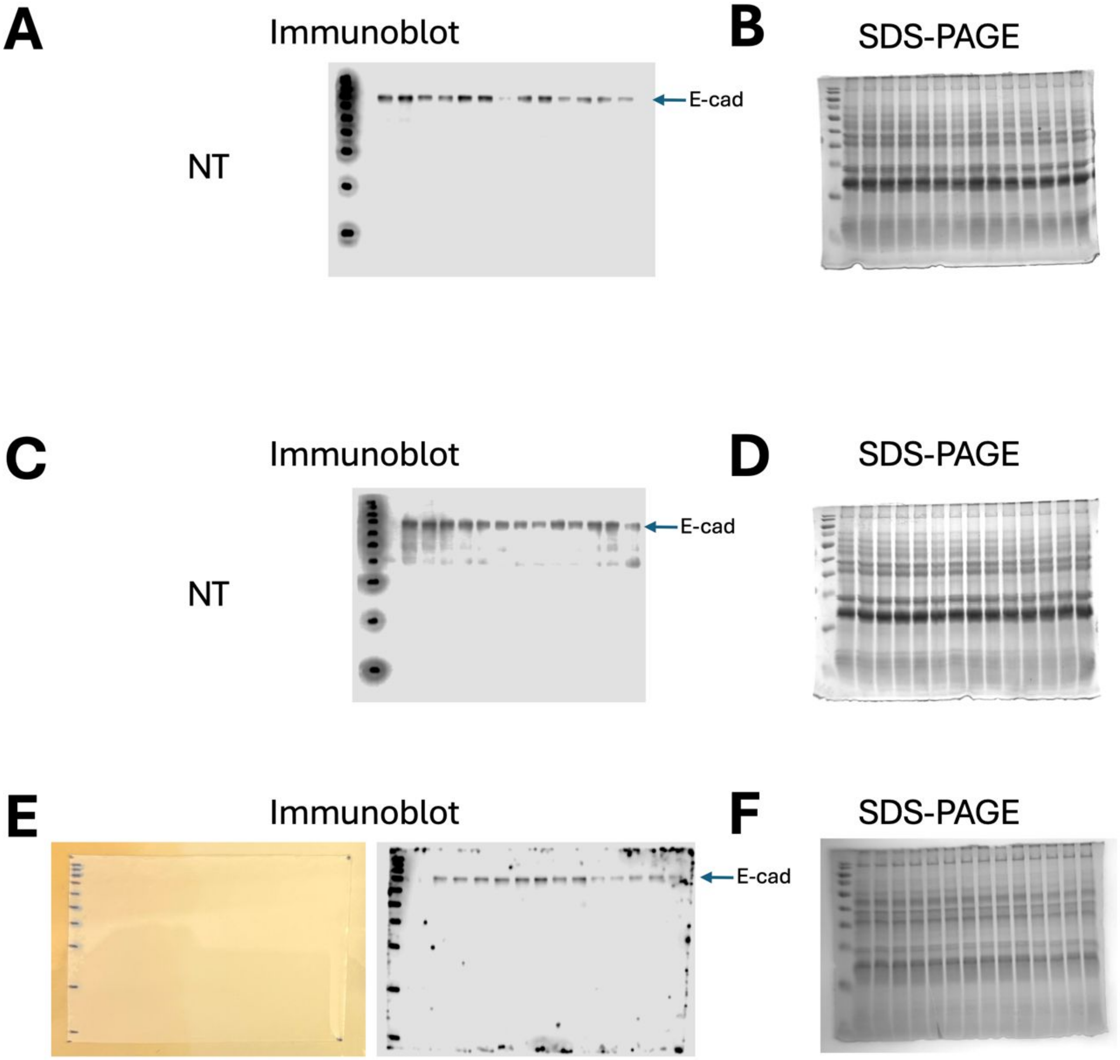

Figure S19 | Raw immunoblot and SDS-PAGE images for peptide inhibition assays shown in Figure 9 (second set of constructs). (A,B) Raw chemiluminescent immunoblot (A) and corresponding SDS-PAGE gel (B) for Far-Western analysis of E-cadherin binding to $R20291_{CM196}$ spores incubated with increasing concentrations of the synthetic peptide and scramble control used in Figure 9D. (C,D) Raw chemiluminescent immunoblot (C) and SDS-PAGE gel (D) corresponding to the peptide inhibition experiment shown in Figure 9E. (E,F) Raw chemiluminescent immunoblot (E) and SDS-PAGE gel (F) corresponding to the peptide inhibition experiment shown in Figure 9F. Immunoblots were probed with anti-E-cadherin antibody (α-E-cad), and arrows indicate the E-cadherin band. SDS-PAGE gels (B, D, F) show total protein loading and molecular weight marker lanes for the samples in the respective immunoblots. NT indicates that the corresponding nitrocellulose membrane image was not taken.

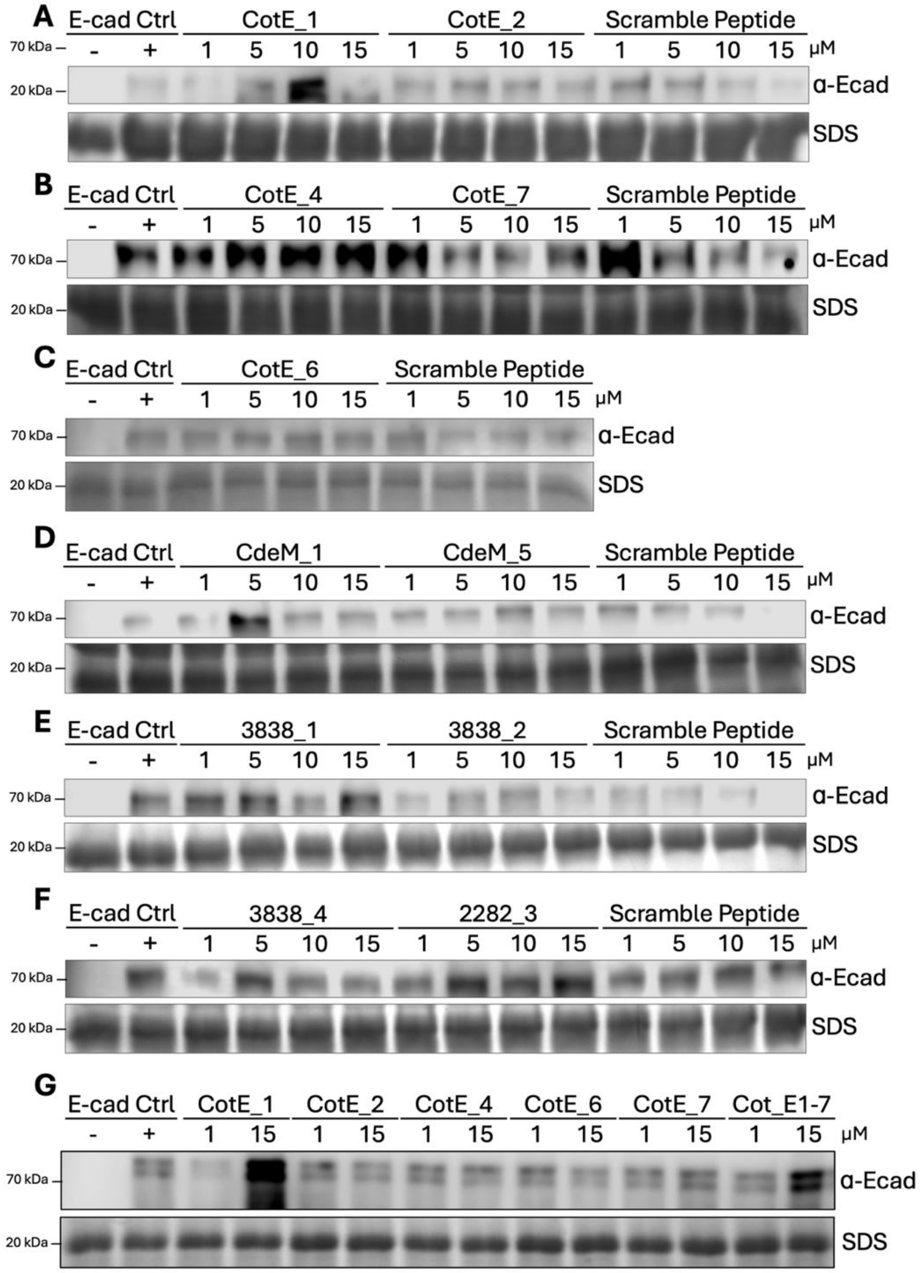

Figure S20 | Replicate gels of E-cadherin pull-down assays to evaluate effect of synthetic peptides on binding to *C. difficile* R20291$_{CM196}$ spores. (A-F) Far-Western blots from independent replicates of the inhibition experiments. R20291$_{CM196}$ spores were incubated with E-cadherin in the presence of increasing concentrations (1, 5, 10, 15 μM) of synthetic peptides representing predicted binding motifs from CotE, CdeM, CDIF27147_03838, and CDIF27147_02282, or control scramble peptide. Detection was performed with anti-E-cadherin antibody, and SDS-PAGE images below serve as loading controls. (G) Far-Western blot from inhibition experiments. R20291$_{CM196}$ spores were incubated with E-cadherin in the presence of 1 or 15 μM of synthetic peptides from representing predicted binding motifs from CotE, or with a combination of all CotE-derived peptides at 1 μM or 15 μM each. Detection was performed with anti-E-cadherin antibody, and SDS-PAGE images below serve as loading controls.

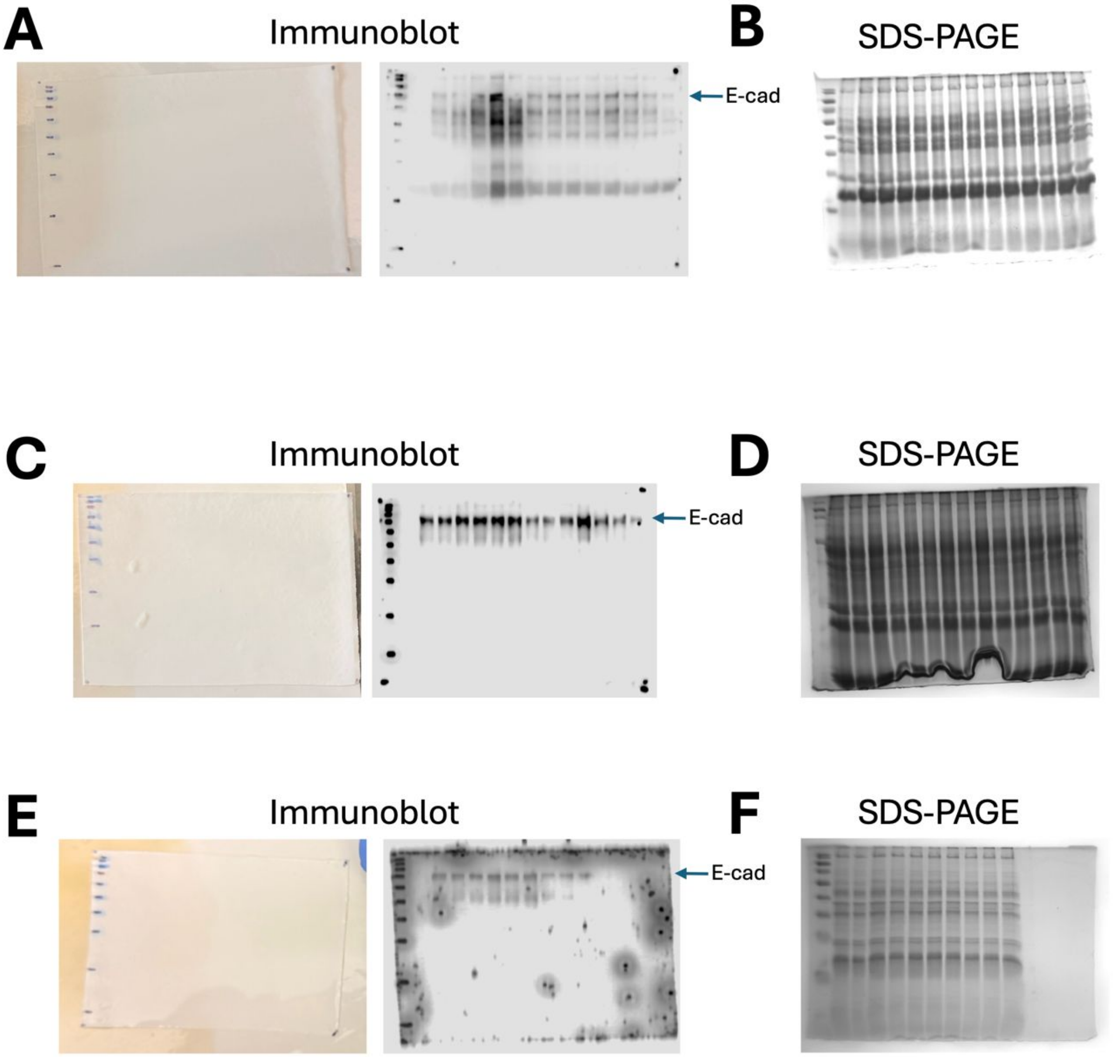

Figure S21 | Raw immunoblot and SDS-PAGE images for replicate E-cadherin pull-down assays with synthetic peptides shown in Figure S20 (first set of constructs). (A,B) Raw chemiluminescent immunoblot (A) and corresponding SDS-PAGE gel (B) for Far-Western analysis of E-cadherin binding to *C. difficile* $R20291_{CM196}$ spores incubated with increasing concentrations of CotE-derived peptides CotE_1 and CotE_2 and scramble peptide, corresponding to the experiment shown in Figure S20A. (C,D) Raw chemiluminescent immunoblot (C) and SDS-PAGE gel (D) for replicate inhibition assays with CotE_4 and CotE_7 peptides and scramble peptide, corresponding to Figure S20B. (E,F) Raw chemiluminescent immunoblot (E) and SDS-PAGE gel (F) for replicate inhibition assays with CotE_6 peptide and scramble peptide, corresponding to Figure S20C. Immunoblots were probed with anti-E-cadherin antibody (α-E-cad), and arrows indicate the E-cadherin band. SDS-PAGE gels (B, D, F) show total protein loading and molecular weight marker lanes for the samples in the respective immunoblots.

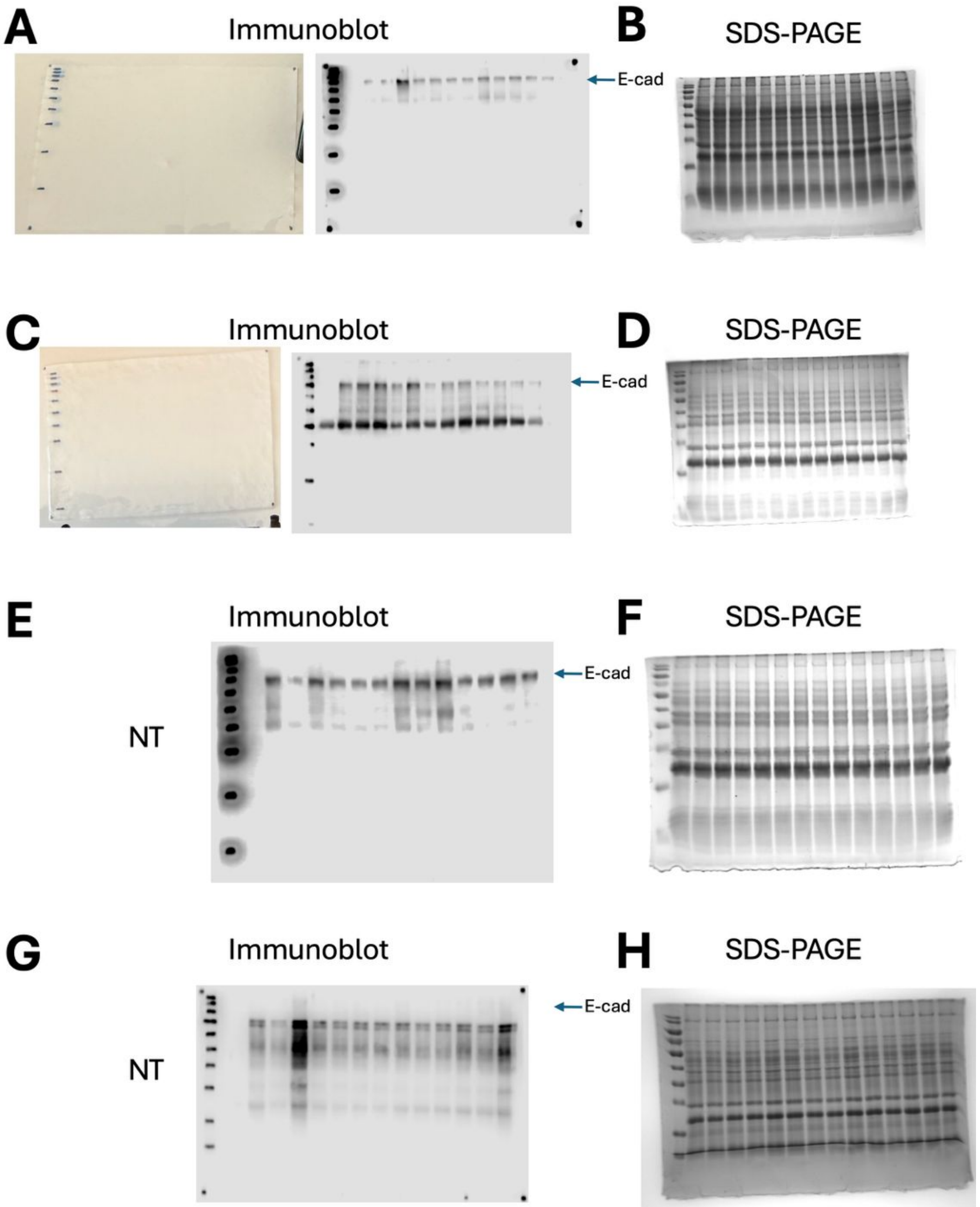

Figure S22 | Raw immunoblot and SDS-PAGE images for replicate E-cadherin pull-down assays with synthetic peptides shown in Figure S20 (second set of constructs). (A,B) Raw chemiluminescent immunoblot (A) and corresponding SDS-PAGE gel (B) for Far-Western analysis of E-cadherin binding to R20291$_{CM196}$ spores incubated with CdeM-derived peptides CdeM_1 and CdeM_5 and scramble peptide, corresponding to Figure S20D. (C,D) Raw chemiluminescent immunoblot (C) and SDS-PAGE gel (D) for replicate inhibition assays with CDIF27147_03838-derived peptides 3838_1 and 3838_2 and scramble peptide, corresponding to Figure S20E. (E,F) Raw chemiluminescent immunoblot (E) and SDS-PAGE gel (F) for replicate inhibition assays with 3838_4 and 2282_3 peptides and scramble peptide, corresponding to Figure S20F. Immunoblots were probed with anti-E-cadherin antibody (α-E-cad), and arrows indicate the E-cadherin band. SDS-PAGE gels (B, D, F) show total protein loading and

molecular weight marker lanes for the samples in the respective immunoblots. NT indicates that the corresponding nitrocellulose membrane image was not taken.

TABLE S1. Bacterial strains used

Strain	Relevant characteristic	Source/Reference
E. coli NEB Turbo	*F' proA^{+}B^{+} lacI^{q} ΔlacZM15 / fhuA2 Δ(lac-proAB) glnV galK16 galE15 R(zgb-210::Tn10)Tet^{S} endA1 thi-1 Δ(hsdS-mcrB)5*	New England Biolabs
E. coli BL21-CodonPlus(DE3)-RIL	Genome contains extra copies of the *argU*, *ileY*, and *leuW* tRNA genes. These genes encode tRNAs that recognize the arginine codons AGA and AGG, the isoleucine codon AUA, and the leucine codon CUA, respectively	Agilent (previously Stratagene)
C. difficile R20291$_{CM196}$	Hypersporulating strain derivative of R20291 strain (CM196; DMSO 435)	18
C. difficile HC52	Ribotype 014-020, Clade 1 strain	9
C. difficile PUC256	Ribotype 014-020, Clade 1 strain	9
C. difficile PUC242	Ribotype 106, Clade 2 strain	9
C. difficile PUC351	Ribotype 106, Clade 2 strain	9
C. difficile C103	Ribotype 023, Clade 3 strain	9
C. difficile PUC75	Ribotype 023, Clade 3 strain	9
C. difficile ICC5	Ribotype 017, Clade 4 strain	9
C. difficile M68	Ribotype 017, Clade 4 strain	9
C. difficile P4	Ribotype 078, Clade 5 strain	9
C. difficile P10	Ribotype 078, Clade 5 strain	9
C. difficile P12	Ribotype 078, Clade 5 strain	9

Table S2. Primers used

Primer code/Name	Primer sequence[a]	Position[b]	Gene	Use[c]
P1493 FP *cotE* pet16b NdeI	*ATATCGAAGGTCGTCAT*ATGCCAAATTTGCCAAGTTTAGGGTC	+1 to +26	*cotE*	OE
P1492 RP *cotE* pet16b BamHI	*CTTTGTTAGCAGCCGGATCC*TTAGAATTGCCCATAAATACCTTCAAGTTC	+2100 to +2130	*cotE*	OE
P1497 FP *cotA* pet16b NdeI	*ATATCGAAGGTCGTCAT*GTGGAAAATAATAAATGTAGAGAGGACTTTAG	+1 to +32	*cotA*	OE
P1496 RP *cotA* pet16b BamHI	*CTTTGTTAGCAGCCGGATCC*TTATGCAATATAATCTATAGAATCTACACATACAAAG	+882 to +918	*cotA*	OE
P1487 FP *cdeM* pet16b NdeI	*ATATCGAAGGTCGTCAT*ATGGAAAATAAAAAATATGCAAATGGTGGTTATTC	+1 to +35	*cdeM*	OE
P1486 RP *cdeM* pet16b BamHI	*CTTTGTTAGCAGCCGGATCC*TTATTTTCTACAGCAGTTACAATTACATTTATGG	+462 to +495	*cdeM*	OE
P1495 FP *cotB* pet16b NdeI	*ATATCGAAGGTCGTCAT*ATGATAGATAATCAAAAATATGTTATTTTATCACTAGAATTAC	+1 to +43	*cotB*	OE

P1494 RP *cotB* pet16b BamHI	*CTTTGTTAGCAGCCGGATCC*TTACATGTTTTTATAACTCTCCAATATTCTAATATAATG	+877 to +915	*cotB*	OE
P1507 FP CDR20291_0489 pet16b NdeI	*ATATCGAAGGTCGTCAT*TTGAAATATAATAATACATCTGAAATAAATCCACTAG	+1 to +37	*CDR20291_0489*	OE
P1506 RP CDR20291_0489 pet16b BamHI	*CTTTGTTAGCAGCCGGATCC*TTATTCTTTTGACACAGTTTGTATCTTGCTG	+2208 to +2238	*CDR20291_0489*	OE
P1499 FP CDR20291_3482 pet16b NdeI	*ATATCGAAGGTCGTCAT*ATGAGTTATCAAAATTATAAATGTGAAAGAGATTATG	+1 to +37	*CDR20291_3482*	OE
P1498 RP CDR20291_3482 pet16b BamHI	*CTTTGTTAGCAGCCGGATCC*TTATACTAAAGGTGCTTCTATTTGAGTAGTAAAAAG	+370 to +405	*CDR20291_3482*	OE
P1505 FP CDR20291_0945 pet16b NdeI	*ATATCGAAGGTCGTCAT*ATGGATATTAAAGAAATAACAAAGAGTTATAAAGAC	+1 to +36	*CDR20291_0945*	OE
P1504 RP CDR20291_0945 pet16b BamHI	*CTTTGTTAGCAGCCGGATCC*TTATTTATTTAAGAAATCCACTGCATATTGCAC	+1132 to +1164	*CDR20291_0945*	OE
P1503 FP CDR20291_1811 pet16b NdeI	*ATATCGAAGGTCGTCAT*ATGTTTAAAAAAATAGTCGCAATAGCAAGCTC	+1 to +32	*CDR20291_1811*	OE
P1502 RP CDR20291_1811 pet16b BamHI	*CTTTGTTAGCAGCCGGATCC*TTATTTAATATTTTTTATAGCATTGTTTGCAAACTCAG	+1013 to +1050	*CDR20291_1811*	OE
P1511 FP *cdeC* pet16b NdeI	*ATATCGAAGGTCGTCAT*ATGCAAGATTATAAAAAAAATAAAAGAAGAATGATGAATC	+1 to +40	*cdeC*	OE
P1510 RP *cdeC* pet16b BamHI	*CTTTGTTAGCAGCCGGATCC*CTATCTGTGGCAACTTGGCTTTCC	+1195 to +1218	*cdeC*	OE
P1489 FP *sipL* pet16b NdeI	*ATATCGAAGGTCGTCAT*ATGGAATTAATTAAAGATGTAATTAAAGTTGACAATAG	+1 to +38	*sipL*	OE
P1488 RP *sipL* pet16b BamHI	*CTTTGTTAGCAGCCGGATCC*TTAATCTACTAATACGACTTTTTTTTCTAAAATAAGAC	+1514 to +1551	*sipL*	OE
P1509 FP CDR20291_0316 pet16b NdeI	*ATATCGAAGGTCGTCAT*ATGAGTAACAAGAAAAAAAAGGATTTAGATACTAG	+1 to +35	*CDR20291_0316*	OE
P1508 RP CDR20291_0316 pet16b BamHI	*CTTTGTTAGCAGCCGGATCC*TTACATTTCTTCCTCATCAAATTCTTCAATATTAG	+815 to +849	*CDR20291_0316*	OE
P1491 FP *cotJC1* pet16b NdeI	*ATATCGAAGGTCGTCAT*ATGTGGATTTATCAAAAAACACTGGAACATCC	+1 to +32	*cotJC1*	OE
P1490 RP *cotJC1* pet16b BamHI	*CTTTGTTAGCAGCCGGATCC*TTAGAACTGATGCTTACACTCTGTAAACTC	+544 to +573	*cotJC1*	OE
P1501 FP CDR20291_2028 pet16b NdeI	*ATATCGAAGGTCGTCAT*ATGAGTGATTTTAAAGAAATTTTGGCAGAAG	+1 to +31	*CDR20291_2028*	OE
P1500 RP CDR20291_2028 pet16b BamHI	*CTTTGTTAGCAGCCGGATCC*CTAATCTTCTTTTACAAGTACATTGGTTATTTC	+481 to +513	*CDR20291_2028*	OE

[a]Overlapping sequence for Gibson cloning is in italic and grey.

[b]The nucleotide position numbering begins from the first base pair of the ORF for each respective gene sequence.

[c]OE, overexpression of target gene.

Table S3. Plasmids used

Plasmid	Relevant characteristic	Source/Reference
pET16b	Vector carries an N-terminal His•Tag® sequence followed by a Factor Xa site and three cloning sites. Gene expression is under control of the T7lac promoter and is induced by IPTG addition.	Novagen
pET16b-*cdeM*	pET16b plasmid contains a 10xHis affinity tag, Factor Xa cleavage site followed by multiple cloning site with BamHI and NdeI restriction site. The *cdeM* gene (495bp) was amplified with primers 1486 and 1487, and cloned using Gibson Cloning between BamHI and NdeI restriction sites	This work
pET16b-*sipL*	pET16b plasmid contains a 10xHis affinity tag, Factor Xa cleavage site followed by multiple cloning site with BamHI and NdeI restriction site. The *sipL* gene (1551bp) was amplified with primers 1488 and 1489, and cloned using Gibson Cloning between BamHI and NdeI restriction sites	This work
pET16b-*cotJC1*	pET16b plasmid contains a 10xHis affinity tag, Factor Xa cleavage site followed by multiple cloning site with BamHI and NdeI restriction site. The *cotJC1* gene (573bp) was amplified with primers 1490 and 1491, and cloned using Gibson Cloning between BamHI and NdeI restriction sites	This work
pET16b-*cotE*	pET16b plasmid contains a 10xHis affinity tag, Factor Xa cleavage site followed by multiple cloning site with BamHI and NdeI restriction site. The *cotE* gene (2130bp) was amplified with primers 1492 and 1493, and cloned using Gibson Cloning between BamHI and NdeI restriction sites	This work
pET16b-*cotB*	pET16b plasmid contains a 10xHis affinity tag, Factor Xa cleavage site followed by multiple cloning site with BamHI and NdeI restriction site. The *cotB* gene (915bp) was amplified with primers 1494 and 1495, and cloned using Gibson Cloning between BamHI and NdeI restriction sites	This work
pET16b-*cotA*	pET16b plasmid contains a 10xHis affinity tag, Factor Xa cleavage site followed by multiple cloning site with BamHI and NdeI restriction site. The *cotA* gene (918bp) was amplified with primers 1496 and 1497, and cloned using Gibson Cloning between BamHI and NdeI restriction sites	This work
pET16b-CDR20291_3482	pET16b plasmid contains a 10xHis affinity tag, Factor Xa cleavage site followed by multiple cloning site with BamHI and NdeI restriction site. The CDR20291_3482 gene (405bp) was amplified with primers 1498 and 1499, and cloned using Gibson Cloning between BamHI and NdeI restriction sites	This work
pET16b-CDR20291_2028	pET16b plasmid contains a 10xHis affinity tag, Factor Xa cleavage site followed by multiple cloning site with BamHI and NdeI restriction site. The CDR20291_2028 gene (513bp) was amplified with primers 1500 and 1501, and cloned using Gibson Cloning between BamHI and NdeI restriction sites	This work
pET16b-CDR20291_1811	pET16b plasmid contains a 10xHis affinity tag, Factor Xa cleavage site followed by multiple cloning site with BamHI and NdeI restriction site. The CDR20291_1811 gene (1050bp) was amplified with primers 1502 and 1503, and cloned using Gibson Cloning between BamHI and NdeI restriction sites	This work
pET16b-CDR20291_0945	pET16b plasmid contains a 10xHis affinity tag, Factor Xa cleavage site followed by multiple cloning site with BamHI and NdeI restriction site. The CDR20291_0945 gene (1164bp) was amplified with primers 1504 and 1505, and cloned using Gibson Cloning between BamHI and NdeI restriction sites	This work
pET16b-CDR20291_0489	pET16b plasmid contains a 10xHis affinity tag, Factor Xa cleavage site followed by multiple cloning site with BamHI and NdeI restriction site. The CDR20291_0489 gene (2238bp) was	This work

	amplified with primers 1506 and 1507, and cloned using Gibson Cloning between BamHI and NdeI restriction sites	
pET16b-CDR20291_0316	pET16b plasmid contains a 10xHis affinity tag, Factor Xa cleavage site followed by multiple cloning site with BamHI and NdeI restriction site. The CDR20291_0316 gene (849bp) was amplified with primers 1508 and 1509, and cloned using Gibson Cloning between BamHI and NdeI restriction sites	This work
pET16b-*cdeC*	pET16b plasmid contains a 10xHis affinity tag, Factor Xa cleavage site followed by multiple cloning site with BamHI and NdeI restriction site. The *cdeC* gene (1218bp) was amplified with primers 1510 and 1511, and cloned using Gibson Cloning between BamHI and NdeI restriction sites	This work

References

1 Pizarro-Guajardo, M., Ravanal, M. C., Paez, M. D., Callegari, E. & Paredes-Sabja, D. Identification of Clostridium difficile Immunoreactive Spore Proteins of the Epidemic Strain R20291. *Proteomics Clin Appl* **12**, e1700182 (2018). https://doi.org:10.1002/prca.201700182

2 Wu, Y., Li, Q. & Chen, X. Z. Detecting protein-protein interactions by Far western blotting. *Nat Protoc* **2**, 3278-3284 (2007). https://doi.org:10.1038/nprot.2007.459

3 Gibson, D. G. *et al.* Enzymatic assembly of DNA molecules up to several hundred kilobases. *Nat Methods* **6**, 343-345 (2009). https://doi.org:10.1038/nmeth.1318

4 Birnboim, H. C. & Doly, J. A rapid alkaline extraction procedure for screening recombinant plasmid DNA. *Nucleic Acids Res* **7**, 1513-1523 (1979). https://doi.org:10.1093/nar/7.6.1513

5 Sambrook, J. & Russell, D. W. Purification of nucleic acids by extraction with phenol:chloroform. *CSH Protoc* **2006** (2006). https://doi.org:10.1101/pdb.prot4455

6 Castro-Córdova, P. *et al.* Redistribution of the Novel Clostridioides difficile Spore Adherence Receptor E-Cadherin by TcdA and TcdB Increases Spore Binding to Adherens Junctions. *Infect Immun* **91**, e0047622 (2023). https://doi.org:10.1128/iai.00476-22

7 Mora-Uribe, P. *et al.* Characterization of the Adherence of. *Front Cell Infect Microbiol* **6**, 99 (2016). https://doi.org:10.3389/fcimb.2016.00099

8 Xue, Q. *et al.* Bacillus anthracis spore entry into epithelial cells is an actin-dependent process requiring c-Src and PI3K. *PLoS One* **5**, e11665 (2010). https://doi.org:10.1371/journal.pone.0011665

9 Guerrero-Araya, E., Cid-Rojas, F., Muñoz, M., Rodríguez, C. & Paredes-Sabja, D. Identification of Novel Cryptic and Classical Clades in Clostridioides difficile. *bioRxiv*, 2025.2008.2001.668003 (2025). https://doi.org:10.1101/2025.08.01.668003

10 Knight, D. R. *et al.* Major genetic discontinuity and novel toxigenic species in Clostridioides difficile taxonomy. *Elife* **10** (2021). https://doi.org:10.7554/eLife.64325

11 Stadler, V. *et al.* Combinatorial synthesis of peptide arrays with a laser printer. *Angew Chem Int Ed Engl* **47**, 7132-7135 (2008). https://doi.org:10.1002/anie.200801616

12 Iwasaki, K., Goto, Y., Katoh, T. & Suga, H. Selective thioether macrocyclization of peptides having the N-terminal 2-chloroacetyl group and competing two or three cysteine residues in translation. *Org Biomol Chem* **10**, 5783-5786 (2012). https://doi.org:10.1039/c2ob25306b

13 Abramson, J. *et al.* Accurate structure prediction of biomolecular interactions with AlphaFold 3. *Nature* **630**, 493-500 (2024). https://doi.org:10.1038/s41586-024-07487-w

14 Pettersen, E. F. *et al.* UCSF ChimeraX: Structure visualization for researchers, educators, and developers. *Protein Sci* **30**, 70-82 (2021). https://doi.org:10.1002/pro.3943

15 Josuran, R. *Prot pi | Bioinformatics Calculator*, <https://www.protpi.ch/Calculator/ProteinTool> (2014).

16 Kyte, J. & Doolittle, R. F. A simple method for displaying the hydropathic character of a protein. *J Mol Biol* **157**, 105-132 (1982). https://doi.org:10.1016/0022-2836(82)90515-0

17 Buchan, D. W. A. & Jones, D. T. The PSIPRED Protein Analysis Workbench: 20 years on. *Nucleic Acids Res* **47**, W402-W407 (2019). https://doi.org:10.1093/nar/gkz297

18 Cid-Rojas, F.; Paredes-Sabja, D. Characterization of a hypersporulating strain derivative of *Clostridioides difficile* R20291. *bioRxiv* **2025**, 2025.2011.2025.690273.
